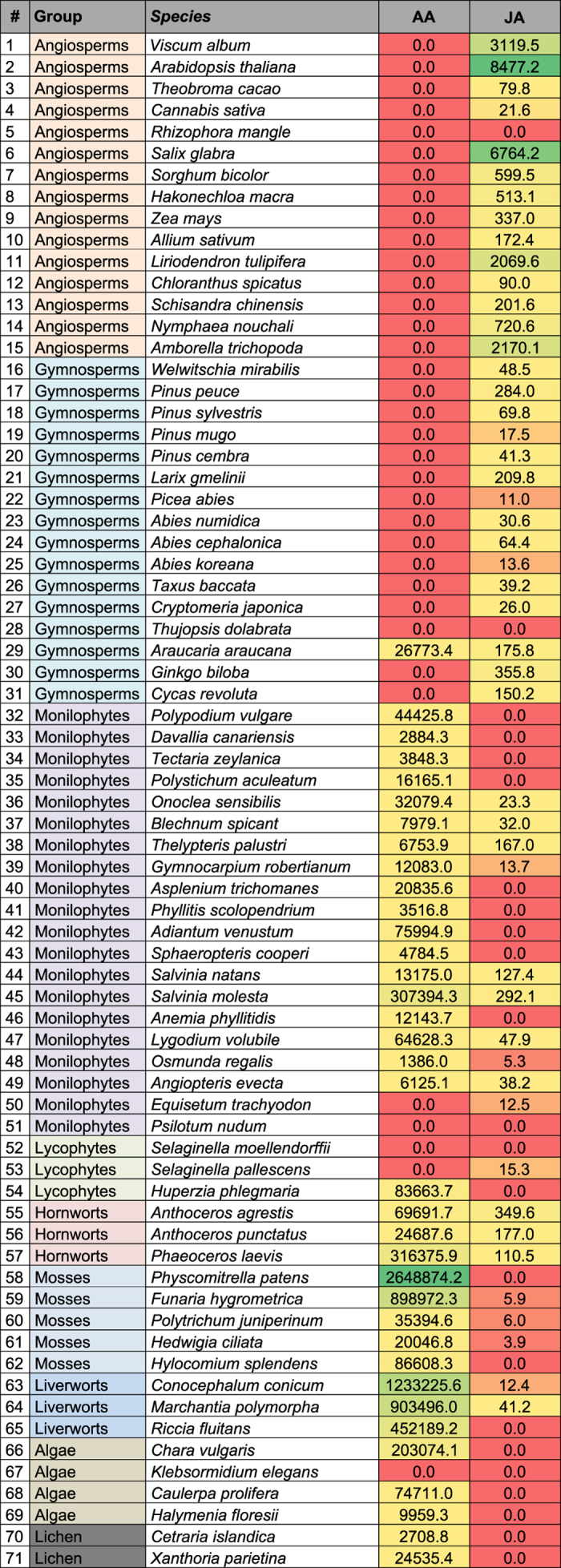# Targeted metabolomics shows plasticity in the evolution of signaling lipids and uncovers old and new endocannabinoids in the plant kingdom

**DOI:** 10.1038/srep41177

**Published:** 2017-01-25

**Authors:** María Salomé Gachet, Alexandra Schubert, Serafina Calarco, Julien Boccard, Jürg Gertsch

**Affiliations:** 1 Institute of Biochemistry and Molecular Medicine, NCCR TransCure, University of Bern, Bühlstrasse 28, 3012 Bern, Switzerland; 2 School of Pharmaceutical Science, University of Geneva, University of Lausanne, 1 rue Michel Servet, 1211 Geneva 4, Switzerland

## Abstract

The remarkable absence of arachidonic acid (AA) in seed plants prompted us to systematically study the presence of C20 polyunsaturated fatty acids, stearic acid, oleic acid, jasmonic acid (JA), *N*-acylethanolamines (NAEs) and endocannabinoids (ECs) in 71 plant species representative of major phylogenetic clades. Given the difficulty of extrapolating information about lipid metabolites from genetic data we employed targeted metabolomics using LC-MS/MS and GC-MS to study these signaling lipids in plant evolution. Intriguingly, the distribution of AA among the clades showed an inverse correlation with JA which was less present in algae, bryophytes and monilophytes. Conversely, ECs co-occurred with AA in algae and in the lower plants (bryophytes and monilophytes), thus prior to the evolution of cannabinoid receptors in Animalia. We identified two novel EC-like molecules derived from the eicosatetraenoic acid juniperonic acid, an omega-3 structural isomer of AA, namely juniperoyl ethanolamide and 2-juniperoyl glycerol in gymnosperms, lycophytes and few monilophytes. Principal component analysis of the targeted metabolic profiles suggested that distinct NAEs may occur in different monophyletic taxa. This is the first report on the molecular phylogenetic distribution of apparently ancient lipids in the plant kingdom, indicating biosynthetic plasticity and potential physiological roles of EC-like lipids in plants.

Lipids are not only a source of energy and physical protection, but also play a crucial role in the regulation of membrane fluidity and signal transduction^1^. Plant cell membranes can serve as reservoirs for biologically active lipid metabolites including fatty acids (FAs) that are released to act as ligands for particular receptors^2^. Given the emerging relevance of lipid signaling in plants^3^, a better understanding of the distribution of potentially functional lipids may shed light on how lipid-mediated signal transduction evolved in the plant kingdom. It has been shown that very long-chain fatty acids (VLCFA), which include the polyunsaturated fatty acid (PUFA) arachidonic acid (AA, 20:4, ω-6), are characteristic of algae, mosses and ferns^4^,^5^,^6^,^7^,^8^,^9^,^10^. On the other hand, flowering plants produce long-chain fatty acids (LCFA) with a chain length of C16 and C18 and zero to three *cis* double bounds (e.g., linoleic acid (LA, 18:2, ω-6) and *α*-linolenic acid (ALA, ω-3)^11^. However, to date no systematic analysis of AA and related eicosanoids in the plant kingdom has been reported. In mammals, AA is essential and can be obtained directly from the diet or it is synthesized from LA through successive desaturation (Δ6-desaturase), elongation (Δ6-elongase) and desaturation (Δ5-desaturase) reactions^12^ (Fig. 1). Intriguingly, angiosperms do not appear to produce AA, but residual long-chain FAs (≥C20 as components of lipid polymers such as suberin^13^) or shorter saturated and unsaturated FAs as constituents of phospholipids (PL)^1^, triglycerides (TG)^14^ or sterol esters^15^. Thus, although Δ6- and Δ5-desaturases are clearly present in higher plants^16^,^17^,^18^,^19^,^20^,^21^,^22^, these enzymes do not generally mediate the production of AA in seed plants. Nevertheless, angiosperms (e.g., *Arabidopsis thaliana*) seem to have evolved the capacity to perceive and respond to AA, which is produced by biotic invaders such as fungi, acting via well-known plant signaling pathways (i.e., jasmonic acid (JA) and salicylic acid pathways)^23^. Moreover, free FAs in plants are associated with signaling processes that elicit effects of their own (e.g., oleic acid (OA)^24^ and ALA^2^ promote disease resistance) or they are used in the production of bioactive metabolites such as oxylipins (oxygenated lipid metabolites) like JA. JA is the most studied oxylipin in plants and is the precursor of a family of bioactive metabolites, which are commonly known as jasmonates and, are derived mainly ALA^25^. Jasmonates are important regulators in plant responses to stress (biotic and abiotic) as well as in plant development^25^.

Another important emerging class of signaling lipids derived from FAs are *N*-acylethanolamines (NAEs)^3^,^26^,^27^ represented by arachidonoyl ethanolamide (anandamide, AEA) which together with 2-arachidonoyl gycerol (2-AG) act as endogenous cannabinoids (endocannabinoids, ECs) in animals^28^. In plants, studies on the distribution and function of NAEs, which contain FA chain lengths of C12-C18 varying in saturation, have been mostly carried out on angiosperm seeds (both monocot and dicots)^29^,^30^. The role of NAEs in plants remains poorly studied, although Chapman *et al*. could shown that they are involved in pathogenic responses, ABA-mediated growth processes and oxylipins formation^27^,^29^,^30^. In mammals, ECs are involved in many physiological and pathological conditions and activate the cannabinoid receptors CB1 and CB2, as well as other receptors^28^,^31^. The ECs 2-AG (here referred to as 1/2-AG as it undergoes rapid isomerization to 1-AG at a rate dependent upon the pH and extraction conditions^32^) and AEA are generated from AA^28^,^31^. Although AEA has been postulated to occur in cocoa (*Theobroma cacao* L) together with other NAEs^33^, no systematic data is available on the phylogenetic distribution of ECs in the plant kingdom. Previous studies have addressed the evolution of the endocannabinoid system (ECS) and reported the wide distribution of ECs in animal taxa^34^,^35^,^36^. However, in the plant kingdom the current insights have been based on genetic analyses and only indirect insights regarding the evolution of ECs could be inferred.

Here, we systematically analyzed 71 plant species representative of the major phylogenetic clades using LC-MS/MS analyses for the presence of AA, ECs (AEA and 1/2-AG), prostaglandin E2 (PGE2, important eicosanoid derived from AA in Animalia^12^), the NAEs myristoyl ethanolamide, (MEA), oleoyl ethanlonamide (OEA), palmitoyl ethanolamide (PEA), steaoryl ethanolamide (SEA), and linoleoyl ethanolamide (LEA), selected FA precursors (stearic acid (STE, 18:0), OA, ALA), the signaling molecule JA, as well as certain FAs upstream from the ω-6 pathway (dihomo-*γ*-linolenic acid (DHGLA, 20:3, ω-6) and adrenic acid (AdA, 22:4, ω-6)) and the ω-3 pathway (docosahexaenoic acid (DHA, 22:6, ω-3). During the course of the investigation, we discovered novel EC-like molecules derived from juniperonic acid (JuA, 20:4, ω-3), a FA characteristic of gymnosperms^37^ (i.e., juniperoyl ethanolamide (JEA) and 2-juniperoyl glycerol (1/2-JG)). To provide additional support for this finding, the newly identified JEA and 1/2-JG were synthesized and a subset of 33 samples of plant species that included all gymnosperms, *Psilotum nudum, Equisetum trachyodon, Selaginella moellendorffii* and *S. pallescens*, together with plant samples that either contained AA, AEA and/or 1/2-AG or not, were re-analyzed by LC-MS/MS and GC-MS. Our study provides new insights into the occurrence and molecular phylogenetic distribution of eicosanoids, NAEs and related lipid secondary metabolites within the plant kingdom. Principal component analysis (PCA) of the targeted metabolic profiles was used to provide a global overview of the dataset, assess similarities between targeted metabolic profiles, and relate groupings of plant species to phylogeny, revealing an emerging evolutionary pattern of plasticity in the generation of lipid signaling molecules of largely unknown physiological importance.

## Results

### Targeted metabolomics to study evolutionary relationships of signaling lipids in plants

**Figure 1:**
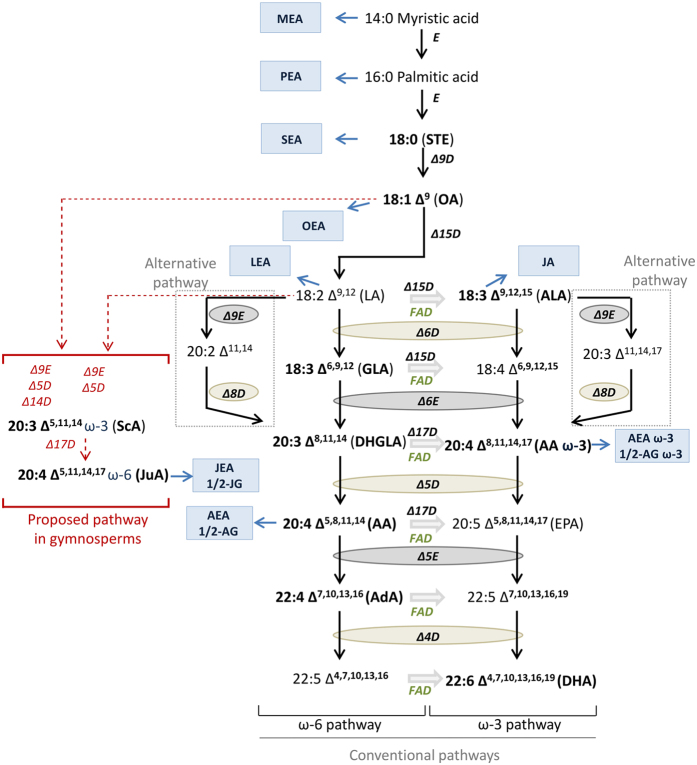
Conventional and alternative biosynthetic pathways for the production of PUFAs in eukaryotes. In green, the switch between ω-3 and ω-6 pathways characteristic of plants and nematodes through the action of ω-3 desaturases (FAD) is highlighted. Desaturases (*D*) and elongases (*E*) are shown together with the position at which the enzymatic reaction takes place. Red arrows highlight a proposed pathway for the biosynthesis of ScA and JuA in gymnosperms. For simplicity, the FA substrates here presented do not differentiate between acyl-lipid, acyl-ACP or acyl-CoA that occur either free or bounded to glycolipids. FAs investigated (bold) as well as the bioactive metabolites (blue squares pointed by blue arrows) that were investigated are shown.

**Figure 2:**
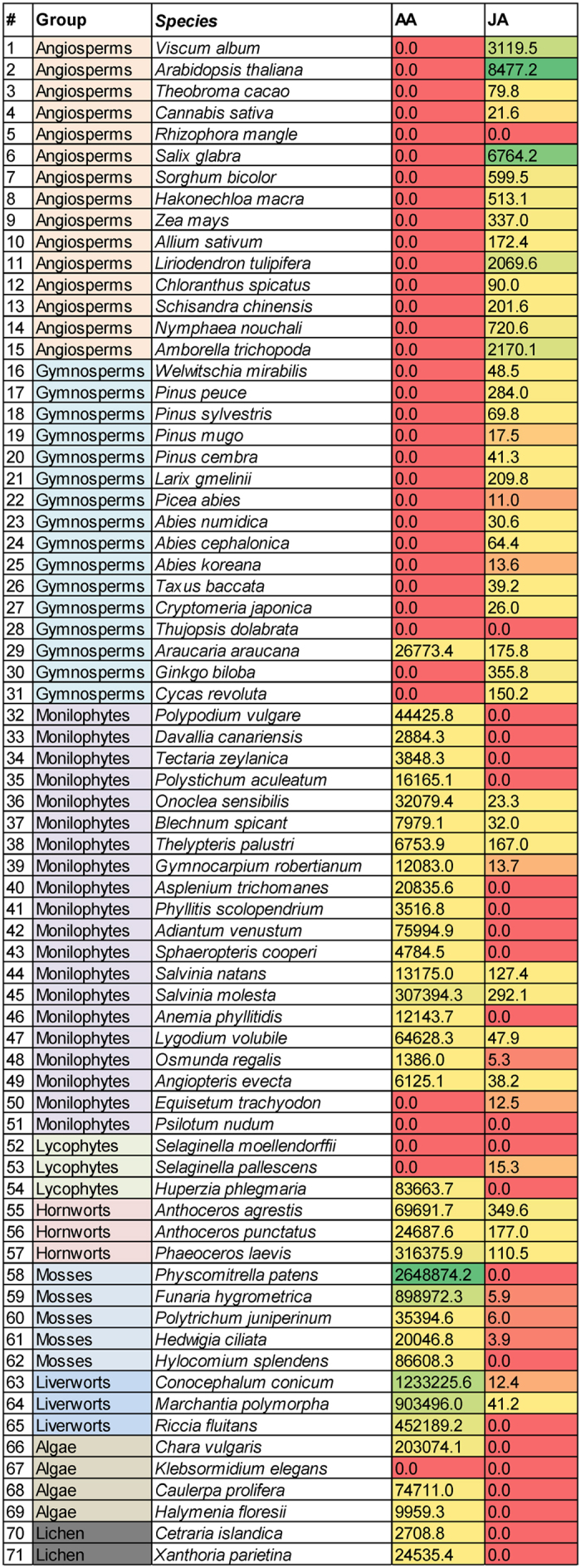
Concentrations (mean values) of AA and JA in pmol/g plant weight found in the 71 plant species analyzed (0.0 = < LOD). The background colors of the cells are used to highlight concentrations in the column (i.e. concentrations of the respective analyte are coded red for small amounts, yellow for intermediate amounts and green for high amounts). Complete information is available in Supplementary Tables S1 and S11.

In Fig. 1 (highlighted) and in Supplementary Fig. S1 (chemical structures), FAs as well as the lipid metabolites analyzed by LC-MS/MS and GC-MS are shown. Figure 1 depicts both conventional and alternative biosynthetic pathways of PUFAs in eukaryotes^38^,^39^,^40^ including a proposed pathway for the production of JuA in gymnosperms^41^ (*vide infra*). Sequence comparisons of the major enzymes involved in the biosynthesis of AA and/or degradation of ECs did not provide informative molecular phylogenetic relationships (Supplementary Excel File S1). Given the inherent difficulty of extrapolating information of lipid metabolites from genetic data and the high sequence similarities of anabolic and metabolic enzymes we employed targeted metabolomics to measure the presence of key FAs and related lipid metabolites. LC-MS/MS analyses of DCM extracts of leaves of 71 plant species revealed the presence (or absence) of AA, 1/2-AG, AEA, OA, STE, MEA, LEA, OEA, SEA, PEA, JA, ALA, DHGLA, AdA and DHA and allowed semi-quantitative analyses of their relative concentrations (Supplementary Tables S1–S15). OA, STE, LEA, OEA and ALA were quantified in all plant species investigated. An intriguing inverse phylogenetic correlation between AA and JA in lower versus higher plants was detected (Fig. 2), suggesting significant lower amounts of JA in taxa containing AA. As will be discussed below, AA and the ECs AEA and 1/2-AG were detected exclusively in the lower plants. PGE2/PGD2 (isomers with the same retention time in the analytical method used)^42^ was not detected in any of the samples analyzed.

### Identification of endocannabinoids and discovery of novel endocannabinoid-like lipids from juniperonic acid

**Figure 3:**
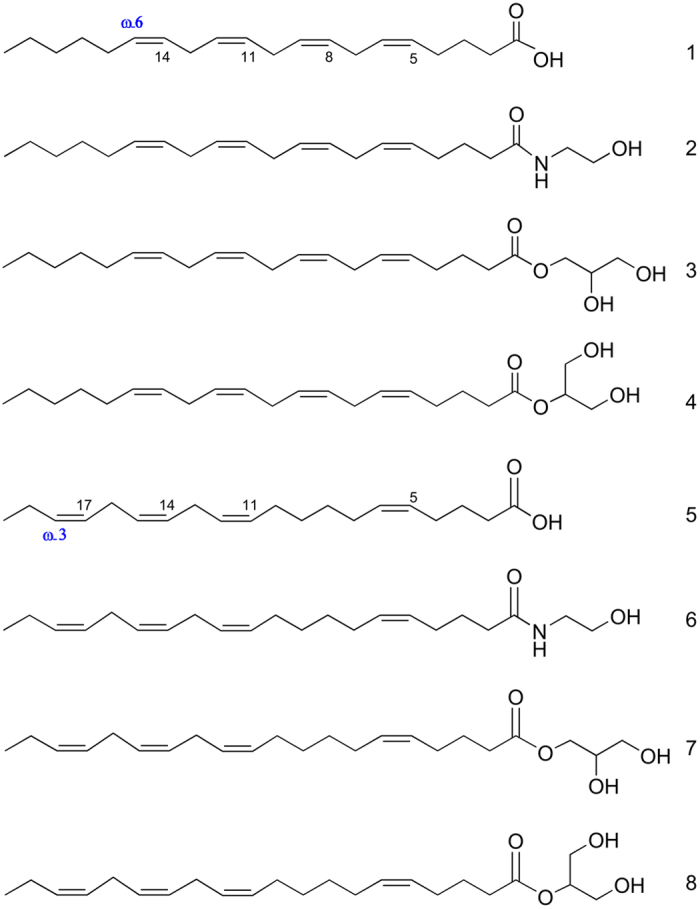
Chemical structures of C-20 PUFA metabolites. Arachidonic acid (**1**) (AA), arachidonoyl ethanolamide (AEA) (**2**), 1-arachidonoyl glycerol (1-AG) (**3**), 2-arachidonoyl glycerol (2-AG) (**4**), juniperonic acid (JuA) (**5**), juniperoyl ethanolamide (JEA) (**6**), 1-juniperoyl glycerol (1-JG) (**7**) and 2-juniperoyl glycerol (2-JG) (**8**).

**Figure 4:**
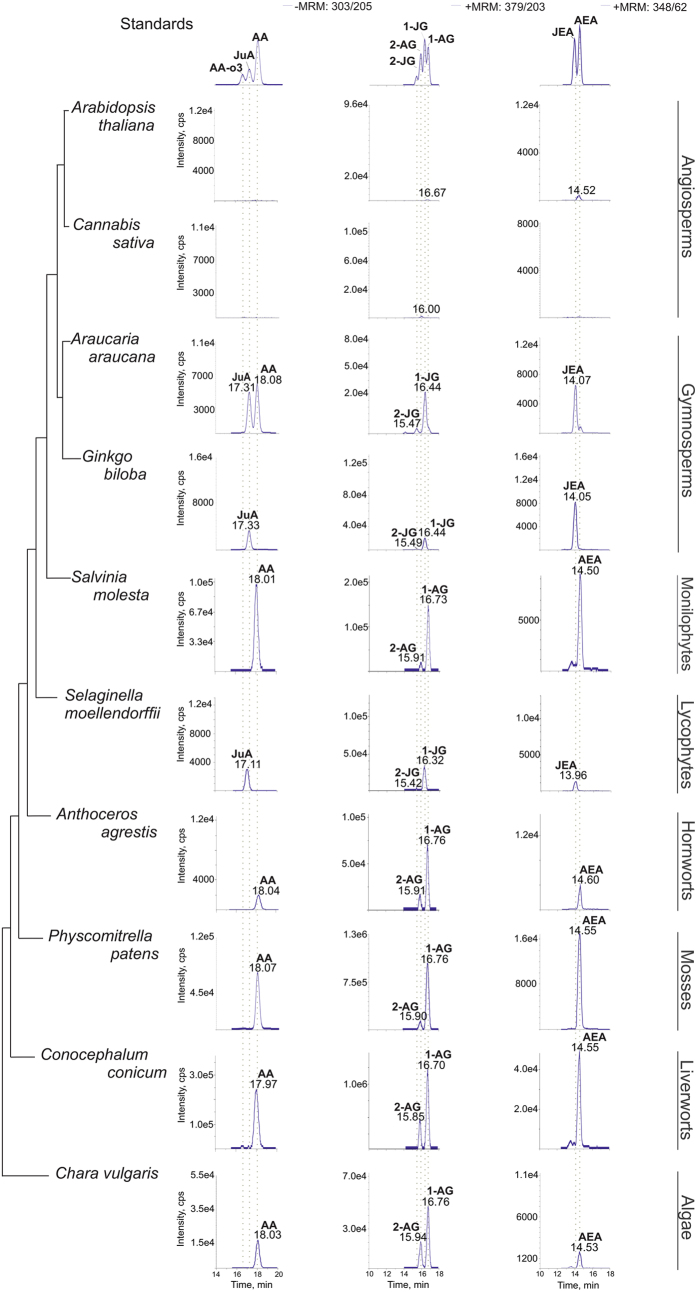
Chromatograms showing the analysis of C20 PUFA metabolites. First column: AA and JuA; second column: 1/2-AG and 1/2-JG and third column: AEA and JEA. Samples were prepared using HPLC for cleanup (see Sample preparation for identification) and analyzed using LC-MS/MS in the positive and negative modes (see Chromatographic conditions used for the identification of structural isomers (LC-MS/MS method 2)).

**Figure 5:**
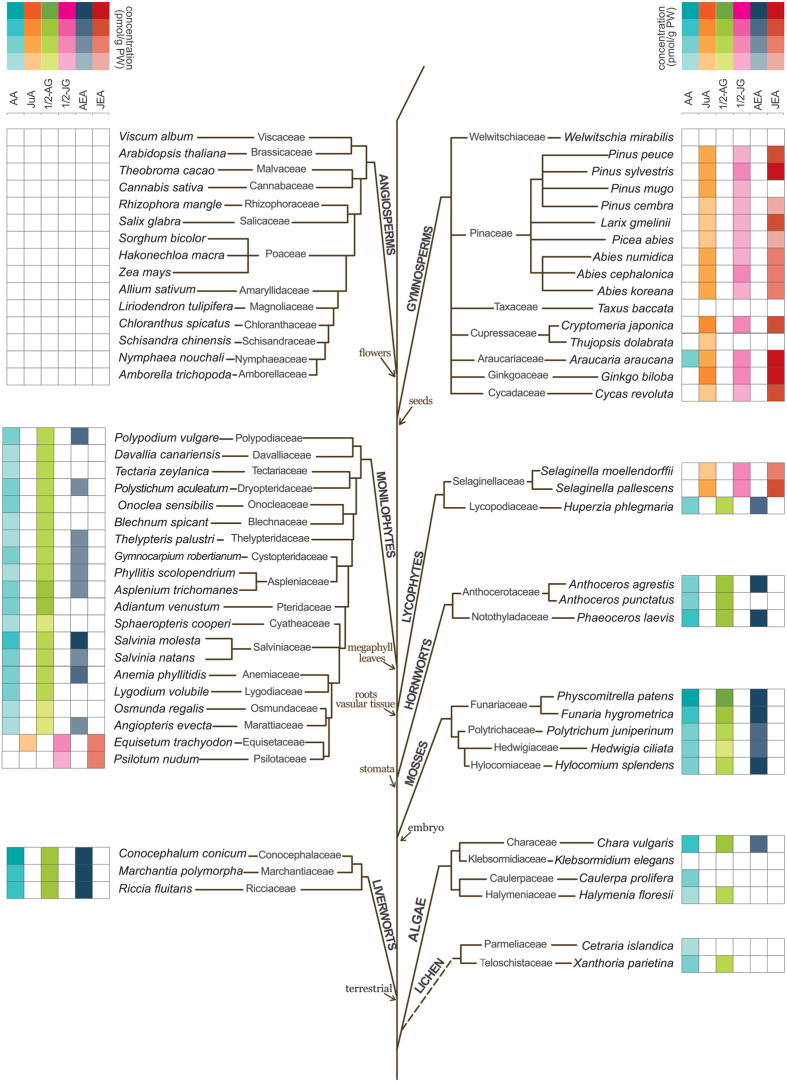
Chemotaxonomic distribution of C-20 PUFA metabolites. AA (pale blue), JuA (orange), 1/2-AG (green), 1/2-JG (pink), AEA (blue) and JEA (red). The intensity of the colors indicates concentration. The concentration ranges used for AA and JuA are 54 to 1E4 (shade 1), 1E4 to 1E5 (shade 2), 1E5 to 1E6 (shade 3) and >1E6 (shade 4); for 1/2-AG and 1/2-JG are 37 to 1E3 (shade 1), 1E3 to 1E4 (shade 2), 1E4 to 1E5 (shade 3) and >1E5 (shade 4); and for AEA and JEA are 0.8 to 1 (shade 1), 1 to 10 (shade 2), 10 to 100 (shade 3) and 100–1000 (shade 4).

Almost all gymnosperms (14/16: i.e., *Pinus peuce, Pinus sylvestris, Pinus mugo, Pinus cembra, Larix gmelinii, Picea abies, Abies numidica, A. cephalonica, A. koreana, Cryptomeria japonica, Thujopsis dolabrata, Araucaria araucana, Ginkgo biloba* and *Cycas revoluta*) and lycophytes (2/3: i.e., *Selaginella moellendorffii* and *S. pallescens*) investigated, as well as two monilophytes (2/20: i.e., *Psilotum nudum* and *Equisetum trachyodon*) contained AA and an as-yet unidentified compound that shares a high degree of similarity with AEA and 1/2-AG (see Supplementary Fig. S2). The enhanced product ion (EPI) spectra obtained from these samples generated similar spectra to 2-AG, suggesting the existence of an eicosatetranoyl glycerol metabolite. Based on this and taking into account the results of Wolff *et al*.^37^, who reported the presence of JuA in conifers, we synthesized the AEA and 1/2-AG analogues of JuA (i.e., JEA and 1/2-JG) to thoroughly investigate the presence of JuA derived EC-like molecules in gymnosperms. The chemical structures of AA, AEA, 1-AG, 2-AG, JuA, JEA, 1-JG and 2-JG are shown in Fig. 3. Using our synthetic JEA and 1/2-JG standards a subset of 33 plant species (see Table S1 in Supplementary Information), which included all critical plant samples together with samples that either contained AA, AEA and/or 1/2-AG or not, were re-analyzed using LC-MS/MS (Fig. 4 and Supplementary Fig. S3) and GC-MS (see Supplementary Figs S4–S6). As shown in Fig. 5, all critical samples, with the exception of *Araucaria araucana*, contained exclusively JuA and, in most cases, also JEA and/or 1/2-JG. Conversely, the plant samples that contained AA also contained AEA and/or 1/2-AG. Interestingly, *Araucaria araucana* contained both AA and JuA, but only JEA and 1/2-JG, suggesting that JuA is the preferred eicosatetraenoic acid substrate for EC biosynthesis in this taxon. Noteworthy, detectable levels of arachidonic acid ω-3 (AA ω-3, 20:4, ω-3) were found only in *Conocephalum conicum* and *Chara vulgaris* but EC-like molecules derived from AA ω-3 were not detected (data not shown).

### Distribution of *α*-linolenic acid, *γ*-linolenic acid (GLA), dihomo-*γ*-linolenic acid and sciadonic acid (ScA) in the plant kingdom

The analytical methods used for the re-analysis of the 33 plant species were also used to identify the structural isomers of ALA and DHGLA (i.e. GLA (18:3, ω-6) and ScA (20:3, ω6). ALA was almost exclusively present in these 33 samples re-analyzed. However, only low quantities of GLA was measured in *Pinus peuce* (of the total unassigned ALA/GLA, 7% corresponded to GLA), *Pinus sylvestris* (4%), *Pinus mugo* (10%), *Pinus cembra* (11%), *Larix gmelinii* (10%), *Picea abies* (3%), *Abies numidica* (1%), *Abies koreana* (5%), *Salvinia molesta* (3%), *Physcomitrella patens* (11%) and *Conocephalum conicum* (8%). Supplementary Table S12 shows the concentrations of ALA and GLA in 33 plants species and the unassigned ALA/GLA in the remaining 38 plant species investigated. Supplementary Fig. S7 shows chromatograms (LC-MS/MS and GC-MS) exemplifying their analyses. The analyses of DHGLA and ScA shows that all JuA containing plant species (Fig. 5) contained exclusively ScA, except for *Pinus mugo* (of the total of DHGLA:ScA, 6% corresponds to DHGLA and; 94%, to ScA), *Picea abies* (DHGLA:ScA - 7:93%), *Abies cephalonica* (DHGLA:ScA - 9:91%) and *Abies koreana* (DHGLA:ScA - 10:90%), which also contained low amounts of DHGLA. Conversly, AA containing plant species (Fig. 5) contained almost exclusively DHGLA (i.e., *Salvinia molesta* (DHGLA:ScA - 71:29%), *Huperzia phlegmaria* (DHGLA:ScA - 88:12%), *Physcomitrella patens* (DHGLA:ScA - 100:0%), *Hylocomium splendens* (DHGLA:ScA - 100:0%), *Conocephalum conicum* (DHGLA:ScA - 100:0%) and *Chara vulgaris* (DHGLA:ScA - 87:13%). Concentrations of these analytes are presented in Supplementary Table S13, while chromatograms (LC-MS/MS and GC-MS) are shown in Supplementary Fig. S8. Additionally, we investigated the samples for their contents of the VLFA AdA (22:4, ω-6) and DHA (22:6, ω-3) in the initial screening (71 plant species) and report their concentrations in 17 and 14 plant species, respectively (see Supplementary Tables S14 and S15). From these, more than 50% are mosses (5/5 mosses analyzed contained AdA and 4/5 DHA), liverworts (3/3 and 3/3) and algae (1/4 and 2/4), in agreement with the general knowledge that lower organism can produce VLFA. Based on the observation that the chromatographic conditions used during the quantification could not separate the structural isomers of AA/JuA/AA ω-3, ALA/GLA and DHGLA/ScA, we could not confirm the identities of AdA and DHA. Therefore, this information was not used in subsequent analyses.

### Principal component analysis reveals clade-specific plasticity in plant signaling lipids

**Figure 6:**
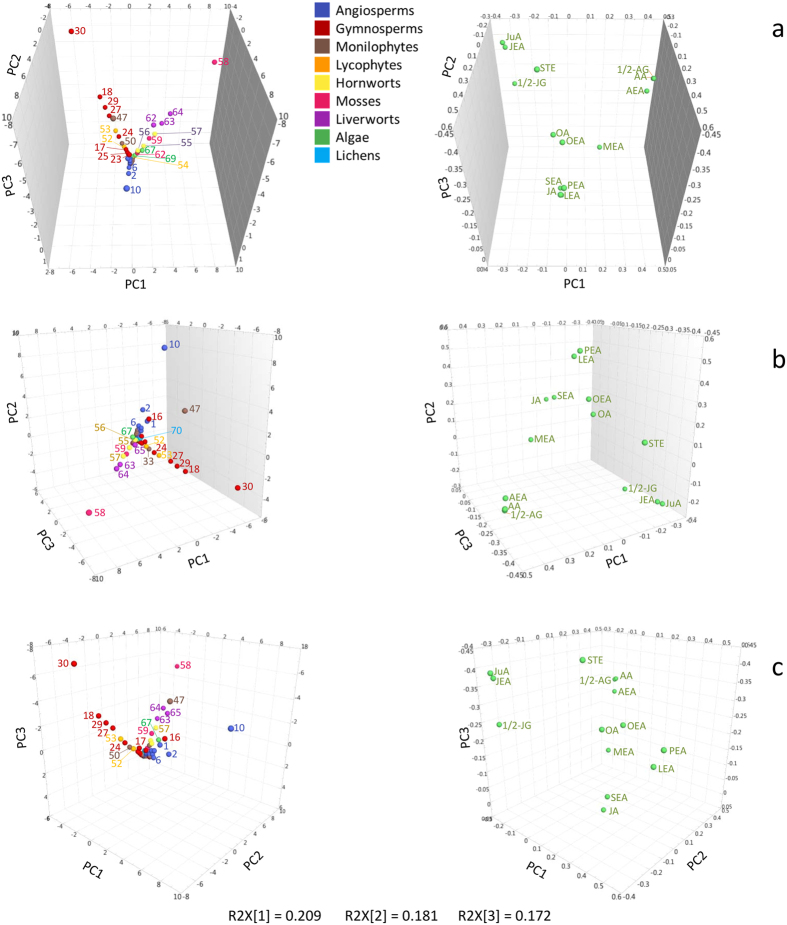
3D-PCA plots depicting 71 plant species distributed in the 9 plant groups investigated. Nine different colors are used to highlight the plant groups. Three panels (**a–c**) showing the same analysis in different positions are presented to highlight difference between plant groups and samples. Each panel shows the scores on the left and the loadings on the right sides, respectively. **Angiosperms:** 1) *Viscum album*, 2) *Arabidopsis thaliana*, 3) *Theobroma cacao*, 4) *Cannabis sativa*, 5) *Rhizophora mangle*, 6) *Salix glabra*, 7) *Sorghum bicolor*, 8) *Hakonechloa macra*, 9) *Zea mays*, 10) *Allium sativum*, 11) *Liriodendron tulipifera*, 12) *Chloranthus spicatus*, 13) *Schisandra chinensis*, 14) *Nymphaea nouchali*, 15) *Amborella trichopoda*; **Gymnospers:** 16) *Welwitschia mirabilis*, 17) *Pinus peuce*, 18) *Pinus peuce*, 19) *Pinus mugo*, 20) *Pinus cembra*, 21) *Larix gmelinii*, 22) *Picea abies*, 23) *Abies numidica*, 24) *Abies cephalonica*, 25) *Abies koreana*, 26) *Taxus baccata*, 27) *Cryptomeria japonica*, 28) *Thujopsis dolabrata*, 29) *Araucaria araucana*, 30) *Ginkgo biloba*, 31) *Cycas revoluta*; **Monilophytes:** 32) *Polypodium vulgare*, 33) *Davallia canariensis*, 34) *Tectaria zeylanica*, 35) *Polystichum aculeatum*, 36) *Onoclea sensibilis*, 37) *Blechnum spicant*, 38) *Thelypteris palustri*, 39) *Gymnocarpium robertianum*, 40) *Asplenium trichomanes*, 41) *Phyllitis scolopendrium*, 42) *Adiantum venustum*, 43) *Sphaeropteris cooperi*, 44) *Salvinia natans*, 45) *Salvinia molesta*, 46) *Anemia phyllitidis*, 47) *Lygodium volubile*, 48) *Osmunda regalis*, 49) *Angiopteris evecta*, 50) *Equisetum trachyodon*, 51) *Psilotum nudum*; **Lycophytes:** 52) *Selaginella moellendorffii*, 53) *Selaginella pallescens*, 54) *Huperzia phlegmaria*; **Hornworts:** 55) *Anthoceros agrestis*, 56) *Anthoceros punctatus*, 57) *Phaeoceros laevis*; **Mosses:** 58) *Physcomitrella patens*, 59) *Funaria hygrometrica*, 60) *Polytrichum juniperinum*, 61) *Hedwigia ciliata*, 62) *Hylocomium splendens*; **Liverworts:** 63) *Conocephalum conicum*, 64) Marchantia polymorpha, 65) *Riccia fluitans*; **Algae:** 66) *Chara vulgaris*, 67) *Klebsormidium elegans*, 68) *Caulerpa prolifera*, 69) *Halymenia floresii*; **Lichens:** 70) *Cetraria islandica*, 71) *Xanthoria parietina*.

To determine whether the 14 targeted metabolites measured (i.e., AA, AEA, 1/2-AG, JuA, JEA, 1/2-JG, JA, OA, STE, LEA, MEA, OEA, PEA and SEA) might be useful for chemotaxonomic purposes or to derive evolutionary hypotheses, principal component analysis (PCA) was performed (see Supplementary Table S16). To that aim, the first 3 principal components (PCs) that covered 56% of the total variability were used to display the major trends in the dataset (Fig. 6). The negative correlations observed between AA/1/2-AG/AEA and JuA/1/2-JG/JEA were the main contributors to the first PC (R2X[1]) summarizing 21% of the variability within the data (see Supplementary Table S17) as shown in Fig. 6a–c. The high levels of AA, 1/2-AG and AEA found in *Physcomitrella patens* (sample 58) clearly differentiate it from the other samples, while *Ginkgo biloba* (sample 30) can be distinguished, not only due to its content of JA, 1/2-JG and JEA, but also, and mainly, because of its higher levels of STE and JuA. In Fig. 6a and b, *Lygodium volubile* (sample 47) was separated from the other AA containing samples due to the high levels of STE and OA (Fig. 6c). STE > JuA > JEA > 1/2-AG > AA > AEA > OA are the main representatives of the third PC (R2X[3], summarizing 17% of the variability (see Supplementary Table S17)). Additionally, Fig. 6a shows a clear trend separating angiosperms from the other plant groups, not only due to the absence of JuA and AA (and their derivatives), but also due to the high levels of PEA, LEA and OEA contained in this plant group (i.e., the second PC (R2X[2]). This component accounting for approximately 18% of the variability is driven by PEA > LEA >  > OEA (see Supplementary Table S17)). The only three samples in which PEA levels were < LOD belong to the gymnosperm genus *Abies* (i.e. *A. numidica, A. cephalonica* and *A. koreana*). *Allium sativum* (sample 10), which contained high levels of LEA, PEA and OEA, is clearly separated from the other angiosperms evaluated. It would thus be interesting to more systematically compare monocots and dicots for these NAEs. In general, LEA, OEA and PEA are more abundant in the angiosperms > gymnosperms > monilophytes. Moreover, OA, STE, LEA and OEA were positively correlated and seem to be, in general, more abundant in angiosperms and less abundant in monilophytes (see Supplementary Table S16). This correlation is supported by examining the fourth PC (representing 14% of the variability, see Supplementary Table S17) which is driven by OA > SEA > SA > LEA > OEA. On the other hand, SEA seems to be more abundant in monilophytes (see Supplementary Table S16). Interestingly, the only two gymnosperms investigated, which lacked JuA (i.e., *Welwitschia mirabilis* and *Taxus baccata*) showed very different trends. *W. mirabilis* (sample 16) contained the highest concentration of OEA and did not group among the gymnosperms, but instead tended towards the angiosperms (Fig. 6b,c). Meanwhile, the *T. baccata* sample (sample 26), which contained high levels of SEA, was found closer to the monilophytes (see Supplementary Table S16).

Samples 10, 30, 47 and 58 (i.e., *Allium sativum, Ginkgo biloba, Lygodium volubile* and *Physcomitrella patens*) presented the most characteristic metabolic profiles, (Fig. 6a–c), representing prototypical examples of metabolic patterns associated with angiosperms, gymnosperms, monilophytes and mosses, respectively. Interestingly, *Equisetum trachyodon* (sample 50, Fig. 6a and c) seems to group with the gymnosperms rather than the monilophytes, possibly due to JuA, JEA and 1/2-JG.

Another result that is not immediately visible on the PCA 3D score plot can be extracted by analyzing the observation scores and variable loadings of higher components (see Supplementary Table S17). JA that contributes almost entirely to the fifth PC, was commonly detected in angiosperms > gymnosperms > monilophytes > hornworts > liverworts > lycophytes > mosses but not detected in algae and lichens. While MEA that almost exclusively contributed to the sixth PC, is found in high concentrations in hornworts, mosses and algae. In general, the distributions of JA and MEA were negatively correlated, overlapping in the monilophytes group, which shows low levels of both JA and MEA. Similarly, *Rhizophora mangle* (red mangrove) was the only angiosperm that lacked JA and showed low levels of MEA, while the sweet water algae *Klebsormidium elegans* displayed the highest levels of MEA, but its level of JA was < LOD. The three species investigated that grow in sea water (i.e., *Rhizophora mangle, Caulerpa prolifera* and *Halymenia floresii*) show JA levels < LOD.

## Discussion

The absence of the eicosatetraenoic acid AA in higher plants here reported is intriguing because this essential ω-6 PUFA exerts numerous fundamental functions in Animalia. Yet, plants generally seem to be able to respond to AA by activating a discrete signal transduction pathway^23^. The SOFA database reports the presence of AA as part of the seed oils of few angiosperms (e.g. *Nigella sativa, Persea americana* and *Ximenia americana*)^43^. However, some literature sources are not available or the analytical information presented in the references is ambiguous, lacking spectrometric resolution. Our data suggest that the biosynthesis of AA in plants evolved early and free AA is widely present in lichen (2/2), algae (3/4) and lower land plants (liverworts (3/3), mosses (4/4), hornworts (3/3) and monilophytes (18/20). The biosynthesis of the ω-3 eicosatetraenoic acid JuA seems to have evolved through later independent events (e.g., diversification or convergent evolution) primarily in gymnosperms and was lost in angiosperms. Not unexpectedly, strict associations between AA and ECs were found, showing that, in contrast to bryophytes and ferns, angiosperms do not seem to contain the ECs AEA and 1/2-AG. The presence of the major EC 1/2-AG in lower plants is reported for the first time here. As in animal tissues, 1/2-AG is also present in higher amounts in most AA containing plant species suggesting the presence of arachidonoyl harboring diacylglycerol precursors. Overall, the current study and previous reports show that AA is present in monilophytes, bryophytes and algae^4^,^5^,^6^,^7^,^8^,^9^,^10^. AA was previously identified in the genera *Polypodium* (leaves: 18.4–174.3 nmol/g dry weight vs. 44.4 nmol/g dry/fresh weight here reported)^7^, *Polystichum*^4^, *Adiantum*^4^, *Onoclea sensibilis*^4^, *Osmunda*^4^, *Hylocomium splendens*^4^, *Hedwigia ciliata*^4^, *Physcomitrella patens*^10^, *Marchantia polymorpha*^5^ and *Caulerpa*^9^. AA was also reported in *Allium sativum* bulbs^44^ but we could neither detect it in bulbs (data not shown) nor in leaves. However, we could confirm the presence of AA in *Araucaria araucana*^45^, though ECs were not detected. Considering that AA is present in algae and lower plants, different studies have attempted to identify PGE2^46^. We did not detect PGE2/PGD2 (isomers coming at the same retention time with the analytical method used)^42^ in any of the samples analyzed, in perfect agreement with the general absence of cyclooxygenases (COX-1 and -2) in plants^47^. Thus, it cannot be excluded that reports on PGE2 in the plant kingdom are analytical artefacts.

We have identified two novel EC-like molecules derived from JuA, namely JEA and 1/2-JG by employing a combination of chemical synthesis and mass spectrometric techniques. JEA and 1/2-JG were detected in gymnosperms (14/16 (absent from *Welwitschia mirabilis* and *Taxus baccata*)), two monilophytes (2/20 (i.e. *Psilotum nudum* and *Equisetum trachyodon*)) and two lycophytes of the same genus (2/3 (i.e., *Selaginella moellendorffii* and *S. pallescens*)). These new ECs co-occur with JuA, highlighting the biosynthetic relationships of AA with 1/2-AG and AEA (Fig. 5). ECs derived from AA ω-3 (we also synthesized AEA ω-3 and 1/2-AG ω-3 from AA ω-3 to perform LC-MS/MS measurements) were not detected (data not shown). Low amounts of AA ω-3 were found in the alga *Chara vulgaris* and in the liverwort *Conocephalum conicum.* Interestingly, AA ω-3 has been identified in several species of microalgae^8^ and in the gymnosperm *Agathis robusta*^48^. The shared presence of JuA derivatives in the lycophytes *Selaginella moellendorffii* and *Selaginella pallescens*, the monilophytes *Psilotum nudum* and *Equisetum trachyodon* and the gymnosperms appears to be evolutionarily significant and might suggest a potential compensatory role of this eicosatetraenoic acid for the loss of AA biosynthesis within this division. Despite the fact that only a limited number of lycophytes species were investigated, the detection of either AA or JuA clearly indicates chemotaxonomic connections between these monophyletic groups which common ancestor dates to around 420 million years ago (Mya)^49^,^50^. Noteworthy, in our analysis, *A. araucana* was the only plant species in which both AA and JuA were detected. We may thus hypothesize that the diversification of metabolites in different lineages has taken place upon a transition in desaturase substrate specificity based on unknown selection pressures. Both AA and JuA require Δ5-desaturase for their biosynthesis (Fig. 1). In plants, Δ5-desaturase has been identified in the moss *Physcomitrella patens*^51^ and in the fresh water algae *Parietochloris incisa*^52^ as being crucial in the final step of the biosynthesis of AA. Furthermore, Δ5-desaturase has been recognized to be important in an alternative Δ8-desaturation pathway involved in the production of AA and JuA^53^ and to be involved in the synthesis of ScA, the precursor of JuA in *Anemone leveillei* (Ranunculaceae)^21^. Interestingly, Δ5-desaturase isolated from *Limnanthes douglasii* (Brassicaceae) uses different saturated FAs as substrates (16:0 > 18:0 = 20:0 > 22:0)^18^, again revealing substrate plasticity of plant desaturases. Several studies have reported that JuA is present in the seeds of several species of gymnosperms (including *Pinus, Taxus, Cryptomeria, Araucaria, Abies, Picea* and *Larix*)^43^, demonstrating that this peculiar, unsaturated, ω-3 FA is characteristic of gymnosperms. However, information about the content of JuA in leaves has only been reported for species of *Araucaria*^45^ and *Ginkgo biloba*^4^. In agreement with our findings, Wolff *et al*. detected both AA and JuA in *Araucaria* species^45^. Our data shows that *Araucaria araucana* contains AA, JuA and ScA, while *Taxus baccata* and *Welwitschia mirabilis* contain none of these and thus differ from the other gymnosperms samples investigated (all of which contained JuA and ScA). The diversification of gymnosperms such as Gnetidae (including Welwitschiaceae), Pinidae (including Araucariaceae, Pinaceae, Taxaceae, Cupressaceae), Ginkgoidae (Ginkgoaceae) and Cycadidae (including Cycadaceae) has been dated to have occurred between 330 Mya with the appearance of seed plants (Carboniferous period) and 125 Mya with the occurrence Gnetidae (Cretaceous period), which overlaps with the arrival of the first angiosperms, dated to approximately 194 Mya (Jurassic period)^50^. The exact phylogenetic relationships among seed plants (angiosperms and gymnosperms) are still unclear due to conflicting theories generated from analyses of molecular and morphological data^54^.

Our results may indicate chemotaxonomic relationships between *Welwitschia mirabilis* (Gnetatae) and the angiosperms (Fig. 6b,c), while *Ginkgo biloba, Cycas revoluta* and the investigated representatives of Pinidae (Pinaceae (9/16), Cupressaceae (2/16), Araucariaceae (1/16)) except for Taxaceae (1/16)) share chemotaxonomic lipid markers (Fig. 6a,c). ScA and JuA have also been reported in *Equisetum* species^4^,^43^ but, to our knowledge this is the first time that they have been identified in *Psilotum nudum* and *Selaginella* species. Nevertheless, the last common ancestor of the euphyllophytes, which includes the spermatophytes (seed plants) and monilophytes (ferns), has been dated from the Devonian period (around 392 Mya), whereby the Lycophytes also diversified around 384 Mya^50^. The roles of AA and JuA in plants are currently unknown, but both lipids might have similar functions. To our knowledge, for the first time we show that plants containing AA and JuA also have the biosynthetic capability to synthesize corresponding ECs, even though they are known to produce other NAEs^27^,^29^, and express homologs of the fatty acid amide hydrolase^55^,^56^. FAAH is the principal enzyme responsible for the degradation of AEA in mammals and NAEs in general, and in plants this enzyme exerts biochemical functions similar to that found in animals^56^ (*vide infra*). The presence of AEA in chocolate (assuming that it originated from *Theobroma cacao* beans)^33^ and the recent finding of its occurrence in the Ascomycota *Tuber melanosporum* (black truffle)^57^ are hitherto the only reports of AEA to date from non-animal organisms. To date, the occurrence of 2-AG in plants has not yet been reported, although a 2-AG analogue derived from ScA has been detected in the seeds of the conifer *Sciadopitys verticillata*^58^.

Even though angiosperms have lost the ability (or necessity) to produce long-chain PUFAs such as AA or JuA they can detect AA that is produced by pathogens and activate plant defense responses^23^. It will thus be interesting to elucidate the corresponding lipid receptors in plants and investigate whether these signal transduction events differ between distinct PUFAs. For instance, NAEs (*N*-lauroylethanolamine, MEA, PEA, SEA, OEA, LEA and *N-α*-linolenoylethanolamine) are widespread in plants but have been mainly reported from certain angiosperms and mostly detected in the seeds of crop plants (12 species, such as tomato, corn, cotton) or legumes (14 species, such as *Lupinus* spp., *Medicago* spp., *Pisum* spp.), none of which were included in our analysis. The concentration ranges of NAEs obtained in seeds were highly variable according to the species (i.e., MEA ranged from 13.3–1882 pmol/g fresh weight; PEA, from 125–43478 pmol/g fresh weight; SEA, from 23–6208 pmol/g; OEA, from 75–29538 pmol/g; and LEA, from 89–39443 pmol/g fresh weight)^30^,^59^. Information for NAEs in vegetative tissue have only been described for *A. thaliana*, with levels of MEA, PEA, SEA and OEA < LOD^30^.

JA has been identified to be a precursor of more than 20 metabolites^60^, the final biosynthetic product of which is (+)-7-iso-JA, although some conditions favor the formation of its stereoisomer (−)-JA (1:9)^61^. The JA levels found in plants depend on the tissue investigated, the developmental stage of the plant and external stimuli^60^. Many studies have reported the content of JA in fresh plant tissue. Recent investigations using LC-MS/MS analyses have reported JA levels in fresh leaves of angiosperms^62^,^63^,^64^,^65^ including *Zea mays* (ca. 306 pmol/g fresh weight^62^ versus the 337 pmol/g dry weight reported here) and *Arabidopsis thaliana (*ca. 84 pmol/g fresh weight^62^ versus the 8477 pmol/g dry weight reported here). In agreement with our data, in bryophytes analyses of 6 liverworts and 24 mosses showed low levels of JA that ranged from 1.2–78.8 pmol/g^66^ and JA was not detected in the moss *Physcomitrella patens*^67^.

**Figure 7:**
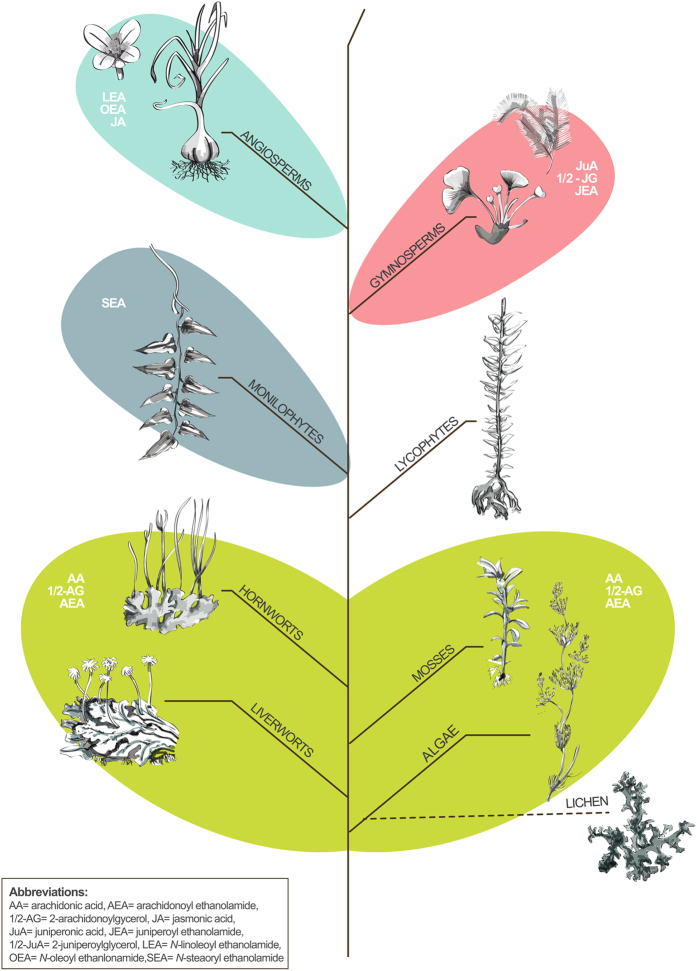
Graphical representation of the metabolite distribution within the investigated phylogenetic groups.

Previous metabolomics studies have successfully used FA fingerprints to investigate chemotaxonomic differences in gymnosperms^68^, Pinidae^37^, Pinaceae^69^ and microalgae^8^. From a chemotaxonomic perspective, our data confirm that monilophytes and lycophytes represent transition groups between seed plants and lower plants. Even though the number of samples belonging to the phylogenetic groups of “lower plants” was limited, we could identify significant trends with regard to the lipid metabolites investigated, and shared chemotaxonomic markers between the two lichen samples studied and algae. A clear limitation of the present study is the lack of knowledge about the distribution of the lipids among different plant organs. Nevertheless, the use of vegetative tissue can serve as a comparative starting point and we hypothesize that the apparent clade-specific differences of signaling lipids observed in this study may reflect evolutionary plant physiological processes. Additionally, care must be taken when drawing conclusions regarding mean concentrations of the metabolites investigated as large variations for most plant species and most analytes were observed (semiquantitative approach). This might be due to sample preparation (enzymatic processes during drying), plant specific matrix effects during LC-MS/MS analyses and when analyzing two or more different plant samples of the same species the variations could be either intrinsic of the plant or the collection (circadian rhythm, season, etc.). However, general evolutionary patterns of these signaling lipids within the plant kingdom can be postulated (Fig. 7). Based on the discovery of known and novel EC-like lipids in plants we conclude that ethanolamine and glycerol derivatives of the different eicosatetraenoic acids (AA, JuA but also ScA) relatively widely occur in Cryptogamae and gymnosperms with as yet unknown biological roles. Thus, the major EC in mammals is also a major glycerol derivative in lower plants and clearly evolved prior to CB receptors in Animalia. In a future study we will assess the interaction of the JuA-derived endocannabinoid-like molecules with CB receptors and endocannabinoid degrading enzymes. Overall, it will be interesting to elucidate the biological and evolutionary reasons for the likely absence of AA and JuA in angiosperms. As indicated by our study, the plasticity of signaling lipids in plants is remarkable. We would not be surprised to see *N*-acylethanolamines or *N*-acylglycerol derivatives from FAs different from the ones already identified (e.g. ALA, GLA, DHGLA). The phylogenetic distribution of lipids may be a consequence of interactions/adaptations to the surrounding conditions such as chemical plant-pollinator interactions, communication, defense mechanisms, waiting to be discovered.

## Methods

The material used can be found in the Supplementary Information.

### Synthesis of juniperoyl ethanolamide (JEA)

Under argon atmosphere, a solution of JuA (**5**) (2 mg, 0.007 mmol, 1 equiv) in 100 μL of anhydrous CH2Cl2 was cooled to 0 °C and treated with oxalyl chloride (2.3 μL, 0.03 mmol, 4 equiv) and 1.5 μL of DMF. The mixture was stirred for 4 h at room temperature in the dark to prevent the oxidation of **5**. Subsequently, the mixture was flushed with argon to remove the excess of oxalyl chloride. The corresponding acyl chloride was dissolved in 100 μL of anhydrous CH2Cl2, cooled with argon to 0 °C and an excess of ethanolamine (6.1 μL, 0.1 mmol, 14 equiv) was added. The mixture was left to react with stirring in the dark at room temperature for 12 h. The volatiles were removed with argon and the residue purified by HPLC (70% to 100% ACN over 40 min, 0.8 mL/min flow rate, detection at 200 nm). The desired compound JEA (**6**) was obtained as a colorless dense oil (2.1 mg, yield: 88%, scheme 1 Supplementary Information). The structure of JEA was confirmed by NMR spectroscopy and MS (Supplementary Information).

### Synthesis of 1/2-juniperoyl glycerol (1/2-JG)

Under argon atmosphere, a solution of JuA (**5**) (3 mg, 0.01 mmol, 1 equiv) dissolved in 100 μL of anhydrous CH2Cl2 was cooled to 0 °C and treated with oxalyl chloride (3.2 μL, 0.04 mmol, 4 equiv) and 1.5 μL of DMF. The mixture was stirred for 4 h at room temperature in the dark to prevent the oxidation of **5**. Subsequently, the mixture was flushed with argon to remove the excess of oxalyl chloride. The corresponding acyl chloride was dissolved in 200 μL of anhydrous CH2Cl2, and an excess of cis-1,3-O-benzylideneglycerol (9 mg, 0.05 mmol, 5 equiv) was added to the solution under argon. The reaction mixture was stirred at room temperature for 24 h in the dark and monitored by thin-layer chromatography (TLC) using *n*-hexane/ethyl acetate (4:1) as eluent. The reaction was then washed with NaOH (0.1 M) and with distilled water until a neutral pH. After evaporation, the intermediate product **7** (white oil) was obtained and used for the next step without any further purification. To a solution of **7** (4.7 mg, 0.01 mmol, 1 equiv) in 100 μL of anhydrous CH2Cl2, B-chlorocatecholborane (4.5 mg, 0.03 mmol, 3 equiv) was added. The reaction mixture was stirred for 2 h in the dark at room temperature and checked by TLC using *n*-hexane/ethyl acetate (1:1) as eluent. The product was observed by TLC (Rf = 0.2). The organic phase was washed with cold distilled water (100 μL) and CH3OH (100 μL) until neutrality. After the elimination of the solvent by flushing the solution with argon, the crude mixture was purified by HPLC (70% to 100% ACN over 40 min, 0.8 mL/min flow rate, detection at 200 nm). A mixture of the two isomers 1- and 2-juniperoyl glycerol (**8** and **9**) was obtained, as a colorless oil, in a ratio 2:1 (1-JG **8**: 2-JG **9**) (1.8 mg yield 48%, Scheme 2, Supplementary Information). The structures 1- and 2-JG were confirmed by NMR spectroscopy and MS (Supplementary Information).

### Plant selection, collection and preparation

Plant species were selected based on phylogeny^70^,^71^. At least two representatives from the green plant phylogenic clades were investigated including one Rhodophyta (red algae) and two lichens. Leaves were collected from the selected plants species, which were growing in different botanical gardens (Bern, Zurich, Graz, and Bonn), or from plants in the field. Several species samples were obtained from laboratory-grown plants (University of Neuchatel and University of Zurich) or purchased from specialized collections (Sammlung von Algenkulturen and www.plankton-shop.ch). In the case of several species, it was not possible to separate the leaves from the rest of the plant and, therefore, a sample of whole plant material was studied. A total of 71 plant species samples were investigated. Fresh or shade-dried plant material was extracted with DCM either by ultrasonication or homogenization/percolation, dried and stored at −20 °C until the analyses. 1–3 mg of DCM extract for each plant species were dissolved in 1 mL ACN + 0.1% formic acid that contained internal standards (IS) and were extracted over SPE using a previously published method^72^ and explained in detail in the Supplementary Information. The eluates were dried under nitrogen, reconstituted in ACN and analyzed by LC-MS/MS (method 1) (analyzed at least in triplicate). See Supplementary Information for details about the collection, identification, sample preparation, quantification and method performance.

For repeated analyses and the identification of JuA, JEA, 1/2-JG, AA, AEA, 1/2-AG, DHGLA, ScAc, ALA and GLA in the plant extracts, 33 plant species were investigated (Supplementary Table S1). These included all samples considered to be critical, together with those of other plants that either contained AA, AEA and 1/2-AG or not. DCM extracts (ca. 5 mg) were dissolved in ACN and purified by HPLC using a gradient of ACN and water. One aliquot containing all analytes of interest was collected, dried under nitrogen, reconstituted in ACN and split for analyses (Supplementary Information contains details about the sample preparation). These split samples were analyzed by LC-MS/MS (method 2) and GC-MS, respectively (Supplementary Information).

Additionally, the identities of the 1/2-AG, AEA and the other analytes run in positive mode, despite peak shifts, were confirmed by simultaneously recording the enhanced-product-ion (EPI) spectra of the most intense MRM transition (Q) in an information-dependent-acquisition (IDA) experiment (Supplementary Information). EPI scans for most analytes at low concentrations were not clear. However, at high concentrations, identification could be conducted without difficulties (data not shown).

### Quantification by LC-MS/MS

An API 4000 QTrap mass spectrometer equipped with a TurboIonSpray probe (AB Sciex) connected to a Shimadzu UFLC was used for the quantification (semi-quantitative approach) of the target compounds. Quantification of the target analytes (i.e. AA, 1/2-AG, AEA, LEA, PEA, OEA and SEA) was performed using our recently published method^42^,^72^. Additional analytes here quantified were MEA, quantified in the positive mode, and SA, OA, ALA, DHGLA and JA, quantified in the negative mode. Details about the chromatographic conditions and the evaluation of the method performance can be found in the Supplementary Information.

### Chromatographic conditions used for quantification by LC-MS/MS

Analytical LC separations were performed using a Reprosil-PUR C18 column as published^72^. Details about the mass spectrometric conditions are found in the Supplementary Information.

### Chromatographic conditions used for identification by LC-MS/MS

Analytical LC separations were performed using a Reprosil Saphir C18 column with a flow rate of 0.35 mL/min and oven temperature of 40 °C using a gradient of CH_3_OH with 2 mM ammonium acetate (eluent A) and water with 2 mM ammonium acetate and 0.1% formic acid (eluent B) for analyses in the positive mode of AEA, JEA, 1/2-AG and 1/2-JG. For the analyses of AA, JuA, AA ω-3, DHGLA, ScA, ALA and GLA, a gradient of ACN containing 0.1% formic acid (eluent B) and water containing 0.1% formic acid (eluent A) was used. Details are presented in the Supplementary Information.

### GC-MS analysis

The analyses of AEA, 1/2-AG, JEA, 1/2-JG, AA, JuA, AA ω-3, DHGLA, ScA, ALA and GLA were performed using a GC-MS from Agilent6890 N GC equipped with a 30 m HP-5MS column and a 5975 C EI-MS with a triple-axis detector as described^73^ with some modifications (Supplementary Information).

### Multivariate data analysis

Principal Component Analysis (PCA) was computed with the SIMCA© software (version 14, MKS Data Analytics Solutions, Umeå, Sweden). Autoscaling was performed as pre-processing.

## Additional Information

**How to cite this article**: Gachet, M. S. *et al*. Targeted metabolomics shows plasticity in the evolution of signaling lipids and uncovers old and new endocannabinoids in the plant kingdom. *Sci. Rep.* **7**, 41177; doi: 10.1038/srep41177 (2017).

**Publisher's note:** Springer Nature remains neutral with regard to jurisdictional claims in published maps and institutional affiliations.

## Supplementary Material

Supplementary Information

Supplementary Dataset 1

## Figures and Tables

**Figure:**