# Supplementary material for: Targeted metabolomics shows plasticity in the evolution of signaling lipids and uncovers old and new endocannabinoids in the plant kingdom
Source: Sci Rep. 2017 Jan 25;7:41177. doi: 10.1038/srep41177 (PMC5264637; doi:10.1038/srep41177)
Supplement: Supplementary Information [file srep41177-s1.pdf]

Targeted metabolomics shows plasticity in the evolution of signaling lipids and uncovers old and new endocannabinoids in the plant kingdom

María Salomé Gachet, Alexandra Schubert, Serafina Calarco, Julien Boccard, Jürg Gertsch

Content:

1. Materials and methods

1.1. Materials

1.2. Synthesis and identification of juniperoyl ethanolamide (JEA) and 1/2-juniperoyl glycerol (1/2-JG).

Scheme 1. Synthesis of JEA.

Scheme 2. Synthesis of 1-JG and 2-JG.

1.3. Information about the collection, identification and sample preparation of the plant species investigated.

Table S1. Information about the collection, identification and sample preparation of the plant species investigated.

1.4. Semi-quantitative analysis of targeted analytes using LC-MS/MS (validation).

Table S2. MRMs and chromatographic conditions for the analytes evaluated in plants.

Table S3. LODs, LOQs, dynamic range, linearity and accuracy of the MRM transitions selected for the quantification in plants.

Table S4. Results of the analysis of the quality control samples (QC), used to evaluate intra- and inter-day precision/stability and recovery.

1.5. Sample preparation, chromatographic conditions used for the analysis of plant samples (LC-MS/MS and GC-MS) and quantification of structural isomers.

Table S5. Ions used for selected ion monitoring in GC-MS.

2. Supplementary tables:

Supplementary Table S1: Concentrations of arachidonic acid or AA (20:4, $\Delta^{5,8,11,14}$, ω-6) and juniperonic acid or JuA (20:4, $\Delta^{5,11,14,17}$, ω-3) found in the 71 plant species analyzed.

Supplementary Table S2: Concentrations of 1/2-arachidonoyl glycerol or 1/2-AG and 1/2-juniperoyl glycerol or 1/2-JG found in the 71 plant species analyzed.

Supplementary Table S3: Concentrations of arachidonoyl ethanolamide or AEA and juniperoyl ethanolamide or JEA found in the 71 plant species analyzed.

Supplementary Table S4: Concentrations of myristoyl ethanolamide or MEA found in the 71 plant species analyzed.

Supplementary Table S5: Concentrations of linoleoyl ethanolamide or LEA found in the 71 plant species analyzed.

Supplementary Table S6: Concentrations of oleoyl ethanolamide or OEA found in the 71 plant species analyzed.

Supplementary Table S7: Concentrations of palmitoyl ethanolamide or PEA found in the 71 plant species analyzed.

Supplementary Table S8: Concentrations of stearoyl ethanolamide or SEA found in the 71 plant species analyzed.

Supplementary Table S9: Concentrations of stearic acid or STE (18:0) found in the 71 plant species analyzed.

Supplementary Table S10: Concentrations of oleic acid or OA (18:1, Δ^{9}) found in the 71 plant species analyzed.

Supplementary Table S11: Concentrations of jasmonic acid or JA found in the 71 plant species analyzed.

Supplementary Table S12: Concentrations of *α*-linolenic acid or ALA (18:3, $\Delta^{9,12,15}$, ω-3), *γ*-linolenic acid or GLA (18:3, $\Delta^{6,9,12}$, ω-6) and the unassigned ALA/GLA found in the 71 plant species analyzed.

Supplementary Table S13: Concentrations of dihomo-*γ*-linolenic acid or DHGLA (20:3, $\Delta^{8,11,14}$, ω-6), sciadonic acid or ScA (20:3, $\Delta^{5,11,14}$, ω-6) and the unassigned DHGLA/ScA found in the 71 plant species analyzed.

Supplementary Table S14: Concentrations of adrenic acid or AdA (22:4, $\Delta^{7,10,13,16}$, ω-6) found in the 71 plant species analyzed.

Supplementary Table S15: Concentrations of docosahexaenoic acid or DHA (22:6, $\Delta^{4,7,10,13,16,19}$, ω-3) found in the 71 plant species analyzed.

Supplementary Table S16: Concentrations (mean values in pmol/g plant weight) of the 14 analytes used for the Principal Component Analysis (PCA).

Supplementary Table S17: Contribution of the variables and eigenvalues obtained for the Principal Component Analysis (PCA) using Pearson (n-1).

3. Supplementary Figures:

Supplementary Fig. S1. Chemical structure of the analytes investigated.

Supplementary Fig. S2. Chromatograms (LC-MS/MS) showing the analysis of AA, 1/2-AG and AEA.

Supplementary Fig. S3. Chromatograms showing the analysis of C-20 PUFA metabolites.

Supplementary Fig. S4. GC-MS chromatograms showing the analysis of AA, JuA and arachidonic acid or AA ω-3 (20:4, $\Delta^{8,11,14,17}$, ω-3).

Supplementary Fig. S5. GC-MS chromatograms showing the analysis of 1/2-AG and 1/2-JG.

Supplementary Fig. S6. CG-MS chromatograms showing the analysis of AEA and JEA.

Supplementary Fig. S7. Chromatograms showing the analysis of ALA and GLA by LC-MS/MS and GC-MS.

Supplementary Fig. S8. Chromatograms showing the analysis of ScA and DHGLA by LC-MS/MS and GC-MS.

1. Materials and methods

1.1. Materials. Analytical and internal standards were purchased from Cayman Chemical, Tallinn, Estonia (arachidonic acid (AA or 5*Z*,8*Z*,11*Z*,14*Z*-eicosatetraenoic acid); AA-d_8 (5*Z*,8*Z*,11*Z*,14*Z*-eicosatetraenoic-5,6,8,9,11,12,14,15-d_8 acid); arachidonic acid ω3 (AA ω-3 or 8*Z*,11*Z*,14*Z*,17*Z*-eicosatetraenoic acid); anandamide (AEA or *N*-(2-hydroxyethyl)-5*Z*,8*Z*,11*Z*,14*Z*-eicosatetraenamide); AEA-d_4 (*N*-(2- hydroxyethyl-1,1,2,2-d_4)- 5*Z*,8*Z*,11*Z*,14*Z*-eicosatetraenamide); 2-arachidonoyl glycerol (2-AG or 5*Z*,8*Z*,11*Z*,14*Z*-eicosatetraenoic acid, 2-glyceryl ester); 2-AG-d_5 (5*Z*,8*Z*,11*Z*,14*Z*-eicosatetraenoic acid, 2-glyceryl-1,1,2,3,3-d_5 ester); 1-AG (5Z,8Z,11Z,14Z-eicosatetraenoic acid, 1-glyceryl ester); (±)-Jasmonic acid (JA or 3-oxo-2*R*-(2*Z*)2-penten-1*R*-yl-cyclopentaneacetic acid); linoleoyl ethanolamide (LEA or *N*-(2- hydroxyethyl)-9*Z,*12*Z*-octadecadienamide); LEA-d_4 (*N*-(2-hydroxyethyl-1,1,2,2-d_4)-9*Z*,12*Z*-octadecadienamide); myristoyl ethanolamide (MEA or *N*-(2-hydroxyethyl)-tetradecanamide); oleoyl ethanlonamide (OEA or *N*-(2-hydroxyethyl)-9*Z*-octadecenamide); OEA-d_4 (*N*-(2-hydroxyethyl-1,1,2,2-d_4)-9*Z*-octadecenamide); palmitoyl ethanolamide (PEA or *N*-(2-hydroxyethyl)-hexadecanamide); PEA-d_5 (*N*-(2-hydroxyethyl)-hexadecanamide-15,15,16,16,16-d_5); prostaglandin E_2 (PGE_2 or 9-oxo-11*α*,15S-dihydroxy-prosta-5Z,13E-dien-1-oic acid); PGE_2-d_4 (9-oxo-11*α*,15*S*-dihydroxy-prosta-5*Z*,13*E*-dien-1-oic-3,3,4,4-d_4 acid); stearoyl ethanolamide (SEA or *N*-(2-hydroxyethyl)-octadecanamide); stearic acid (STE or octadecanoic acid); oleic acid (OA or 9*Z*-octadecenoic acid); OA-d_{17} (9*Z*-octadecenoic-11,11,12,12,13,13,14,14,15,15,16,16,17,17,18,18,18-d_{17} acid); *α*-linolenic acid (ALA or 9*Z*,12*Z*,15*Z*-octadecatrienoic acid); ALA-d_{14} (9*Z*,12*Z*,15*Z*-octadecatrienoic-2,2,3,3,4,4,5,5,6,6,7,7,8,8-d_{14} acid); *γ*-linolenic acid (GLA or 6*Z*,9*Z*,12*Z*-octadecatrienoic acid); dihomo-*γ*-linolenic acid (DHGLA or 8*Z*, 11*Z*,14*Z*-eicosatrienoic acid); sciadonic acid (ScA or 5*Z*,11*Z*,14*Z*-eicosatrienoic acid); adrenic acid (AdA or 7*Z*,10*Z*,13*Z*,16*Z*-docosatetraenoic acid); docosahexaenoic acid (DHA or 4*Z*,7*Z*,10*Z*,13*Z*,16*Z*,19*Z*-docosahexaenoic acid)); or Lipidox Stockholm, Sweden (juniperonic acid (JuA or 5*Z*,11*Z*,14*Z*,17*Z*-eicosatetraenoic acid)). HPLC-grade methanol (CH_3OH), HPLC-grade acetonitrile (ACN), ammonium acetate, formic acid, pentafluorobenzylbromide, *N,N*-diisopropylethylamine, anhydrous dichloromethane (DCM), ethanolamine, dimethylformamide (DMF), cis-1,3-*O*-benzylideneglycerol, B-chlorocatecholborane and sodium hydroxide (NaOH) were obtained from Sigma-Aldrich, Steinheim, Germany. HPLC-grade ethyl acetate, DCM, *n*-hexane and 2-propanol were obtained from Fisher Scientific UK, Leicestershire, UK. HPLC-grade chloroform was purchased from Biosolve BV, Valkenswaard, the Netherlands. Oxalyl chloride was purchased from Acros Organics. Analytical TLC plates (silica gel $60F_{254}$) were obtained from Merck. Absolute ethanol (EtOH) was obtained from the Federal Swiss Government. Deionized water (18.2 MΩ x cm) was

obtained from an ELGA Purelab Ultra Genetic system (VWS (UK) Ltd, ELGA LabWater, UK). AQUASIL used for silanization was purchased from Thermo Scientific.

1.2. Synthesis and identification of juniperoyl ethanolamide (JEA) and 1/2-juniperoyl glycerol (1/2-JG).

^{1}H NMR spectra were recorded on a BRUKER AVANCE 300 spectrometer at 300 MHz and ^{13}C NMR spectra were recorded on a BRUKER AVANCE 400 spectrometer at 400 MHz, using $CDCl_3$ as the solvent and tetramethylsilane (TMS) as an internal standard.

The determination of the molecular weight of the synthesized molecules was performed on an API 4000 QTrap mass spectrometer equipped with a TurboIonSpray probe (AB Sciex) operated in LIT mode, performing enhanced product ion experiments (EPI). EPI were run in positive mode at 4000 Da/s as the scan rate, covering a mass range of 50-400 Da (*m/z*), collision energy (CE) of 30 V, collision energy spread (CES) of 15 V, using a LIT fill time of 200 msec enabling Q0 trapping; capillary ion spray voltage of +4500 V; curtain gas of 25 psi; GS1 of 20 psi; GS2 of 0 psi; at unit resolution Q1 and room temperature. The declustering potential (DP) was set to 55 V and 83 V for the analysis of JEA and 1/2-JG, respectively. Fresh solutions of JEA and 1/2-JG (1000 ng/mL and 3000 ng/mL, respectively, were dissolved in a 1:1 mixture of CH_3OH containing 2 mM ammonium acetate and water containing 2 mM ammonium acetate and 0.1% formic acid) and directly infused after equilibration (10 µL/min) for 60 cycles using MCA.

Juniperoyl ethanolamide (JEA) or *N*-(2- hydroxyethyl)-5*Z*,11*Z*,14*Z*,17*Z*-eicosatetraenamide (6) was synthesized following scheme 1 to yield a colorless oil (purity >96%). ^{1}H-NMR (300 MHz, $CDCl_3$) δ: 5.39-5.25 (m, 8H), 3.70 (t, 2H, *J*= 5.1 Hz), 3.40 (t, 2H, *J*= 5.1 Hz), 2.77 (t, 4H, *J*= 5.9 Hz), 2.18 (t, 2H, *J*= 7.7 Hz), 2.09-1.98 (m, 8H), 1.70 (t, 2H, *J*=7.5 Hz), 1.36-1.31 (m, 4H), 0.94 (t, 3H, *J*= 7.5 Hz) ppm. ^{13}C-NMR (400 MHz, $CDCl_3$) δ: 174.24, 131.99, 130.90, 130.14, 128.58, 128.33, 128.21, 127.83, 127.09, 63.09, 42.63, 36.06, 29.69, 29.28, 27.17, 27.14, 26.62, 25.63, 25.57, 25.54, 20.87, 14.33 ppm. ESIMS: $C_{22}H_{37}NO_2$ *m/z* (%) 348.03 (100) $[M+1]^+$.

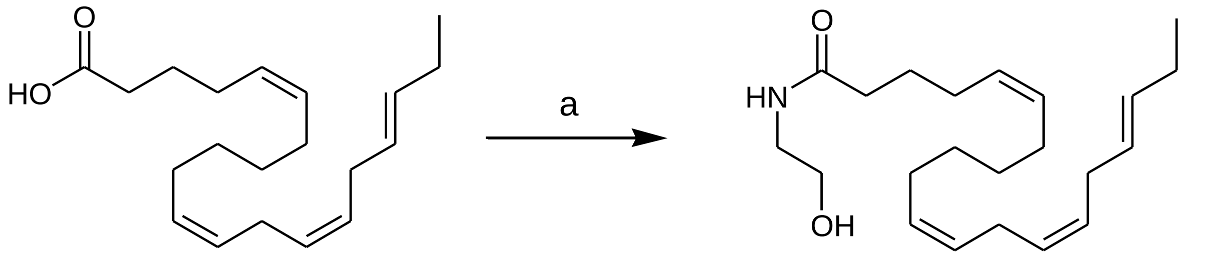

5 6

Scheme 1. Synthesis of JEA (6) from JuA (5). Reagents and conditions: (a) 1) oxalyl chloride, DMF, CH_2Cl_2, 0° to r.t., 4h; 2) ethanolamine, CH_2Cl_2 r.t., overnight.

1/2- juniperoy lglycerol (1/2-JG) or 5*Z*,11*Z*,14*Z*,17*Z*-eicosatetraenoic acid, 1/2-glyceryl ester (8 and 9) was synthesized following scheme 2 to yield a colorless oil (purity >96%).

^{1}H-NMR (300 MHz, $CDCl_3$) δ: 5.39-5.20 (m, 8H), 4.18-4.05 (m, 2H), 3.89-3.83 (m, 1H), 3.63 (dd, 1H, *J*= 3.9, 11.4 Hz), 3.53 (dd, 1H, *J*= 6.0, 11.4 Hz), 2.74 (t, 4H, *J*= 5.9 Hz), 2.29 (t, 2H, *J*= 7.7 Hz), 2.06-1.94 (m, 8H), 1.63 (t, 2H, *J*= 7.5 Hz), 1.32-1.28 (m, 4H), 0.91 (t, 3H, *J*= 7.5) ppm. ^{13}C-NMR (400 MHz, $CDCl_3$) δ: 174.14, 131.98, 131.08, 130.12, 128.32, 128.29, 128.21, 127.83, 127.09, 70.70, 65.17, 33.52, 29.69, 29.27, 27.14, 26.51, 25.63, 25.57, 25.54, 24.84, 20.60, 14.33 ppm. ESIMS: $C_{23}H_{38}O_4$ *m/z* (%) 379.32 (88) $[M+1]^+$.

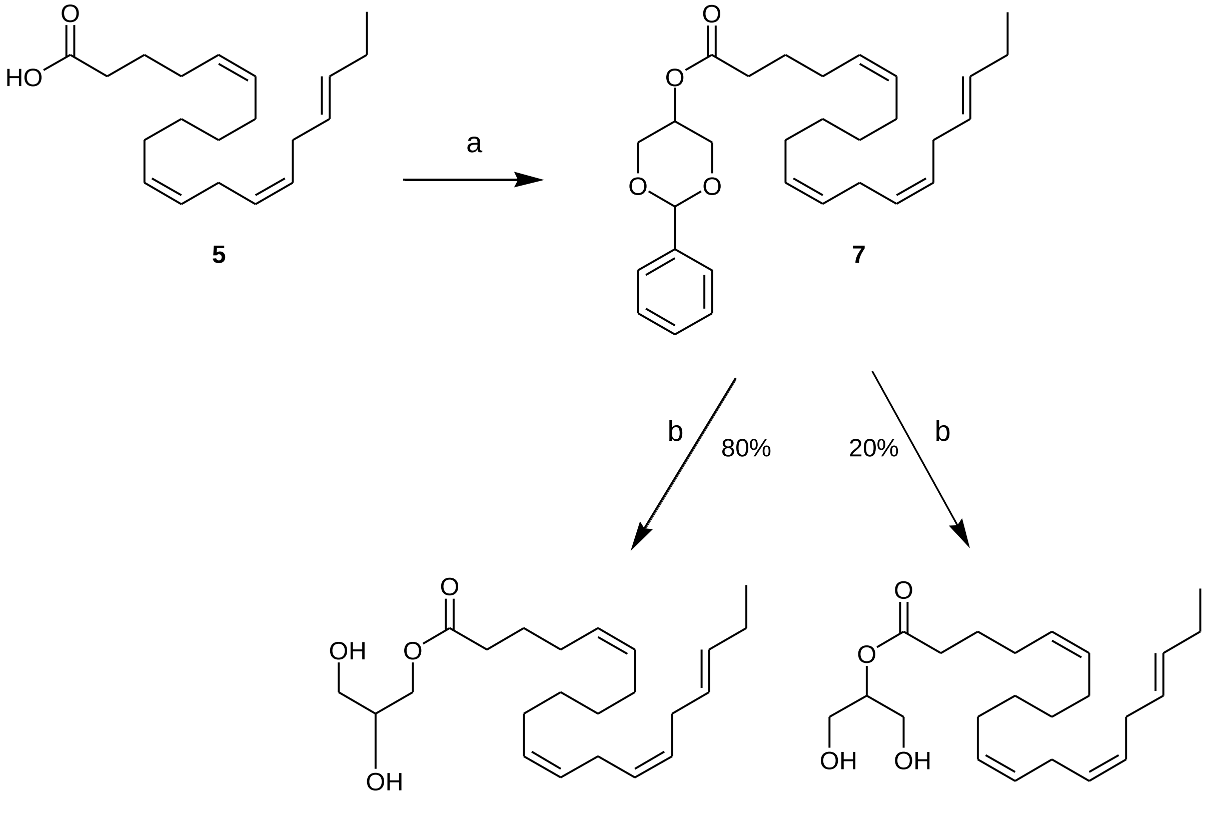

Scheme 2. Synthesis of 1-JG (8) and 2-JG (9) from JuA (5). Reagents and conditions: (a) 1) oxalyl chloride, DMF, CH_2Cl_2, 0° to r.t., 4h; 3) cis-1,3-*O*-Benzylideneglycerol, CH_2Cl_2, r.t., 24h, dark; (b) B-chlorocatecholborane, CH_2Cl_2, r.t., 2h, dark.

1.3. Information about the collection, identification and sample preparation of the plant species investigated. Samples from selected plant species (leaves (**L**) or whole plants (**WP**)) were identified and collected from the Botanical Gardens of Bern (**BeBG**) by Steffen Boch and Christopher Ball, at the Zurich Botanical Garden (**ZuBG**) by Michael Kessler and his group members and at the Graz Botanical Garden (**GrBG**) by Wolfgang Schühly. *Amborella trichopoda* (origin: DAVIS Arboretum of California), *Chloranthus spicatus* (origin: Dresden, Botanical Garden at the Technical University) and *Selaginella moellendorfii* (Felix Grewe) were kindly provided by Wolfram Lobin from the Botanical Garden in Bonn (**BoBG**). Ariel Bergamini and Maya Valentini, working at the Swiss Federal Research Institute WSL (**SFRI WSL**), helped us with the collection and identification of *Phaeoceros laevis* (collected at 680396/264425 in the Canton of Zurich close to Hochfelden). Edwin Urmi at Bosco-Gurin, Ticino (**Ti**) helped us with the collection and identification of *Hedwigia ciliate, Hylocomium splendens* and *Polytrichum juniperinum*. *Zea mays* was collected in the field (**CF**) in the Canton of Bern close to Köniz. A few fallen leaves from *Rhizophora mangle* were collected on Tortuga Bay-Santa Cruz Island, Galapagos, from a labeled mangrove. Cultured laboratory strains of *Anthoceros agrestis*, *A. punctatus* and *Chara vulgaris*, cultured at the Institute of Evolutionary Biology and Environmental Studies, University of Zurich (**UZu**), were kindly provided by Peter Szovenyi and Mariana Ricca; *Physcomitrella patens* cultured at the Institute of Biology, Laboratory of Cell and Molecular Biology, University of Neuchatel (**UNe**) was kindly provided by Didier Schaefer; and *Arabidopsis thaliana* growing at the Bern Botanical Garden was kindly provided by Marianne Suter Grotemeyer. *Klebsormidium elegans* was purchased from the Sammlung von Algenkulturen Göttingen (**SAG**) and *Caulerpa prolifera* and *Halymenia floresii* were ordered online (**OL**) from http://www.plankton-shop.ch/.

Plants were collected and processed at two different time points, following different procedures. During the first collection phase, ca. 20 g of fresh (**F**) plant material were extracted with ca. 200 mL of DCM while stirring at 3.4 rpm with a Polytron PT 3000 homogenizer (Kinematic AG, Littau, Switzerland) at room temperature. The extracts were filtered through glass wool and percolated with an additional 100 mL DCM. After removing the organic solvent, extracts were kept at -20°C until analysis (homogenization/percolation= **HP**). During the second collection phase, shade-dried (**D**), powdered plant materials (0.5-1 g) were extracted twice with 5 mL DCM by ultrasonication (each 5 min). The extracts were filtered through glass wool, dried and stored at -20°C prior the analysis (ultrasonication= **US**). Detailed information about each individual samples can be found in Table S1.

Table S1. Information about the collection, identification and sample preparation of the plant species investigated.

Group	#	Order	Family	Species	Collection	Plant part analyzed	Plant extracted	Extraction method	Sub-set reanalyzed
Angiosperms	1	Santalales	Viscaceae	*Viscum album* L.	BeBG	L	D	US	
	2	Brassicales	Brassicaceae	*Arabidopsis thaliana* (L.) Heynh.	BeBG	L	D	US	x
	3	Malvales	Malvaceae	*Theobroma cacao* L.	BeBG	L	D	US	x
	4	Rosales	Cannabaceae	*Cannabis sativa* L.	BeBG	L	D	US	x
	5	Malpighiales	Rhizophoraceae	*Rhizophora mangle* L.	CF	L	D	US	
	6	Malpighiales	Salicaceae	*Salix glabra* Scop.	BeBG	L	D	US	
	7	Poales	Poaceae	*Sorghum bicolor* (L.) Moench	GrBG	L	D	US	x
	8	Poales		*Hakonechloa macra* (Munro) Makino	BeBG	L	D	US	
	9	Poales		*Zea mays* L.	CF	L	D	US	
	10	Asparagales	Amaryllidaceae	*Allium sativum* L.	BeBG	L	D	US	
	11	Magnoliales	Magnoliaceae	*Liriodendron tulipifera* L.	BeBG	L	D	US	
	12	Chloranthales	Chloranthaceae	*Chloranthus spicatus* (Thunb.) Makino	BoBG	L	D	US	
	13	Austrobaileyales	Schisandraceae	*Schisandra chinensis* (Turcz.) Baill.	GrBG	L	D	US	
	14	Nymphaeales	Nymphaeaceae	*Nymphaea nouchali* Burm.f.	BeBG	L	D	US	
	15	Amborellales	Amborellaceae	*Amborella trichopoda* Baill.	BoBG	L	D	US	x
Gymnosperms	16	Gnetidae	Welwitschiaceae	*Welwitschia mirabilis* Hook.f.	BeBG	L	D	US	x
	17	Pinales	Pinaceae	*Pinus peuce* Griseb.	BeBG	L	D	US	x
	18	Pinales		*Pinus sylvestris* L.	BeBG	L	D	US	x
	19	Pinales		*Pinus mugo* Turra	BeBG	L	D	US	x
	20	Pinales		*Pinus cembra* L.	BeBG	L	D	US	x
	21	Pinales		*Larix gmelinii* var. *principis-rupprechtii* (Mayr) Pilg.	BeBG	L	D	US	x
	22	Pinales		*Picea abies* (L.) H.Karst.	BeBG	L	D	US	x
	23	Pinales		*Abies numidica* de Lannoy ex Carrière	BeBG	L	D	US	x
	24	Pinales		*Abies cephalonica* Loudon	BeBG	L	D	US	x
	25	Pinales		*Abies koreana* E.H.Wilson	BeBG	L	D	US	x
	26	Cupressales/Pinales	Taxaceae	*Taxus baccata* L.	BeBG	L	F	HP	x
	27	Cupressales	Cupressaceae	*Cryptomeria japonica* (Thunb. ex L.f.) D.Don	BeBG	L	D	US	x
	28	Cupressales		*Thujopsis dolabrata* (Thunb. ex L.f.) Sieb. & Zucc.	GrBG	L	D	US	x
	29	Araucariales	Araucariaceae	*Araucaria araucana* (Molina) K.Koch	BeBG	L	D	US	x
	30	Ginkgoales	Ginkgoaceae	*Ginkgo biloba* L.	BeBG	L	D	US	x
	31	Cycadales	Cycadaceae	*Cycas revoluta* Thunb.	BeBG	L	D	US	x
Monilophytes	*32*	Polypodiales	Polypodiaceae	*Polypodium vulgare* L.	BeBG	L	F/D	HP/US	
	33	Polypodiales	Davalliaceae	*Davallia canariensis* (L.) Sm.	ZuBG	L	F	HP	
	34	Polypodiales	Tectariaceae	*Tectaria zeylanica* (Houtt.) Sledge	ZuBG	L	F	HP	
	35	Polypodiales	Dryopteridaceae	*Polystichum aculeatum* (L.) Roth ex Mert.	BeBG	L	F	HP	
	36	Polypodiales	Onocleaceae	*Onoclea sensibilis* L.	ZuBG	L	F	HP	
	37	Polypodiales	Blechnaceae	*Blechnum spicant* (L.) Sm.	ZuBG	L	F	HP	
	38	Polypodiales	Thelypteridaceae	*Thelypteris palustri* Schott	ZuBG	L	F	HP	
	39	Polypodiales	Cystopteridaceae	*Gymnocarpium robertianum* (Hoffm.) Newman	BeBG	L	F	HP	
	40	Polypodiales		*Asplenium trichomanes* L.	BeBG	L	F	HP	
	41	Polypodiales	Aspleniaceae	*Phyllitis scolopendrium* (L.) Newman	BeBG	L	F	HP	
	42	Polypodiales	Pteridaceae	*Adiantum venustum* D. Don	ZuBG	L	F	HP	
	43	Cyatheales	Cyatheaceae	*Sphaeropteris cooperi* (F.Muell.) R.M.Tryon	BeBG	L	F	HP	
	44	Salviniales		*Salvinia natans* (L.) All.	ZuBG	L	F	HP	
	45	Salviniales	Salviniaceae	*Salvinia molesta* D. S. Mitch.	BeBG	L	D	US	x
	46	Schizaeales	Anemiaceae	*Anemia phyllitidis* (L.) Sw.	ZuBG	L	F	HP	
	47	Schizaeales	Lygodiaceae	*Lygodium volubile* Sw.	ZuBG	L	F	HP	
	48	Osmundales	Osmundaceae	*Osmunda regalis* L.	ZuBG	L	F	HP	
	49	Marattiales	Marattiaceae	*Angiopteris evecta* (G.Forst.) Hoffm.	ZuBG	L	F	HP	
	50	Equisetales	Equisetaceae	*Equisetum trachyodon* (A. Braun) W.D.J. Koch	ZuBG	L	F	HP	x
	51	Psilotales	Psilotaceae	*Psilotum nudum* (L.) P. Beauv.	ZuBG	L	F	HP	x
Lycophytes	52	Selaginellales	Selaginellaceae	*Selaginella moellendorffii* Hieron.	BoBG	L	D	US	x
	53	Selaginellales		*Selaginella pallescens* (C.Presl) Spring	BeBG	L	F/D	HP/US	x
	54	Lycopodiales	Lycopodiaceae	*Huperzia phlegmaria* (L.) Rothm.	ZuBG	L	F	HP	x
Hornworts	55	Anthocerotales	Anthocerotaceae	*Anthoceros agrestis* Paton nom. cons. prop.	UZu	WP	D	US	x
	56	Anthocerotales		*Anthoceros punctatus* L.	UZu	WP	D	US	
	57	Notothyladales	Notothyladaceae	*Phaeoceros laevis* (L.) Prosk.	SFRI WSL	WP	D	US	
Mosses	58	Funariales	Funariaceae	*Physcomitrella patens* (Hedw.) Bruch & Schimp.	UNe	WP	D	US	x
	59	Funariales		*Funaria hygrometrica* Hedw.	BeBG	WP	D	US	
	60	Polytrichales	Polytrichaceae	*Polytrichum juniperinum* Hedw.	Ti	WP	D	HP	
	61	Hedwigiales	Hedwigiaceae	*Hedwigia ciliata* (Hedw.) P.Beauv.	Ti	WP	D	HP	
	62	Hypnales	Hylocomiaceae	*Hylocomium splendens* (Hedw.) Schimp. in B.S.G.	Ti	WP	D	HP	x
Liverworts	*63*	Marchantiales	Conocephalaceae	*Conocephalum conicum* (Linnaeus) Lindb.	BeBG	WP	D	US	x
	64	Marchantiales	Marchantiaceae	*Marchantia polymorpha* L.	BeBG	WP	D	US	
	65	Marchantiales	Ricciaceae	*Riccia fluitans* L.	BeBG	WP	D	US	
Algaes	*66*	Charophytes	Characeae	*Chara vulgaris* Linnaeus	UZu	WP	D	US	x
	67	Charophytes	Klebsormidiaceae	*Klebsormidium elegans* Lokhorst	SAG	WP	D	US	
	68	Chlorophyta	Caulerpaceae	*Caulerpa prolifera* (P. Forsskal) Lamouroux	OL	L	D	US	
	69	Rhodophyta	Halymeniaceae	*Halymenia floresii* (Clemente y Rubio) C. Agardh	OL	L	D	US	
Lichens	70	Lecanorales	Parmeliaceae	*Cetraria islandica* (L.) Ach.	BeBG	WP	D	US	
	71	Teloschistales	Teloschistaceae	*Xanthoria parietina* (L.) Th. Fr.	BeBG	WP	D	US	x

BeBG= Bern Botanical Garden ; **ZuBG**= Zurich Botanical Garden ; **GrBG**= Graz Botanical Garden; **BoBG**= Bonn Botanical Garden; **UZu**= University of Zurich; SFRI WSL= Swiss Federal Research Institute WSL; **UNe**= University of Neuchatel; **Ti**= Bosco-Gurin (Ticino); **SAG**= Sammlung von Algenkulturen; **CF**= collected on the field; OL= ordered online; **L**= leaves; **WP**= whole plant; **US**= ultrasonication; **HP**= homogenization/percolation; **D**= dried plant material; **F**= fresh plant material.

1.4. Semi-quantitative analysis of targeted analytes using LC-MS/MS (validation)

Mass spectrometric conditions. Analyses were performed using an API 4000 QTrap mass spectrometer equipped with a TurboIonSpray probe (AB Sciex) connected to a Shimadzu UFLC. Data were acquired and processed using Analyst software version 1.5 (AB Sciex Concord, Ontario, Canada). The analysis was performed in the negative ionization mode for AA, AdA, ALA, DHA, DHGLA, JA, PGE_2, OA and STE and in the positive mode for AEA, 1/2-AG (both isomers were measured), LEA, MEA, OEA, PEA and SEA. The chemical structures of the investigated analytes are shown in supplementary Fig. S1. The source used nitrogen as a curtain gas and was operated at a capillary ion spray voltage of -4250 V in the negative, and +4500 V in the positive modes, respectively; temperature of 600°C; curtain gas of 25 psi; GS1 of 50 psi; GS2 of 50 psi; entrance potential of 10 V and collision cell exit potentials of 10 V. Analyses were performed in the multiple reaction monitoring mode (MRM) using one quantifier ion (Q) and at least one qualifier ion (q). The schedule MRM function was applied in both scan modes (target scan time of 1 sec and MRM detection window of 60 sec). MRM transitions are presented in the Table S2.

The information-dependent-acquisition (IDA) experiments applied the following criteria: select 1 to 2 of the most intense peaks which exceeded 500 counts per second (cps) to enable dynamic background subtraction, never exclude former target ions and set a mass tolerance of 250 mDa. Two identical enhanced product ion scans (EPI) were recorded over a mass range of *m/z* 50-500 at a rate of 4000 amu/s using the linear ion trap with a fixed fill time of 50 ms, which enabled Q_0 trapping and Q_1 at unit resolutions. The CE was set to 30 eV with a CES of 15 eV and the DP was set to 30 eV. The MRM used for these experiments included only the most intense ions (Q). The IDA experiments were only performed in the positive mode.

Quantification by LC-MS/MS. Quantification (semi-quantitative) was performed using external calibration standards, following our recently published method with a few modifications.[1,2] Standard mixtures used as calibrators (calibration solutions) and internal standards (IS) were prepared in EtOH at concentrations of 0.01 mg/mL and later diluted accordingly. The concentrations of the labeled IS used for the analysis were: 20 ng/mL for AEA-d_4, LEA-d_4, OEA-d_4, PEA-d_5 and PGE_2-d_4; 50 ng/mL for 2-AG-d_5; 1000 ng/mL for ALA-d_{14}; 1500 ng/mL for AA-d_8; and 6500 ng/mL for OA-d_{17}. The concentrations of the calibrators are listed under dynamic range (low and high) in Table S3. The reported concentrations were obtained upon spiking 10 µL of a tenfold concentrated solution brought up to a final volume of 100 µL. Calibration curves (5-11 calibration points, run in triplicate) and quality control samples (CQ_0: non-spiked samples and CQ_7: samples spiked with an intermediate concentration (Table S4)) were prepared for the *Arabidopsis thaliana* (***At***) and *Physcomitrella*

patens (***Pp***) DCM extracts and evaluated both prior and during the analysis of these samples. Method performance (LOD, LOQ, linearity, accuracy, precision, stability and recoveries) was evaluated using both matrixes and, based on the results, the best matrix was selected for the quantification of each individual analyte in the samples. Table S3 shows results only for the matrix selected for quantification, while Table S4 shows the results obtained in both matrixes. The range of concentrations for the analytes found in the samples was very broad and, thus, two calibration curves (low and high) were used for the quantification of most analytes (Table S3). Overall, the method validation parameters showed good performance, which is transparently presented in Tables S3 and S4. Some analytes, which used as IS a label standard different than their own, (e.g., JA, MEA, OA STE, SEA) showed higher variability (CV%), lower accuracy and a broader range of recoveries. Recoveries were between 70-130% (exemplified for one concentration in Table S4, but were similar at other concentrations within the dynamic ranges evaluated) with the exception of ALA, DHGLA, JA and STE, for which recoveries were rather high, and MEA, for which recoveries were rather low (Table S4). We are aware of the complexity of the matrices presented by each individual plant species and the potential effects (suppression/ enhancement) that each single matrix can have in the quantification (as exemplified for *At* and *Pt* in Tables S3 and S4). Therefore, the values generated and reported here are treated as semi-quantitative.

The identification of each individual analyte in the plant extracts was based on the retention time of the analytes and the IS. Only if the peak fitted the expected retention time of both, it was considered for quantification. An exception was made for some samples (e.g., gymnosperms) in the analysis of AEA and 1/2-AG, within which some peaks were slightly shifted. Later, these peaks were identified as JEA and 1/2-JG (see below), but were still quantified as AEA and 1/2-AG. Samples were analyzed at least in triplicate together with blank-blank samples (containing neither matrix nor IS), blank samples (only IS), calibrators and QC samples. A flushing solution (water/CH_3OH/ACN/2-propanol (1:1:1:1)) and ACN were run over the column between each sample injection to avoid carryovers. The quantification was critical at low concentrations (LOQ). Samples displaying peaks at the LOQ or around it were evaluated carefully (i.e., criteria: signal-to-noise ratio above 10, no interferences with the blank and consistency between the repetitions measured). In order to obtain the concentration found in plants, the analytical amount (ng/mL) was multiplied by 0.1 (to correct the volume of 1 mL to the final volume of 0.1 mL) and then normalized, first, to the amount of DCM extract analyzed and, later, to the amount of plant material (either fresh or dried) that had been used to generate the DCM extract.

Table S2. MRMs and chromatographic conditions for the analytes evaluated in plants.

Analyte	IS used	Retention time (min)	Mode	Q/q	Precursor ion (*m/z*)	Product ion (*m/z*)	Declustering potential (eV)	Collition energy (eV)	Observation
Analytes measured in negative mode									
AA	AA-d_8	8.7	neg	Q	303	205	-80	-18	
				q	303	59	-80	-37	
				q	303	259	-80	-19	
AdA	AA-d_8	9.1	neg	Q	331	59	-95	-42	
				q	331	287	-95	-21	
				q	331	313	-95	-25	
ALA	ALA-d_{14}	8.4	neg	Q	277.4	127	-80	-37	
				q	277.4	59.2	-80	-35	
				q	277.4	259.2	-80	-24	
DHA	AA-d_8	8.6	neg	Q	327	191	-35	-15	
				q	327	133	-35	-15	
				q	327	59	-75	-15	
DHGLA	ALA-d_{14}	8.9	neg	Q	305.4	287.1	-80	-23	
				q	305.4	113	-80	-36	
				q	305.4	232.8	-80	-25	
JA	PGE_2-d_4	4.9	neg	Q	209.3	58.8	-58	-25	
				q	209.3	80.8	-58	-25	
				q	209.3	108.8	-58	-25	
PGE_2	PGE_2-d_4	5.4	neg	Q	351	315	-70	-25	
				q	351	271	-70	-16	
OA	OA-d_{17}	9.0	neg	Q	281.5	97	-85	-35	
				q	281.5	59	-85	-45	
				q	281.5	70.8	-85	-45	
STE	OA-d_{17}	9.4	neg	Q	283.5	264.7	-90	-27	
				q	283.5	59	-90	-50	
Analytes measured in positive mode									
AEA	AEA-d_4	8.1	pos	Q	348	62	56	42	
				q	348	133	56	33	
2AG*	2-AG-d_5*	8.2/8.3	pos	Q	379	203	82	25	double peak
				q	379	287	51	18	
LEA	LEA-d_4	8.1	pos	Q	324	62	74	36	
				q	324	109	74	32	
MEA	PEA-d_5	7.6	pos	Q	272.4	255	60	20	
				q	272.4	62	60	30	
OEA	OEA-d_4	8.5	pos	Q	326	62	72	36	
				q	326	309	72	21	
PEA	PEA-d_5	8.3	pos	Q	300	62	78	36	
				q	300	283	78	19	
SEA	PEA-d_5	8.9	pos	Q	328	62	72	31	
				q	328	311	72	22	
IS measured in negative mode									
AA-d_8		8.6	neg	IS	311	59	-90	-38	
ALA-d_{14}		8.3	neg	IS	291.5	271.3	-82	-25	
OA-d_{17}		9.0	neg	IS	298.6	280.4	-90	-30	
PGE_2-d_4		5.4	neg	IS	355	319	-64	-17	
IS measured in positive mode									
AEA-d_4		8.1	pos	IS	352	66	60	35	
2-AG-d_5		8.2/8.3	pos	IS	384	287	62	17	double peak
LEA-d_4		8.1	pos	IS	328	66	70	35	
OEA-d_4		8.5	pos	IS	330	66	72	32	
PEA-d_5		8.3	pos	IS	305	62	70	40	

* Both isomers (i.e. 1- and 2-AG) were measured
nd: not determine
Q: MRM transition used as quantifier
q: MRM transition used as quanlifier
IS: internal standard

Table S3. LODs, LOQs, dynamic range, linearity and accuracy of the MRM transitions selected for the quantification in plants using either *P. patens* (*Pp*) or *A. thaliana* (*At*).

Analyte	Precursor ion (*m/z*)	Product ion (*m/z*)	Matrix used	LOD (ng/mL)	LOQ (ng/mL)	Dynamic range low (ng/mL)	R^2±SD (n=3)	Slope	CV slope (%)	Intercept	Amount spiked (ng/mL)	Accuracy (%, n= 6)
AA	303	205	*At*	41	104	41-25000	0.9963±0.0042	0.0009±0.0002	19	-0.12378±0.2765	4000	107±9
AdA	331	59	*At*	64	160	64-2500	0.9989±0.0008	0.0010±0.0002	16	-0.0007±0.0286	400	85±7
AEA	348	62	*At*	0.5	1.3	0.5-50*	0.9992±0.0010	0.0293±0.0016	5	0.0003±0.0013	8	98±10
2AG	379	203	*At*	4.1	10.2	4.1-2500	0.9977±0.0027	0.0087±0.0008	10	0.1190±0.6224	400	99±6
ALA	277.4	127	*Pp*	260	1600	260-25000	0.9972±0.0013	0.0007±0.0001	10	0.0750±0.4197	4000	143±9
DHA	327	191	*At*	640	640	640-25000	0.9970±0.0017	2.35E-05±1.8E-6	8	-0.0007±0.0075	4000	107±14
DHGLA	305.5	287.1	*Pp*	102	640	102-25000	0.9965±0.0029	0.0034±0.0005	21	0.2622±0.2963	4000	110±10
JA	209.3	58.8	*At*	0.3	5	0.3-500*	0.9972±0.0008	0.329±0.0152	46	0.1501±0.1743	80	98±10
LEA	324	62	*Pp*	0.03	0.2	0.03-50	0.9907±0.0070	0.0123±0.015	12	0.0129±0.0300	8	106±20
MEA	272.4	255	*At*	0.4	0.4	0.4-250*	0.9907±0.0089	0.0331±0.0134	41	0.1346±0.1640	40	71±29
OA	281.5	97	*Pp*	3200	3200	3200-50000	0.9874±0.0136	0.0001±0.00001	5	0.120±0.0437	8000	100±16
OEA	326	62	*At*	0.2	0.4	0.2-250*	0.9990±0.0007	0.0636±0.0005	1	-0.0001±0.0683	40	103±9
PEA	300	62	*At*	0.2	6.4	0.2-250	0.9981±0.0011	0.0385±0.058	15	-0.0013±0.0880	40	87±12
PGE_2	351	315	*At*	0.4	1.0	0.4-250	0.9979±0.0030	0.0497±0.0011	2	-0.371±0.0817	40	98±5
SEA	328	62	*At*	0.2	2.6	0.2-250*	0.9969±0.0022	0.0568±0.0189	33	-0.631±0.0639	40	109±40
STE	283.5	264.7	*At*	3200	8000	3200-50000	0.9813±0.0159	0.0005±0.0001	13	0.4767±0.7443	8000	95±12

Analyte	Precursor ion (*m/z*)	Product ion (*m/z*)	Matrix used	Dynamic range high (ng/mL)	R^2±SD (n=3)	Slope	CV slope (%)	Intercept	Amount spiked (ng/mL)	Accuracy (%, n= 6)
AA	303	205	*At*	41-375000	0.9997±0.0001	0.0007±0.0001	21	0.3022±0.5134	250000	125±37
AdA	331	59	*At*	-	-	-	-	-	-	-
AEA	348	62	*At*	0.5-500	0.9999±0.0000	0.0295±0.0029	10	0.0096±0.0247	50	111±3
2AG	379	203	*At*	4.1-37500	0.9842±0.0241	0.0071±0.0005	7	2.5503±2.7310	25000	125±24
ALA	277.4	127	*Pp*	260-375000	0.9954±0.0005	0.0004±0.00001	3	1.9085±0.7301	250000	97±11
DHA	327	191	*At*	-	-	-	-	-	-	-
DHGLA	305.5	287.1	*Pp*	-	-	-	-	-	-	-
JA	209.3	58.8	*At*	0.3-500	0.9937±0.0043	0.0527±0.0073	14	0.2617±0.2907	200	123±24
LEA	324	62	*Pp*	0.03-750	0.9973±0.0038	0.0106±0.0023	22	0.0595±0.0611	500	108±25
MEA	272.4	255	*At*	-	-	-	-	-	-	-
OA	281.5	97	*Pp*	3200-500000	0.9984±0.0014	0.0001±0.00001	8	0.4498±0.3490	50000	105±30
OEA	326	62	*At*	0.2-250	0.9990±0.0009	0.0656±0.0024	4	0.0582±0.0250	100	95±9
PEA	300	62	*At*	0.2-2500	0.9999±0.0002	0.0501±0.0082	16	-0.1034±0.2417	250	109±16
PGE_2	351	315	*At*	-	-	-	-	-	-	-
SEA	328	62	*At*	0.2-3750	0.9870±0.0100	0.0340±0.0061	18	1.0206±1.2350	2500	132±23
STE	283.5	264.7	*At*	3200-500000	0.9760±0.0300	0.0002±0.0001	40	3.1604±0.5259	50000	148±34

*For these analytes, a narrower concentration range was used in the analysis of samples found at low levels (i.e. AEA at 1.3-20; JA at 5-32; MEA at 0.4-16; OEA at 0.41-40; and SEA at 2.6-100 ng/mL, respectively)

Table S4. Results of the analysis of the quality control samples (QC), used to evaluate intra- and inter-day precision/stability and recovery using *P. patens* (*Pp*) and *A. thaliana* (*At*)

			**Physcomitrella patens**							**Arabidopsis thaliana**			
			**QC_0**		**QC_7**					**QC_0**	**QC_7**		
			**Intra-day (n=6)**	**Inter-day (n=6)**		**Intra-day (n=6)**		**Inter-day (n=3)**		**Intra-day (n=6) CQ_0**		**Intra-day (n=6) CQ_7**	
**Analyte**	**Precursor ion (*m/z*)**	**Product ion (*m/z*)**	**CV (%)**	**CV (%)**	**Amount spiked (ng/mL)**	**Recovery (%)**	**CV (%)**	**Recovery (%)**	**CV (%)**	**CV (%)**	**Amount spiked (ng/mL)**	**Recovery (%)**	**CV (%)**
AA	303	59	6.1	16.5	4000	np	7.1	np	8.2	3.7	4000	105+8	7.4
AdA	331	59	10.0	12.8	400	75±16	15.5	82±11	9.8	<lod	400	85+7	8.3
AEA	348	62	11.5	6.1	8	115±35	10.1	104±9	4.0	<lod	8	98+10	10.0
2AG	379	203	8.9	17.4	400	np	10.3	np	11.6	<lod	400	99+6	6.0
ALA	277.4	127	3.2	8.1	4000	143±9	1.5	103±50	14.8	4.1	4000	61±33	3.9
DHA	327	191	<lod	<lod	4000	107±14	13.1	102±13	12.5	<lod	4000	94+8	8.4
DHGLA	305.5	287.1	7.1	12.5	4000	116±15	7.0	137±26	5.7	<lod	4000	110+10	8.8
JA	209.3	58.8	<lod	<lod	80	94±6	55.7	96±45	37.7	9.9	80	98+10	5.5
LEA	324	62	13.1	16.5	8	106±20	8.9	98±21	11.6	8.7	8	np	7.0
MEA	272.4	255	<lod	<lod	40	135±51	64.8	156±118	68.2	<lod	40	71+29	41.0
OA	281.5	97	26.5	29.1	8000	100±16	14.2	82±15	20.1	23.4	8000	105+5	4.7
OEA	326	62	10.1	7.6	40	99±4	4.1	96±17	16.6	28.0	40	103+9	9.0
PEA	300	62	12.0	23.4	40	108±7	5.6	107±9	4.3	14.2	40	87+12	9.0
PGE_2	351	315	<lod	<lod	40	104±6	5.6	98±9	8.7	<lod	40	98+5	4.6
SEA	328	62	28.5	23.3	40	63±36	53.4	83±6	16.1	11.7	40	109+40	33.0
STE	283.5	264.7	14.5	25.1	8000	43±22	21.6	61±15	16.6	12.5	8000	95+12	8.1

np= not possible to determine due to the high endogenous concentration present in the matrix.

1.5. Sample preparation, chromatographic conditions used for the analysis of plant samples (LC-MS/MS and GC-MS) and quantification of structural isomers.

DCM extracts (1-3 mg) were weighed into 2 mL Eppendorf tube and dissolved in 1 mL ACN with 0.1 % formic acid and 10 µL of IS solution. The mixture was thoroughly vortexed and placed inside the refrigerator (6°C) for 10 min to facilitate the extraction process. Subsequently, the sample was centrifuged at 16,100 g at 4°C for 5 min. The supernatant was transferred to a silanized glass tube, diluted with 9 mL water and extracted by solid-phase extraction (SPE) (C18 Sep-Pak cartridge from Waters AG Baden-Dättwil, Switzerland, pre-activated with 3 mL CH_3OH and equilibrated with 3 mL 10% ACN). Cartridges were washed with 3 mL 10% ACN and eluted with 3 mL ACN/ethyl acetate (1:1). The eluates were evaporated to dryness under nitrogen. The samples were reconstituted in 100 µL ACN (final volume) and centrifuged for 5 min at 16,100 g at 4°C. 80 µL of these samples were carefully pipetted out, placed into conic amber vials and analyzed by LC-MS/MS (10 µL injection volume) [1].

Samples preparation for identification. DCM extracts of 33 selected plant species (ca. 5 mg) were dissolved in ACN and purified by HPLC (Agilent/HP 1100 series HPLC system) using a Lichrospher 100 RP-18 column at a flow rate of 0.8 mL/min and a gradient of ACN and water (70% to 100% ACN over 40 min, maintained at 100% ACN for 10 min, detection at 200 nm). One aliquot (eluting at 6 to 22 min, which contained all analytes of interest) was collected into silanized glass tubes. Samples were dried under nitrogen, reconstituted in ACN, split in two and analyzed by both LC-MS/MS (method 2) and GC-MS.

Chromatographic conditions used for the quantifications (LC-MS/MS method 1). Analytical LC separations used for the quantification were performed on a Reprosil-PUR C18 column (3 µm particle size; 2×50 mm, Dr. A. Maisch, High Performance LC-GMBH, Ammerbuch, Germany) with a flow rate of 0.35 mL/min and oven temperature of 40°C using a gradient of CH_3OH containing 2 mM ammonium acetate (eluent B) and water containing 2 mM ammonium acetate and 0.1% formic acid (eluent A). The gradient was as follows: 15% eluent B for 0.5 min; 15-70% B from 0.5 - 3.5 min; 70-99% B from 3.5-8.0 min, held at 99% from 8.0-11.0. From 11.0-11.5 min, the column was re-equilibrated to 15% B and conditioned from 11.5-13 min at 15% B. The autosampler was cooled at 4°C [1].

Chromatographic conditions used for the identification of structural isomers (LC-MS/MS method 2). Analytical LC separations used for the identification were performed using a ReproSil Saphir 100 C18 column (3 µm particle size; 2×50 mm, Dr. A. Maisch, High Performance LC-GMBH, Ammerbuch, Germany) with a flow rate of 0.35 mL/min and oven

temperature of 40°C. The autosampler was cooled at 4°C. These conditions were used for the analysis in positive and negative modes, employing different solvent compositions and gradients.

The analysis in the positive mode of AEA, JEA, 1/2-AG and 1/2-JG was performed using a gradient of CH_3OH containing 2 mM ammonium acetate (eluent B) and water containing 2 mM ammonium acetate and 0.1% formic acid (eluent A). The gradient was as follows: 65-80% eluent B from 0.0-20.0 min; 80-99% B from 20.0-21.0 min, held at 99% from 21.0-25.0. From 25.0-26.0 min, the column was re-equilibrated to 15% B and conditioned from 26.0-30.0 min at 65% B.

The analysis in negative mode of AA, JuA, AA ω-3, ALA, GLA, DHGLA and ScAc was performed using a gradient of ACN containing 0.1% formic acid (eluent B) in water containing 0.1% formic acid (eluent A). The gradient was as follows: 50-70% eluent B from 0.0-30.0 min; 70-99% B from 30.0-31.0 min, held at 99% from 31.0-34.0 min. From 34.0-35.0 min, the column was re-equilibrated to 50% B and conditioned from 35.0-40.0 min at 50% B.

As expected, the tuning of JEA, 1/2-JG, JuA, AA ω-3, GLA and ScAc yielded almost the same MRM transitions and very similar collision energies (CE) and declustering potentials (DP) as their corresponding structural isomers (i.e., AEA, 1/2-AG, AA, ALA and DHGLA, reported in Table S2). Therefore, the MRM transitions reported in Table S2 were selected for the analysis of the plant samples. In the case of ALA and GLA, MRM transition 227/205 (DP: -90, CE: -25) was used instead of 277/127; and in the case of DHGLA and ScA, MRM transitions 305/59 (DP: -90, CE: -35) and 305/261 (DP: -90, CE: -25) were used instead of 305/113 and 305/233. The chemical structures of the investigated structural isomers of AA, ScA, ALA, AEA and 1/2-AG are shown in supplementary Fig. S1.

GC-MS analysis. The analyses of AEA, 1/2-AG, JEA, 1/2-JG, AA, JuA, AA ω-3, DHGLA, ScA, ALA and GLA were performed after derivatization according to [3]. In brief, the carboxylic groups of the fatty acids were esterified with pentafluorobenzylbromide and *N,N*-diisopropylethylamine (i.e., 20 µL of a 1g in 3mL ACN solution of pentafluorobenzylbromide and 20 µL *N,N*-diisopropylethylamine were added to the samples dissolved in 50 µL ACN for their derivatization at 45°C for 25 min). After drying the samples under nitrogen, the hydroxyl groups of the endocannabinoids were derivatized by adding 50 µL of dimethylisopropylsilyl imidazole (23°C for 60 min). The excess of derivatization reagent was removed by running the samples over a Sephadex LH-20 column (300 mg), eluting with *n*-hexane/chloroform/CH_3OH (10:10:1). After drying under nitrogen, samples were dissolved in 25 µL of *n*-hexane and analyzed with a GC-MS from Agilent6890 N GC equipped with a 30 m HP-5MS column and a 5975 C EI-MS with a triple-axis detector. Helium was used as carrier gas at a flow rate of 1.5 mL/min. The inlet temperature was kept at 250°C and 1 µL of the

sample was injected in splitless mode. The oven temperature program was as follows: initial temperature of 150°C (hold time 1 min), increasing by 8°C/min to 280°C (hold time 20 min). The MS conditions were: ionization energy 70eV, ion source temperature 230°C, MS Quant temperature 150°C and Aux temperature 280°C. Specific ions were used for selected ion monitoring (SIM), see Table S5. Samples were analyzed twice, first measuring endocannabinoids and, subsequently, the fatty acids using the same chromatographic conditions, but different SIM settings.

Table S5. Ions used for selected ion monitoring in GC-MS.

**Analytes and IS**	**Quantlifier Ion (*m/z*)**	**Qualifier Ions (*m/z*)**	**Retention time (min)**
AEA	404	432	19.41
JEA	404	432	19.80
AEA-d_4	408		19.38
2AG	535	479	21.63
2JG	535	479	22.13
2AG-d_5	540		21.59
1AG	535	479	22.16
1JG	535	479	22.76
1AG-d_5	540		22.13
AA	484	386	16.41
AA ω-3	484	415	16.75
JuA	484	455/441	16.69
AA-d_8	392	350/402	16.37
DHGLA	486	305	16.68
ScAc	486	415/401	16.58
ALA	458	360	14.81
GLA	458	429/402	15.11

Quantification of ALA and GLA. The determination of the concentrations of ALA and GLA was only performed on 33 plant species (supplementary Table S12). When samples were identified as containing either ALA or GLA, the intact concentration obtained from the LC-MS/MS analysis (see Quantification by LC-MS/MS) was used. However, when samples contained both, an additional analysis was performed using the GC-MS chromatogram at SIM *m/z* 458. The height (and area) of ALA and GLA injected at the same concentration is more or less the same (see supplementary Fig. S7), which means that the relationship between ALA and GLA is 1 to 1. Therefore, the proportion of the area of both peaks in a sample containing ALA and GLA was determined (i.e., the sum of the area of both peaks was set to 1). Then, the proportions obtained were multiplied by the concentration obtained from the LC-MS/MS analysis to assign the concentrations of ALA and GLA, respectively.

Quantification of ScA and DHGLA. The determination of the concentrations of ScA and DHGLA was also only performed on 33 plant species (supplementary Table S13). When samples contained either ScA or DHGLA, the intact concentration obtained from the LC-MS/MS analysis (see quantification by LC-MS/MS) was used. However, when samples contained both, an additional analysis was performed using the GC-MS chromatogram at SIM *m/z* 486. The response of these two structural isomers in the GC-MS is not equal. When injecting the same concentration of each isomer, the peak size of ScA is 3 times smaller than that of DHGLA (height and area), which can be observed in see supplementary Fig. S8 (i.e., the relationship between ScA and DHGLA is 1 to 3). Therefore, when samples contained both isomers, the first step taken was the normalization of DHGLA, which was achieved by dividing the area of DHGLA by 3. Then, this area was used to calculate the proportion of both peaks in the sample (i.e., the sum of the area of both peaks was set to 1). Finally, the proportions were multiplied by the concentration obtained from the LC-MS/MS analysis to assign the concentrations of ScA and DHGLA, respectively.

Quantification of AA and JuA in *Araucaria araucana*. All samples contained either AA or JuA except for *Araucaria araucana* (see Fig. 3 and supplementary Fig. S3) In this case, to assign the concentration of AA and JuA, the analysis of LC-MS/MS at MRM transition 303/205 was used (see chromatographic conditions used for the identification of structural isomers (LC-MS/MS method 2)). The relationship between AA and JuA injected at the same concentration is 3.5 to 1 (Fig. 3 and supplementary Fig. S3). Therefore, the area of AA in *Araucaria araucana* was divided by 3.5 to normalize the signal response and, later, the proportion of each peak in the sample was determined. Finally, the proportions were multiplied by the concentration obtained from the LC-MS/MS analysis to assign the

concentrations of AA and JuA, respectively. Additionally, the use of the MRM transitions 303/59 and 303/259 yielded similar results.

2. Supplementary Tables

Supplementary Table S1: Concentrations of arachidonic acid or AA (20:4, $\Delta^{5,8,11,14}$, ω-6) and juniperonic acid or JuA (20:4, $\Delta^{5,11,14,17}$, ω-3) (pmol/g plant weight) found in the 71 plant species analyzed. Values are reported as the mean (X) and standard deviation (SD) of at least triplicates (n) of at least one plant species (N).

#	Group	Order	Family	Species	AA (pmol/g PW)		JuA (pmol/g PW)		N,n
					X	SD	X	SD	
1	Angiosperms	Santalales	Viscaceae	*Viscum album* L.	<LOD	-	<LOD	-	1,3
2	Angiosperms	Brassicales	Brassicaceae	*Arabidopsis thaliana* (L.) Heynh.	<LOD	-	<LOD	-	2,5
3	Angiosperms	Malvales	Malvaceae	*Theobroma cacao* L.	<LOD	-	<LOD	-	1,3
4	Angiosperms	Rosales	Cannabaceae	*Cannabis sativa* L.	<LOD	-	<LOD	-	2,5
5	Angiosperms	Malpighiales	Rhizophoraceae	*Rhizophora mangle* L.	<LOD	-	<LOD	-	1,3
6	Angiosperms	Malpighiales	Salicaceae	*Salix glabra* Scop.	<LOD	-	<LOD	-	1,3
7	Angiosperms	Poales	Poaceae	*Sorghum bicolor* (L.) Moench	<LOD	-	<LOD	-	1,3
8	Angiosperms	Poales	Poaceae	*Hakonechloa macra* (Munro) Makino	<LOD	-	<LOD	-	1,3
9	Angiosperms	Poales	Poaceae	*Zea mays* L.	<LOD	-	<LOD	-	1,3
10	Angiosperms	Asparagales	Amaryllidaceae	*Allium sativum* L.	<LOD	-	<LOD	-	1,3
11	Angiosperms	Magnoliales	Magnoliaceae	*Liriodendron tulipifera* L.	<LOD	-	<LOD	-	1,3
12	Angiosperms	Chloranthales	Chloranthaceae	*Chloranthus spicatus* (Thunb.) Makino	<LOD	-	<LOD	-	1,3
13	Angiosperms	Austrobaileyales	Schisandraceae	*Schisandra chinensis* (Turcz.) Baill.	<LOD	-	<LOD	-	1,3
14	Angiosperms	Nymphaeales	Nymphaeaceae	*Nymphaea nouchali* Burm.f.	<LOD	-	<LOD	-	1,3
15	Angiosperms	Amborellales	Amborellaceae	*Amborella trichopoda* Baill.	<LOD	-	<LOD	-	1,3
16	Gymnosperms	Gnetidae	Welwitschiaceae	*Welwitschia mirabilis* Hook.f.	<LOD	-	<LOD	-	1,3
17	Gymnosperms	Pinales	Pinaceae	*Pinus peuce* Griseb.	<LOD	-	12895.8	5450.8	2,5
18	Gymnosperms	Pinales	Pinaceae	*Pinus sylvestris* L.	<LOD	-	76526.6	20022.7	1,3
19	Gymnosperms	Pinales	Pinaceae	*Pinus mugo* Turra	<LOD	-	18467.1	3086.3	1,3
20	Gymnosperms	Pinales	Pinaceae	*Pinus cembra* L.	<LOD	-	601.1	389.6	1,3
21	Gymnosperms	Pinales	Pinaceae	*Larix gmelinii* var. *principis-rupprechtii* (Mayr) Pilg.	<LOD	-	54.4	-	1,3
22	Gymnosperms	Pinales	Pinaceae	*Picea abies* (L.) H.Karst.	<LOD	-	4427.3	692.1	1,3
23	Gymnosperms	Pinales	Pinaceae	*Abies numidica* de Lannoy ex Carrière	<LOD	-	10998.8	6014.5	1,3
24	Gymnosperms	Pinales	Pinaceae	*Abies cephalonica* Loudon	<LOD	-	38959.6	11832.6	1,3
25	Gymnosperms	Pinales	Pinaceae	*Abies koreana* E.H.Wilson	<LOD	-	15811.0	4287.5	1,3
26	Gymnosperms	Cupressales/Pinales	Taxaceae	*Taxus baccata* L.	<LOD	-	<LOD	-	1,4
27	Gymnosperms	Cupressales	Cupressaceae	*Cryptomeria japonica* (Thunb. ex L.f.) D.Don	<LOD	-	100611.8	20144.0	1,3
28	Gymnosperms	Cupressales	Cupressaceae	*Thujopsis dolabrata* (Thunb. ex L.f.) Sieb. & Zucc.	<LOD	-	770.4	547.1	1,3
29	Gymnosperms	Araucariales	Araucariaceae	*Araucaria araucana* (Molina) K.Koch	26773.4	6415.7	80320.2	19247.0	2,5
30	Gymnosperms	Ginkgoales	Ginkgoaceae	*Ginkgo biloba* L.	<LOD	-	216277.7	225967.6	2,5
31	Gymnosperms	Cycadales	Cycadaceae	*Cycas revoluta* Thunb.	<LOD	-	4216.1	3572.2	2,5
32	Monilophytes	Polypodiales	Polypodiaceae	*Polypodium vulgare* L.	44425.8	39444.8	<LOD	-	2,5
33	Monilophytes	Polypodiales	Davalliaceae	*Davallia canariensis* (L.) Sm.	2884.3	1833.5	<LOD	-	1,3
34	Monilophytes	Polypodiales	Tectariaceae	*Tectaria zeylanica* (Houtt.) Sledge	3848.3	2451.8	<LOD	-	1,3
35	Monilophytes	Polypodiales	Dryopteridaceae	*Polystichum aculeatum* (L.) Roth ex Mert.	16165.1	1345.9	<LOD	-	1,3
36	Monilophytes	Polypodiales	Onocleaceae	*Onoclea sensibilis* L.	32079.4	9313.8	<LOD	-	1,3
37	Monilophytes	Polypodiales	Blechnaceae	*Blechnum spicant* (L.) Sm.	7979.1	1227.9	<LOD	-	1,3
38	Monilophytes	Polypodiales	Thelypteridaceae	*Thelypteris palustri* Schott	6753.9	2868.6	<LOD	-	1,3
39	Monilophytes	Polypodiales	Cystopteridaceae	*Gymnocarpium robertianum* (Hoffm.) Newman	12083.0	2316.2	<LOD	-	1,3
40	Monilophytes	Polypodiales	Aspleniaceae	*Asplenium trichomanes* L.	20835.6	5290.8	<LOD	-	1,3
41	Monilophytes	Polypodiales	Aspleniaceae	*Phyllitis scolopendrium* (L.) Newman	3516.8	1514.7	<LOD	-	1,3
42	Monilophytes	Polypodiales	Pteridaceae	*Adiantum venustum* D. Don	75994.9	14982.5	<LOD	-	1,3
43	Monilophytes	Cyatheales	Cyatheaceae	*Sphaeropteris cooperi* (F.Muell.) R.M.Tryon	4784.5	1187.0	<LOD	-	1,3
44	Monilophytes	Salviniales	Salviniaceae	*Salvinia natans* (L.) All.	13175.0	4809.8	<LOD	-	1,3
45	Monilophytes	Salviniales	Salviniaceae	*Salvinia molesta* D. S. Mitch.	307394.3	236171.1	<LOD	-	2,5
46	Monilophytes	Schizaeales	Anemiaceae	*Anemia phyllitidis* (L.) Sw.	12143.7	7263.7	<LOD	-	1,3
47	Monilophytes	Schizaeales	Lygodiaceae	*Lygodium volubile* Sw.	64628.3	31260.0	<LOD	-	1,3
48	Monilophytes	Osmundales	Osmundaceae	*Osmunda regalis* L.	1386.0	632.9	<LOD	-	1,3
49	Monilophytes	Marattiales	Marattiaceae	*Angiopteris evecta* (G.Forst.) Hoffm.	6125.1	2080.0	<LOD	-	1,3
50	Monilophytes	Equisetales	Equisetaceae	*Equisetum trachyodon* (A. Braun) W.D.J. Koch	<LOD	-	4768.5	1854.4	1,3
51	Monilophytes	Psilotales	Psilotaceae	*Psilotum nudum* (L.) P. Beauv.	<LOD	-	<LOD	-	1,3
52	Lycophytes	Selaginellales	Selaginellaceae	*Selaginella moellendorffii* Hieron.	<LOD	-	9985.0	4131.1	1,3
53	Lycophytes	Selaginellales	Selaginellaceae	*Selaginella pallescens* (C.Presl) Spring	<LOD	-	15352.2	10680.3	2,6
54	Lycophytes	Lycopodiales	Lycopodiaceae	*Huperzia phlegmaria* (L.) Rothm.	83663.7	47441.0	<LOD	-	1,3
55	Hornworts	Anthocerotales	Anthocerotaceae	*Anthoceros agrestis* Paton nom. cons. prop.	69691.7	21150.4	<LOD	-	1,4
56	Hornworts	Anthocerotales	Anthocerotaceae	*Anthoceros punctatus* L.	24687.6	14397.5	<LOD	-	1,3
57	Hornworts	Notothyladales	Notothyladaceae	*Phaeoceros laevis* (L.) Prosk.	316375.9	81404.2	<LOD	-	1,3
58	Mosses	Funariales	Funariaceae	*Physcomitrella patens* (Hedw.) Bruch & Schimp.	2648874.2	3162852.2	<LOD	-	3,6
59	Mosses	Funariales	Funariaceae	*Funaria hygrometrica* Hedw.	898972.3	62534.5	<LOD	-	1,3
60	Mosses	Polytrichales	Polytrichaceae	*Polytrichum juniperinum* Hedw.	35394.6	20181.8	<LOD	-	1,3
61	Mosses	Hedwigiales	Hedwigiaceae	*Hedwigia ciliata* (Hedw.) P.Beauv.	20046.8	8190.9	<LOD	-	1,3
62	Mosses	Hypnales	Hylocomiaceae	*Hylocomium splendens* (Hedw.) Schimp. in B.S.G.	86608.3	59809.8	<LOD	-	1,3
63	Liverworts	Marchantiales	Conocephalaceae	*Conocephalum conicum* (Linnaeus) Lindb.	1233225.6	177805.3	<LOD	-	1,3
64	Liverworts	Marchantiales	Marchantiaceae	*Marchantia polymorpha* L.	903496.0	298986.3	<LOD	-	1,3
65	Liverworts	Marchantiales	Ricciaceae	*Riccia fluitans* L.	452189.2	293085.6	<LOD	-	1,3
66	Algae	Charophytes	Characeae	*Chara vulgaris* Linnaeus	203074.1	150828.5	<LOD	-	2,5
67	Algae	Charophytes	Klebsormidiaceae	*Klebsormidium elegans* Lokhorst	<LOD	-	<LOD	-	1,3
68	Algae	Chlorophyta	Caulerpaceae	*Caulerpa prolifera* (P. Forsskal) Lamouroux	74711.0	46106.2	<LOD	-	1,3
69	Algae	Rhodophyta	Halymeniaceae	*Halymenia floresii* (Clemente y Rubio) C. Agardh	9959.3	1220.6	<LOD	-	1,3
70	Lichen	Lecanorales	Parmeliaceae	*Cetraria islandica* (L.) Ach.	2708.8	1574.4	<LOD	-	1,3
71	Lichen	Teloschistales	Teloschistaceae	*Xanthoria parietina* (L.) Th. Fr.	24535.4	7121.4	<LOD	-	1,3

Supplementary Table S2: Concentrations of 1/2-arachidonoyl glycerol or 1/2-AG and 1/2-juniperoyl glycerol or 1/2-JG (pmol/g plant weight) found in the 71 plant species analyzed. Values are reported as the mean (X) and standard deviation (SD) of at least triplicates (n) of at least one plant species (N).

#	Group	Order	Family	Species	1/2-AG (pmol/g PW)		1/2-JG (pmol/g PW)		N,n
					X	SD	X	SD	
1	Angiosperms	Santalales	Viscaceae	*Viscum album* L.	<LOD	-	<LOD	-	1,3
2	Angiosperms	Brassicales	Brassicaceae	*Arabidopsis thaliana* (L.) Heynh.	<LOD	-	<LOD	-	2,5
3	Angiosperms	Malvales	Malvaceae	*Theobroma cacao* L.	<LOD	-	<LOD	-	1,3
4	Angiosperms	Rosales	Cannabaceae	*Cannabis sativa* L.	<LOD	-	<LOD	-	2,5
5	Angiosperms	Malpighiales	Rhizophoraceae	*Rhizophora mangle* L.	<LOD	-	<LOD	-	1,3
6	Angiosperms	Malpighiales	Salicaceae	*Salix glabra* Scop.	<LOD	-	<LOD	-	1,3
7	Angiosperms	Poales	Poaceae	*Sorghum bicolor* (L.) Moench	<LOD	-	<LOD	-	1,3
8	Angiosperms	Poales	Poaceae	*Hakonechloa macra* (Munro) Makino	<LOD	-	<LOD	-	1,3
9	Angiosperms	Poales	Poaceae	*Zea mays* L.	<LOD	-	<LOD	-	1,3
10	Angiosperms	Asparagales	Amaryllidaceae	*Allium sativum* L.	<LOD	-	<LOD	-	1,3
11	Angiosperms	Magnoliales	Magnoliaceae	*Liriodendron tulipifera* L.	<LOD	-	<LOD	-	1,3
12	Angiosperms	Chloranthales	Chloranthaceae	*Chloranthus spicatus* (Thunb.) Makino	<LOD	-	<LOD	-	1,3
13	Angiosperms	Austrobaileyales	Schisandraceae	*Schisandra chinensis* (Turcz.) Baill.	<LOD	-	<LOD	-	1,3
14	Angiosperms	Nymphaeales	Nymphaeaceae	*Nymphaea nouchali* Burm.f.	<LOD	-	<LOD	-	1,3
15	Angiosperms	Amborellales	Amborellaceae	*Amborella trichopoda* Baill.	<LOD	-	<LOD	-	1,3
16	Gymnosperms	Gnetidae	Welwitschiaceae	*Welwitschia mirabilis* Hook.f.	<LOD	-	<LOD	-	1,3
17	Gymnosperms	Pinales	Pinaceae	*Pinus peuce* Griseb.	<LOD	-	468.7	176.7	2,5
18	Gymnosperms	Pinales	Pinaceae	*Pinus sylvestris* L.	<LOD	-	2375.0	1748.7	1,3
19	Gymnosperms	Pinales	Pinaceae	*Pinus mugo* Turra	<LOD	-	600.8	409.1	1,3
20	Gymnosperms	Pinales	Pinaceae	*Pinus cembra* L.	<LOD	-	55.8	51.1	1,3
21	Gymnosperms	Pinales	Pinaceae	*Larix gmelinii* var. *principis-rupprechtii* (Mayr) Pilg.	<LOD	-	37.4	59.3	1,3
22	Gymnosperms	Pinales	Pinaceae	*Picea abies* (L.) H.Karst.	<LOD	-	308.5	214.1	1,3
23	Gymnosperms	Pinales	Pinaceae	*Abies numidica* de Lannoy ex Carrière	<LOD	-	374.1	155.4	1,3
24	Gymnosperms	Pinales	Pinaceae	*Abies cephalonica* Loudon	<LOD	-	2695.1	1605.7	1,3
25	Gymnosperms	Pinales	Pinaceae	*Abies koreana* E.H.Wilson	<LOD	-	697.4	458.5	1,3
26	Gymnosperms	Cupressales/Pinales	Taxaceae	*Taxus baccata* L.	<LOD	-	<LOD	-	1,4
27	Gymnosperms	Cupressales	Cupressaceae	*Cryptomeria japonica* (Thunb. ex L.f.) D.Don	<LOD	-	2163.8	875.9	1,3
28	Gymnosperms	Cupressales	Cupressaceae	*Thujopsis dolabrata* (Thunb. ex L.f.) Sieb. & Zucc.	<LOD	-	<LOD	-	1,3
29	Gymnosperms	Araucariales	Araucariaceae	*Araucaria araucana* (Molina) K.Koch	<LOD	-	3208.1	1765.0	2,5
30	Gymnosperms	Ginkgoales	Ginkgoaceae	*Ginkgo biloba* L.	<LOD	-	2795.6	1420.4	2,5
31	Gymnosperms	Cycadales	Cycadaceae	*Cycas revoluta* Thunb.	<LOD	-	118.2	90.3	2,5
32	Monilophytes	Polypodiales	Polypodiaceae	*Polypodium vulgare* L.	6909.4	4969.8	<LOD	-	2,5
33	Monilophytes	Polypodiales	Davalliaceae	*Davallia canariensis* (L.) Sm.	1298.1	776.9	<LOD	-	1,3
34	Monilophytes	Polypodiales	Tectariaceae	*Tectaria zeylanica* (Houtt.) Sledge	3233.2	1338.4	<LOD	-	1,3
35	Monilophytes	Polypodiales	Dryopteridaceae	*Polystichum aculeatum* (L.) Roth ex Mert.	5162.6	903.0	<LOD	-	1,3
36	Monilophytes	Polypodiales	Onocleaceae	*Onoclea sensibilis* L.	2085.9	66.8	<LOD	-	1,3
37	Monilophytes	Polypodiales	Blechnaceae	*Blechnum spicant* (L.) Sm.	2995.3	1000.1	<LOD	-	1,3
38	Monilophytes	Polypodiales	Thelypteridaceae	*Thelypteris palustri* Schott	2851.6	842.3	<LOD	-	1,3
39	Monilophytes	Polypodiales	Cystopteridaceae	*Gymnocarpium robertianum* (Hoffm.) Newman	3616.1	587.8	<LOD	-	1,3
40	Monilophytes	Polypodiales	Aspleniaceae	*Asplenium trichomanes* L.	16014.1	2377.3	<LOD	-	1,3
41	Monilophytes	Polypodiales	Aspleniaceae	*Phyllitis scolopendrium* (L.) Newman	1698.1	426.1	<LOD	-	1,3
42	Monilophytes	Polypodiales	Pteridaceae	*Adiantum venustum* D. Don	10783.1	8047.4	<LOD	-	1,3
43	Monilophytes	Cyatheales	Cyatheaceae	*Sphaeropteris cooperi* (F.Muell.) R.M.Tryon	166.1	11.0	<LOD	-	1,3
44	Monilophytes	Salviniales	Salviniaceae	*Salvinia natans* (L.) All.	2067.9	917.0	<LOD	-	1,3
45	Monilophytes	Salviniales	Salviniaceae	*Salvinia molesta* D. S. Mitch.	11227.2	6519.6	<LOD	-	2,5
46	Monilophytes	Schizaeales	Anemiaceae	*Anemia phyllitidis* (L.) Sw.	1209.6	509.7	<LOD	-	1,3
47	Monilophytes	Schizaeales	Lygodiaceae	*Lygodium volubile* Sw.	1182.9	642.6	<LOD	-	1,3
48	Monilophytes	Osmundales	Osmundaceae	*Osmunda regalis* L.	409.9	18.2	<LOD	-	1,3
49	Monilophytes	Marattiales	Marattiaceae	*Angiopteris evecta* (G.Forst.) Hoffm.	379.7	77.6	<LOD	-	1,3
50	Monilophytes	Equisetales	Equisetaceae	*Equisetum trachyodon* (A. Braun) W.D.J. Koch	<LOD	-	2748.9	777.1	1,3
51	Monilophytes	Psilotales	Psilotaceae	*Psilotum nudum* (L.) P. Beauv.	<LOD	-	176.1	23.2	1,3
52	Lycophytes	Selaginellales	Selaginellaceae	*Selaginella moellendorffii* Hieron.	<LOD	-	1818.6	216.2	1,3
53	Lycophytes	Selaginellales	Selaginellaceae	*Selaginella pallescens* (C.Presl) Spring	<LOD	-	4394.7	2447.5	2,6
54	Lycophytes	Lycopodiales	Lycopodiaceae	*Huperzia phlegmaria* (L.) Rothm.	2098.9	1242.9	<LOD	-	1,3
55	Hornworts	Anthocerotales	Anthocerotaceae	*Anthoceros agrestis* Paton nom. cons. prop.	51037.2	24593.3	<LOD	-	1,4
56	Hornworts	Anthocerotales	Anthocerotaceae	*Anthoceros punctatus* L.	21241.6	684.0	<LOD	-	1,3
57	Hornworts	Notothyladales	Notothyladaceae	*Phaeoceros laevis* (L.) Prosk.	37873.5	8043.1	<LOD	-	1,3
58	Mosses	Funariales	Funariaceae	*Physcomitrella patens* (Hedw.) Bruch & Schimp.	286570.7	157384.1	<LOD	-	3,6
59	Mosses	Funariales	Funariaceae	*Funaria hygrometrica* Hedw.	47555.5	12612.6	<LOD	-	1,3
60	Mosses	Polytrichales	Polytrichaceae	*Polytrichum juniperinum* Hedw.	2472.3	1324.8	<LOD	-	1,3
61	Mosses	Hedwigiales	Hedwigiaceae	*Hedwigia ciliata* (Hedw.) P.Beauv.	728.5	324.5	<LOD	-	1,3
62	Mosses	Hypnales	Hylocomiaceae	*Hylocomium splendens* (Hedw.) Schimp. in B.S.G.	6307.5	5033.3	<LOD	-	1,3
63	Liverworts	Marchantiales	Conocephalaceae	*Conocephalum conicum* (Linnaeus) Lindb.	55780.9	14517.9	<LOD	-	1,3
64	Liverworts	Marchantiales	Marchantiaceae	*Marchantia polymorpha* L.	81990.1	34970.3	<LOD	-	1,3
65	Liverworts	Marchantiales	Ricciaceae	*Riccia fluitans* L.	58983.4	39632.9	<LOD	-	1,3
66	Algae	Charophytes	Characeae	*Chara vulgaris* Linnaeus	10047.4	5943.4	<LOD	-	2,5
67	Algae	Charophytes	Klebsormidiaceae	*Klebsormidium elegans* Lokhorst	<LOD	-	<LOD	-	1,3
68	Algae	Chlorophyta	Caulerpaceae	*Caulerpa prolifera* (P. Forsskal) Lamouroux	<LOD	-	<LOD	-	1,3
69	Algae	Rhodophyta	Halymeniaceae	*Halymenia floresii* (Clemente y Rubio) C. Agardh	1033.0	422.5	<LOD	-	1,3
70	Lichen	Lecanorales	Parmeliaceae	*Cetraria islandica* (L.) Ach.	<LOD	-	<LOD	-	1,3
71	Lichen	Teloschistales	Teloschistaceae	*Xanthoria parietina* (L.) Th. Fr.	1112.4	75.4	<LOD	-	1,3

Supplementary Table S3: Concentrations of arachidonoyl ethanolamide or AEA and juniperoyl ethanolamide JEA (pmol/g plant weight) found in the 71 plant species analyzed. Values are reported as the mean (X) and standard deviation (SD) of at least triplicates (n) of at least one plant species (N).

#	Group	Order	Family	Species	AEA (pmol/g PW)		JEA pmol/g DW (X)		N,n
					X	SD	X	SD	
1	Angiosperms	Santalales	Viscaceae	*Viscum album* L.	<LOD	-	<LOD	-	1,3
2	Angiosperms	Brassicales	Brassicaceae	*Arabidopsis thaliana* (L.) Heynh.	<LOD	-	<LOD	-	2,5
3	Angiosperms	Malvales	Malvaceae	*Theobroma cacao* L.	<LOD	-	<LOD	-	1,3
4	Angiosperms	Rosales	Cannabaceae	*Cannabis sativa* L.	<LOD	-	<LOD	-	2,5
5	Angiosperms	Malpighiales	Rhizophoraceae	*Rhizophora mangle* L.	<LOD	-	<LOD	-	1,3
6	Angiosperms	Malpighiales	Salicaceae	*Salix glabra* Scop.	<LOD	-	<LOD	-	1,3
7	Angiosperms	Poales	Poaceae	*Sorghum bicolor* (L.) Moench	<LOD	-	<LOD	-	1,3
8	Angiosperms	Poales	Poaceae	*Hakonechloa macra* (Munro) Makino	<LOD	-	<LOD	-	1,3
9	Angiosperms	Poales	Poaceae	*Zea mays* L.	<LOD	-	<LOD	-	1,3
10	Angiosperms	Asparagales	Amaryllidaceae	*Allium sativum* L.	<LOD	-	<LOD	-	1,3
11	Angiosperms	Magnoliales	Magnoliaceae	*Liriodendron tulipifera* L.	<LOD	-	<LOD	-	1,3
12	Angiosperms	Chloranthales	Chloranthaceae	*Chloranthus spicatus* (Thunb.) Makino	<LOD	-	<LOD	-	1,3
13	Angiosperms	Austrobaileyales	Schisandraceae	*Schisandra chinensis* (Turcz.) Baill.	<LOD	-	<LOD	-	1,3
14	Angiosperms	Nymphaeales	Nymphaeaceae	*Nymphaea nouchali* Burm.f.	<LOD	-	<LOD	-	1,3
15	Angiosperms	Amborellales	Amborellaceae	*Amborella trichopoda* Baill.	<LOD	-	<LOD	-	1,3
16	Gymnosperms	Gnetidae	Welwitschiaceae	*Welwitschia mirabilis* Hook.f.	<LOD	-	<LOD	-	1,3
17	Gymnosperms	Pinales	Pinaceae	*Pinus peuce* Griseb.	<LOD	-	23.8	19.4	2,5
18	Gymnosperms	Pinales	Pinaceae	*Pinus sylvestris* L.	<LOD	-	229.8	17.4	1,3
19	Gymnosperms	Pinales	Pinaceae	*Pinus mugo* Turra	<LOD	-	<LOD	-	1,3
20	Gymnosperms	Pinales	Pinaceae	*Pinus cembra* L.	<LOD	-	2.5	0.4	1,3
21	Gymnosperms	Pinales	Pinaceae	*Larix gmelinii* var. *principis-rupprechtii* (Mayr) Pilg.	<LOD	-	27.6	45.1	1,3
22	Gymnosperms	Pinales	Pinaceae	*Picea abies* (L.) H.Karst.	<LOD	-	0.8	0.3	1,3
23	Gymnosperms	Pinales	Pinaceae	*Abies numidica* de Lannoy ex Carrière	<LOD	-	7.2	4.7	1,3
24	Gymnosperms	Pinales	Pinaceae	*Abies cephalonica* Loudon	<LOD	-	7.4	6.0	1,3
25	Gymnosperms	Pinales	Pinaceae	*Abies koreana* E.H.Wilson	<LOD	-	4.6	1.0	1,3
26	Gymnosperms	Cupressales/Pinales	Taxaceae	*Taxus baccata* L.	<LOD	-	<LOD	-	1,4
27	Gymnosperms	Cupressales	Cupressaceae	*Cryptomeria japonica* (Thunb. ex L.f.) D.Don	<LOD	-	69.3	4.5	1,3
28	Gymnosperms	Cupressales	Cupressaceae	*Thujopsis dolabrata* (Thunb. ex L.f.) Sieb. & Zucc.	<LOD	-	<LOD	-	1,3
29	Gymnosperms	Araucariales	Araucariaceae	*Araucaria araucana* (Molina) K.Koch	<LOD	-	118.1	50.1	2,5
30	Gymnosperms	Ginkgoales	Ginkgoaceae	*Ginkgo biloba* L.	<LOD	-	254.3	47.5	2,5
31	Gymnosperms	Cycadales	Cycadaceae	*Cycas revoluta* Thunb.	<LOD	-	11.8	8.8	2,5
32	Monilophytes	Polypodiales	Polypodiaceae	*Polypodium vulgare* L.	11.0	5.2	<LOD	-	2,5
33	Monilophytes	Polypodiales	Davalliaceae	*Davallia canariensis* (L.) Sm.	<LOD	-	<LOD	-	1,3
34	Monilophytes	Polypodiales	Tectariaceae	*Tectaria zeylanica* (Houtt.) Sledge	<LOD	-	<LOD	-	1,3
35	Monilophytes	Polypodiales	Dryopteridaceae	*Polystichum aculeatum* (L.) Roth ex Mert.	5.0	2.8	<LOD	-	1,3
36	Monilophytes	Polypodiales	Onocleaceae	*Onoclea sensibilis* L.	<LOD	-	<LOD	-	1,3
37	Monilophytes	Polypodiales	Blechnaceae	*Blechnum spicant* (L.) Sm.	<LOD	-	<LOD	-	1,3
38	Monilophytes	Polypodiales	Thelypteridaceae	*Thelypteris palustri* Schott	7.2	2.3	<LOD	-	1,3
39	Monilophytes	Polypodiales	Cystopteridaceae	*Gymnocarpium robertianum* (Hoffm.) Newman	7.7	2.0	<LOD	-	1,3
40	Monilophytes	Polypodiales	Aspleniaceae	*Asplenium trichomanes* L.	6.4	0.3	<LOD	-	1,3
41	Monilophytes	Polypodiales	Aspleniaceae	*Phyllitis scolopendrium* (L.) Newman	3.8	1.7	<LOD	-	1,3
42	Monilophytes	Polypodiales	Pteridaceae	*Adiantum venustum* D. Don	<LOD	-	<LOD	-	1,3
43	Monilophytes	Cyatheales	Cyatheaceae	*Sphaeropteris cooperi* (F.Muell.) R.M.Tryon	<LOD	-	<LOD	-	1,3
44	Monilophytes	Salviniales	Salviniaceae	*Salvinia natans* (L.) All.	1.2	0.7	<LOD	-	1,3
45	Monilophytes	Salviniales	Salviniaceae	*Salvinia molesta* D. S. Mitch.	102.6	75.2	<LOD	-	2,5
46	Monilophytes	Schizaeales	Anemiaceae	*Anemia phyllitidis* (L.) Sw.	56.0	8.1	<LOD	-	1,3
47	Monilophytes	Schizaeales	Lygodiaceae	*Lygodium volubile* Sw.	<LOD	-	<LOD	-	1,3
48	Monilophytes	Osmundales	Osmundaceae	*Osmunda regalis* L.	<LOD	-	<LOD	-	1,3
49	Monilophytes	Marattiales	Marattiaceae	*Angiopteris evecta* (G.Forst.) Hoffm.	6.9	2.4	<LOD	-	1,3
50	Monilophytes	Equisetales	Equisetaceae	*Equisetum trachyodon* (A. Braun) W.D.J. Koch	<LOD	-	7.4	2.9	1,3
51	Monilophytes	Psilotales	Psilotaceae	*Psilotum nudum* (L.) P. Beauv.	<LOD	-	4.7	2.2	1,3
52	Lycophytes	Selaginellales	Selaginellaceae	*Selaginella moellendorffii* Hieron.	<LOD	-	6.5	2.4	1,3
53	Lycophytes	Selaginellales	Selaginellaceae	*Selaginella pallescens* (C.Presl) Spring	<LOD	-	15.4	13.8	2,6
54	Lycophytes	Lycopodiales	Lycopodiaceae	*Huperzia phlegmaria* (L.) Rothm.	10.2	4.2	<LOD	-	1,3
55	Hornworts	Anthocerotales	Anthocerotaceae	*Anthoceros agrestis* Paton nom. cons. prop.	258.9	23.9	<LOD	-	1,4
56	Hornworts	Anthocerotales	Anthocerotaceae	*Anthoceros punctatus* L.	<LOD	-	<LOD	-	1,3
57	Hornworts	Notothyladales	Notothyladaceae	*Phaeoceros laevis* (L.) Prosk.	694.4	225.7	<LOD	-	1,3
58	Mosses	Funariales	Funariaceae	*Physcomitrella patens* (Hedw.) Bruch & Schimp.	694.4	678.6	<LOD	-	3,6
59	Mosses	Funariales	Funariaceae	*Funaria hygrometrica* Hedw.	148.7	32.8	<LOD	-	1,3
60	Mosses	Polytrichales	Polytrichaceae	*Polytrichum juniperinum* Hedw.	35.9	13.0	<LOD	-	1,3
61	Mosses	Hedwigiales	Hedwigiaceae	*Hedwigia ciliata* (Hedw.) P.Beauv.	15.4	0.5	<LOD	-	1,3
62	Mosses	Hypnales	Hylocomiaceae	*Hylocomium splendens* (Hedw.) Schimp. in B.S.G.	215.5	143.0	<LOD	-	1,3
63	Liverworts	Marchantiales	Conocephalaceae	*Conocephalum conicum* (Linnaeus) Lindb.	518.3	113.2	<LOD	-	1,3
64	Liverworts	Marchantiales	Marchantiaceae	*Marchantia polymorpha* L.	891.7	285.3	<LOD	-	1,3
65	Liverworts	Marchantiales	Ricciaceae	*Riccia fluitans* L.	460.2	245.1	<LOD	-	1,3
66	Algae	Charophytes	Characeae	*Chara vulgaris* Linnaeus	28.9	19.0	<LOD	-	2,5
67	Algae	Charophytes	Klebsormidiaceae	*Klebsormidium elegans* Lokhorst	<LOD	-	<LOD	-	1,3
68	Algae	Chlorophyta	Caulerpaceae	*Caulerpa prolifera* (P. Forsskal) Lamouroux	<LOD	-	<LOD	-	1,3
69	Algae	Rhodophyta	Halymeniaceae	*Halymenia floresii* (Clemente y Rubio) C. Agardh	<LOD	-	<LOD	-	1,3
70	Lichen	Lecanorales	Parmeliaceae	*Cetraria islandica* (L.) Ach.	<LOD	-	<LOD	-	1,3
71	Lichen	Teloschistales	Teloschistaceae	*Xanthoria parietina* (L.) Th. Fr.	<LOD	-	<LOD	-	1,3

Supplementary Table S4: Concentrations of myristoyl ethanolamide or MEA (pmol/g plant weight) found in the 71 plant species analyzed. Values are reported as the mean (X) and standard deviation (SD) of at least triplicates (n) of at least one plant species (N).

#	Group	Order	Family	Species	MEA (pmol/g PW)		N,n
					X	SD	
1	Angiosperms	Santalales	Viscaceae	*Viscum album* L.	<LOD	-	1,3
2	Angiosperms	Brassicales	Brassicaceae	*Arabidopsis thaliana* (L.) Heynh.	48.4	31.0	2,5
3	Angiosperms	Malvales	Malvaceae	*Theobroma cacao* L.	<LOD	-	1,3
4	Angiosperms	Rosales	Cannabaceae	*Cannabis sativa* L.	3.7	3.0	2,5
5	Angiosperms	Malpighiales	Rhizophoraceae	*Rhizophora mangle* L.	5.3	3.1	1,3
6	Angiosperms	Malpighiales	Salicaceae	*Salix glabra* Scop.	<LOD	-	1,3
7	Angiosperms	Poales	Poaceae	*Sorghum bicolor* (L.) Moench	8.7	4.3	1,3
8	Angiosperms	Poales	Poaceae	*Hakonechloa macra* (Munro) Makino	9.5	2.6	1,3
9	Angiosperms	Poales	Poaceae	*Zea mays* L.	1.9	0.2	1,3
10	Angiosperms	Asparagales	Amaryllidaceae	*Allium sativum* L.	57.5	20.5	1,3
11	Angiosperms	Magnoliales	Magnoliaceae	*Liriodendron tulipifera* L.	<LOD	-	1,3
12	Angiosperms	Chloranthales	Chloranthaceae	*Chloranthus spicatus* (Thunb.) Makino	<LOD	-	1,3
13	Angiosperms	Austrobaileyales	Schisandraceae	*Schisandra chinensis* (Turcz.) Baill.	<LOD	-	1,3
14	Angiosperms	Nymphaeales	Nymphaeaceae	*Nymphaea nouchali* Burm.f.	11.9	7.1	1,3
15	Angiosperms	Amborellales	Amborellaceae	*Amborella trichopoda* Baill.	4.7	2.4	1,3
16	Gymnosperms	Gnetidae	Welwitschiaceae	*Welwitschia mirabilis* Hook.f.	20.1	14.4	1,3
17	Gymnosperms	Pinales	Pinaceae	*Pinus peuce* Griseb.	<LOD	-	2,5
18	Gymnosperms	Pinales	Pinaceae	*Pinus sylvestris* L.	<LOD	-	1,3
19	Gymnosperms	Pinales	Pinaceae	*Pinus mugo* Turra	<LOD	-	1,3
20	Gymnosperms	Pinales	Pinaceae	*Pinus cembra* L.	<LOD	-	1,3
21	Gymnosperms	Pinales	Pinaceae	*Larix gmelinii* var. *principis-rupprechtii* (Mayr) Pilg.	<LOD	-	1,3
22	Gymnosperms	Pinales	Pinaceae	*Picea abies* (L.) H.Karst.	<LOD	-	1,3
23	Gymnosperms	Pinales	Pinaceae	*Abies numidica* de Lannoy ex Carrière	<LOD	-	1,3
24	Gymnosperms	Pinales	Pinaceae	*Abies cephalonica* Loudon	<LOD	-	1,3
25	Gymnosperms	Pinales	Pinaceae	*Abies koreana* E.H.Wilson	<LOD	-	1,3
26	Gymnosperms	Cupressales/Pinales	Taxaceae	*Taxus baccata* L.	1.6	1.4	1,4
27	Gymnosperms	Cupressales	Cupressaceae	*Cryptomeria japonica* (Thunb. ex L.f.) D.Don	<LOD	-	1,3
28	Gymnosperms	Cupressales	Cupressaceae	*Thujopsis dolabrata* (Thunb. ex L.f.) Sieb. & Zucc.	<LOD	-	1,3
29	Gymnosperms	Araucariales	Araucariaceae	*Araucaria araucana* (Molina) K.Koch	<LOD	-	2,5
30	Gymnosperms	Ginkgoales	Ginkgoaceae	*Ginkgo biloba* L.	5.6	1.0	2,5
31	Gymnosperms	Cycadales	Cycadaceae	*Cycas revoluta* Thunb.	<LOD	-	2,5
32	Monilophytes	Polypodiales	Polypodiaceae	*Polypodium vulgare* L.	13.4	3.8	2,5
33	Monilophytes	Polypodiales	Davalliaceae	*Davallia canariensis* (L.) Sm.	<LOD	-	1,3
34	Monilophytes	Polypodiales	Tectariaceae	*Tectaria zeylanica* (Houtt.) Sledge	<LOD	-	1,3
35	Monilophytes	Polypodiales	Dryopteridaceae	*Polystichum aculeatum* (L.) Roth ex Mert.	32.9	35.6	1,3
36	Monilophytes	Polypodiales	Onocleaceae	*Onoclea sensibilis* L.	<LOD	-	1,3
37	Monilophytes	Polypodiales	Blechnaceae	*Blechnum spicant* (L.) Sm.	<LOD	-	1,3
38	Monilophytes	Polypodiales	Thelypteridaceae	*Thelypteris palustri* Schott	<LOD	-	1,3
39	Monilophytes	Polypodiales	Cystopteridaceae	*Gymnocarpium robertianum* (Hoffm.) Newman	6.5	5.8	1,3
40	Monilophytes	Polypodiales	Aspleniaceae	*Asplenium trichomanes* L.	3.5	2.2	1,3
41	Monilophytes	Polypodiales	Aspleniaceae	*Phyllitis scolopendrium* (L.) Newman	5.7	2.5	1,3
42	Monilophytes	Polypodiales	Pteridaceae	*Adiantum venustum* D. Don	1.6	1.5	1,3
43	Monilophytes	Cyatheales	Cyatheaceae	*Sphaeropteris cooperi* (F.Muell.) R.M.Tryon	6.3	3.6	1,3
44	Monilophytes	Salviniales	Salviniaceae	*Salvinia natans* (L.) All.	13.8	13.2	1,3
45	Monilophytes	Salviniales	Salviniaceae	*Salvinia molesta* D. S. Mitch.	57.8	70.0	2,5
46	Monilophytes	Schizaeales	Anemiaceae	*Anemia phyllitidis* (L.) Sw.	<LOD	-	1,3
47	Monilophytes	Schizaeales	Lygodiaceae	*Lygodium volubile* Sw.	<LOD	-	1,3
48	Monilophytes	Osmundales	Osmundaceae	*Osmunda regalis* L.	<LOD	-	1,3
49	Monilophytes	Marattiales	Marattiaceae	*Angiopteris evecta* (G.Forst.) Hoffm.	<LOD	-	1,3
50	Monilophytes	Equisetales	Equisetaceae	*Equisetum trachyodon* (A. Braun) W.D.J. Koch	<LOD	-	1,3
51	Monilophytes	Psilotales	Psilotaceae	*Psilotum nudum* (L.) P. Beauv.	<LOD	-	1,3
52	Lycophytes	Selaginellales	Selaginellaceae	*Selaginella moellendorffii* Hieron.	<LOD	-	1,3
53	Lycophytes	Selaginellales	Selaginellaceae	*Selaginella pallescens* (C.Presl) Spring	25.2	19.3	2,6
54	Lycophytes	Lycopodiales	Lycopodiaceae	*Huperzia phlegmaria* (L.) Rothm.	<LOD	-	1,3
55	Hornworts	Anthocerotales	Anthocerotaceae	*Anthoceros agrestis* Paton nom. cons. prop.	58.8	24.4	1,4
56	Hornworts	Anthocerotales	Anthocerotaceae	*Anthoceros punctatus* L.	330.9	204.4	1,3
57	Hornworts	Notothyladales	Notothyladaceae	*Phaeoceros laevis* (L.) Prosk.	72.4	20.0	1,3
58	Mosses	Funariales	Funariaceae	*Physcomitrella patens* (Hedw.) Bruch & Schimp.	209.5	238.3	3,6
59	Mosses	Funariales	Funariaceae	*Funaria hygrometrica* Hedw.	14.4	9.6	1,3
60	Mosses	Polytrichales	Polytrichaceae	*Polytrichum juniperinum* Hedw.	0.2	0.3	1,3
61	Mosses	Hedwigiales	Hedwigiaceae	*Hedwigia ciliata* (Hedw.) P.Beauv.	<LOD	-	1,3
62	Mosses	Hypnales	Hylocomiaceae	*Hylocomium splendens* (Hedw.) Schimp. in B.S.G.	<LOD	-	1,3
63	Liverworts	Marchantiales	Conocephalaceae	*Conocephalum conicum* (Linnaeus) Lindb.	5.8	1.6	1,3
64	Liverworts	Marchantiales	Marchantiaceae	*Marchantia polymorpha* L.	38.1	29.5	1,3
65	Liverworts	Marchantiales	Ricciaceae	*Riccia fluitans* L.	137.7	114.4	1,3
66	Algae	Charophytes	Characeae	*Chara vulgaris* Linnaeus	5.2	0.1	2,5
67	Algae	Charophytes	Klebsormidiaceae	*Klebsormidium elegans* Lokhorst	767.9	607.9	1,3
68	Algae	Chlorophyta	Caulerpaceae	*Caulerpa prolifera* (P. Forsskal) Lamouroux	<LOD	-	1,3
69	Algae	Rhodophyta	Halymeniaceae	*Halymenia floresii* (Clemente y Rubio) C. Agardh	18.3	8.1	1,3
70	Lichen	Lecanorales	Parmeliaceae	*Cetraria islandica* (L.) Ach.	<LOD	-	1,3
71	Lichen	Teloschistales	Teloschistaceae	*Xanthoria parietina* (L.) Th. Fr.	11.6	3.7	1,3

Supplementary Table S5: Concentrations of linoleoyl ethanolamide or LEA (pmol/g plant weight) found in the 71 plant species analyzed. Values are reported as the mean (X) and standard deviation (SD) of at least triplicates (n) of at least one plant species (N).

#	Group	Order	Family	Species	LEA (pmol/g PW)		N,n
					X	SD	
1	Angiosperms	Santalales	Viscaceae	*Viscum album* L.	9051.8	2222.5	1,3
2	Angiosperms	Brassicales	Brassicaceae	*Arabidopsis thaliana* (L.) Heynh.	17710.2	11057.2	2,5
3	Angiosperms	Malvales	Malvaceae	*Theobroma cacao* L.	1870.1	402.6	1,3
4	Angiosperms	Rosales	Cannabaceae	*Cannabis sativa* L.	416.5	110.6	2,5
5	Angiosperms	Malpighiales	Rhizophoraceae	*Rhizophora mangle* L.	433.2	119.0	1,3
6	Angiosperms	Malpighiales	Salicaceae	*Salix glabra* Scop.	5250.1	1623.1	1,3
7	Angiosperms	Poales	Poaceae	*Sorghum bicolor* (L.) Moench	4727.1	1172.0	1,3
8	Angiosperms	Poales	Poaceae	*Hakonechloa macra* (Munro) Makino	2056.0	372.0	1,3
9	Angiosperms	Poales	Poaceae	*Zea mays* L.	8279.9	1468.6	1,3
10	Angiosperms	Asparagales	Amaryllidaceae	*Allium sativum* L.	77053.3	29130.0	1,3
11	Angiosperms	Magnoliales	Magnoliaceae	*Liriodendron tulipifera* L.	4140.2	685.4	1,3
12	Angiosperms	Chloranthales	Chloranthaceae	*Chloranthus spicatus* (Thunb.) Makino	3658.7	212.4	1,3
13	Angiosperms	Austrobaileyales	Schisandraceae	*Schisandra chinensis* (Turcz.) Baill.	2611.5	1331.3	1,3
14	Angiosperms	Nymphaeales	Nymphaeaceae	*Nymphaea nouchali* Burm.f.	6986.2	859.0	1,3
15	Angiosperms	Amborellales	Amborellaceae	*Amborella trichopoda* Baill.	5421.6	1101.2	1,3
16	Gymnosperms	Gnetidae	Welwitschiaceae	*Welwitschia mirabilis* Hook.f.	2866.5	941.1	1,3
17	Gymnosperms	Pinales	Pinaceae	*Pinus peuce* Griseb.	892.3	573.6	2,5
18	Gymnosperms	Pinales	Pinaceae	*Pinus sylvestris* L.	986.5	117.1	1,3
19	Gymnosperms	Pinales	Pinaceae	*Pinus mugo* Turra	89.7	22.0	1,3
20	Gymnosperms	Pinales	Pinaceae	*Pinus cembra* L.	218.0	113.9	1,3
21	Gymnosperms	Pinales	Pinaceae	*Larix gmelinii* var. *principis-rupprechtii* (Mayr) Pilg.	79.6	6.6	1,3
22	Gymnosperms	Pinales	Pinaceae	*Picea abies* (L.) H.Karst.	13.6	3.1	1,3
23	Gymnosperms	Pinales	Pinaceae	*Abies numidica* de Lannoy ex Carrière	44.3	45.8	1,3
24	Gymnosperms	Pinales	Pinaceae	*Abies cephalonica* Loudon	18.0	5.6	1,3
25	Gymnosperms	Pinales	Pinaceae	*Abies koreana* E.H.Wilson	75.8	17.5	1,3
26	Gymnosperms	Cupressales/Pinales	Taxaceae	*Taxus baccata* L.	11.8	4.2	1,4
27	Gymnosperms	Cupressales	Cupressaceae	*Cryptomeria japonica* (Thunb. ex L.f.) D.Don	983.7	146.0	1,3
28	Gymnosperms	Cupressales	Cupressaceae	*Thujopsis dolabrata* (Thunb. ex L.f.) Sieb. & Zucc.	53.0	21.7	1,3
29	Gymnosperms	Araucariales	Araucariaceae	*Araucaria araucana* (Molina) K.Koch	521.7	240.2	2,5
30	Gymnosperms	Ginkgoales	Ginkgoaceae	*Ginkgo biloba* L.	1399.0	480.5	2,5
31	Gymnosperms	Cycadales	Cycadaceae	*Cycas revoluta* Thunb.	3604.2	3030.1	2,5
32	Monilophytes	Polypodiales	Polypodiaceae	*Polypodium vulgare* L.	94.0	79.8	2,5
33	Monilophytes	Polypodiales	Davalliaceae	*Davallia canariensis* (L.) Sm.	36.3	12.4	1,3
34	Monilophytes	Polypodiales	Tectariaceae	*Tectaria zeylanica* (Houtt.) Sledge	12.7	2.1	1,3
35	Monilophytes	Polypodiales	Dryopteridaceae	*Polystichum aculeatum* (L.) Roth ex Mert.	76.3	4.5	1,3
36	Monilophytes	Polypodiales	Onocleaceae	*Onoclea sensibilis* L.	87.7	10.4	1,3
37	Monilophytes	Polypodiales	Blechnaceae	*Blechnum spicant* (L.) Sm.	41.2	15.3	1,3
38	Monilophytes	Polypodiales	Thelypteridaceae	*Thelypteris palustri* Schott	45.7	13.8	1,3
39	Monilophytes	Polypodiales	Cystopteridaceae	*Gymnocarpium robertianum* (Hoffm.) Newman	66.2	16.8	1,3
40	Monilophytes	Polypodiales	Aspleniaceae	*Asplenium trichomanes* L.	178.1	12.0	1,3
41	Monilophytes	Polypodiales	Aspleniaceae	*Phyllitis scolopendrium* (L.) Newman	93.2	24.6	1,3
42	Monilophytes	Polypodiales	Pteridaceae	*Adiantum venustum* D. Don	59.2	2.1	1,3
43	Monilophytes	Cyatheales	Cyatheaceae	*Sphaeropteris cooperi* (F.Muell.) R.M.Tryon	122.7	24.1	1,3
44	Monilophytes	Salviniales	Salviniaceae	*Salvinia natans* (L.) All.	15.0	7.1	1,3
45	Monilophytes	Salviniales	Salviniaceae	*Salvinia molesta* D. S. Mitch.	862.4	708.2	2,5
46	Monilophytes	Schizaeales	Anemiaceae	*Anemia phyllitidis* (L.) Sw.	90.7	14.6	1,3
47	Monilophytes	Schizaeales	Lygodiaceae	*Lygodium volubile* Sw.	152.5	34.2	1,3
48	Monilophytes	Osmundales	Osmundaceae	*Osmunda regalis* L.	62.6	19.2	1,3
49	Monilophytes	Marattiales	Marattiaceae	*Angiopteris evecta* (G.Forst.) Hoffm.	29.6	9.1	1,3
50	Monilophytes	Equisetales	Equisetaceae	*Equisetum trachyodon* (A. Braun) W.D.J. Koch	57.8	33.3	1,3
51	Monilophytes	Psilotales	Psilotaceae	*Psilotum nudum* (L.) P. Beauv.	191.2	46.0	1,3
52	Lycophytes	Selaginellales	Selaginellaceae	*Selaginella moellendorffii* Hieron.	282.2	46.4	1,3
53	Lycophytes	Selaginellales	Selaginellaceae	*Selaginella pallescens* (C.Presl) Spring	768.9	514.2	2,6
54	Lycophytes	Lycopodiales	Lycopodiaceae	*Huperzia phlegmaria* (L.) Rothm.	124.1	49.3	1,3
55	Hornworts	Anthocerotales	Anthocerotaceae	*Anthoceros agrestis* Paton nom. cons. prop.	240.2	44.1	1,4
56	Hornworts	Anthocerotales	Anthocerotaceae	*Anthoceros punctatus* L.	165.4	24.9	1,3
57	Hornworts	Notothyladales	Notothyladaceae	*Phaeoceros laevis* (L.) Prosk.	887.2	261.4	1,3
58	Mosses	Funariales	Funariaceae	*Physcomitrella patens* (Hedw.) Bruch & Schimp.	491.6	567.5	3,6
59	Mosses	Funariales	Funariaceae	*Funaria hygrometrica* Hedw.	146.5	38.6	1,3
60	Mosses	Polytrichales	Polytrichaceae	*Polytrichum juniperinum* Hedw.	83.4	25.0	1,3
61	Mosses	Hedwigiales	Hedwigiaceae	*Hedwigia ciliata* (Hedw.) P.Beauv.	38.0	1.9	1,3
62	Mosses	Hypnales	Hylocomiaceae	*Hylocomium splendens* (Hedw.) Schimp. in B.S.G.	383.7	170.4	1,3
63	Liverworts	Marchantiales	Conocephalaceae	*Conocephalum conicum* (Linnaeus) Lindb.	420.2	161.2	1,3
64	Liverworts	Marchantiales	Marchantiaceae	*Marchantia polymorpha* L.	1650.9	524.0	1,3
65	Liverworts	Marchantiales	Ricciaceae	*Riccia fluitans* L.	1070.5	622.5	1,3
66	Algae	Charophytes	Characeae	*Chara vulgaris* Linnaeus	67.2	34.0	2,5
67	Algae	Charophytes	Klebsormidiaceae	*Klebsormidium elegans* Lokhorst	308.5	167.3	1,3
68	Algae	Chlorophyta	Caulerpaceae	*Caulerpa prolifera* (P. Forsskal) Lamouroux	32.4	28.2	1,3
69	Algae	Rhodophyta	Halymeniaceae	*Halymenia floresii* (Clemente y Rubio) C. Agardh	16.9	15.2	1,3
70	Lichen	Lecanorales	Parmeliaceae	*Cetraria islandica* (L.) Ach.	205.4	29.9	1,3
71	Lichen	Teloschistales	Teloschistaceae	*Xanthoria parietina* (L.) Th. Fr.	1872.4	144.4	1,3

Supplementary Table S6: Concentrations of oleoyl ethanolamide or OEA (pmol/g plant weight) found in the 71 plant species analyzed. Values are reported as the mean (X) and standard deviation (SD) of at least triplicates (n) of at least one plant species (N).

#	Group	Order	Family	Species	OEA (pmol/g PW)		N,n
					X	SD	
1	Angiosperms	Santalales	Viscaceae	*Viscum album* L.	997.9	247.6	1,3
2	Angiosperms	Brassicales	Brassicaceae	*Arabidopsis thaliana* (L.) Heynh.	284.2	211.4	2,5
3	Angiosperms	Malvales	Malvaceae	*Theobroma cacao* L.	93.9	2.3	1,3
4	Angiosperms	Rosales	Cannabaceae	*Cannabis sativa* L.	15.9	3.1	2,5
5	Angiosperms	Malpighiales	Rhizophoraceae	*Rhizophora mangle* L.	101.9	24.5	1,3
6	Angiosperms	Malpighiales	Salicaceae	*Salix glabra* Scop.	88.1	22.8	1,3
7	Angiosperms	Poales	Poaceae	*Sorghum bicolor* (L.) Moench	160.0	26.1	1,3
8	Angiosperms	Poales	Poaceae	*Hakonechloa macra* (Munro) Makino	213.9	42.0	1,3
9	Angiosperms	Poales	Poaceae	*Zea mays* L.	228.3	42.4	1,3
10	Angiosperms	Asparagales	Amaryllidaceae	*Allium sativum* L.	1382.4	534.9	1,3
11	Angiosperms	Magnoliales	Magnoliaceae	*Liriodendron tulipifera* L.	211.6	17.1	1,3
12	Angiosperms	Chloranthales	Chloranthaceae	*Chloranthus spicatus* (Thunb.) Makino	121.7	5.8	1,3
13	Angiosperms	Austrobaileyales	Schisandraceae	*Schisandra chinensis* (Turcz.) Baill.	64.3	38.1	1,3
14	Angiosperms	Nymphaeales	Nymphaeaceae	*Nymphaea nouchali* Burm.f.	200.0	32.0	1,3
15	Angiosperms	Amborellales	Amborellaceae	*Amborella trichopoda* Baill.	261.0	40.5	1,3
16	Gymnosperms	Gnetidae	Welwitschiaceae	*Welwitschia mirabilis* Hook.f.	2681.4	1003.0	1,3
17	Gymnosperms	Pinales	Pinaceae	*Pinus peuce* Griseb.	131.2	108.2	2,5
18	Gymnosperms	Pinales	Pinaceae	*Pinus sylvestris* L.	74.3	4.3	1,3
19	Gymnosperms	Pinales	Pinaceae	*Pinus mugo* Turra	29.9	2.6	1,3
20	Gymnosperms	Pinales	Pinaceae	*Pinus cembra* L.	12.6	5.3	1,3
21	Gymnosperms	Pinales	Pinaceae	*Larix gmelinii* var. *principis-rupprechtii* (Mayr) Pilg.	12.8	0.8	1,3
22	Gymnosperms	Pinales	Pinaceae	*Picea abies* (L.) H.Karst.	1.8	0.1	1,3
23	Gymnosperms	Pinales	Pinaceae	*Abies numidica* de Lannoy ex Carrière	18.7	15.3	1,3
24	Gymnosperms	Pinales	Pinaceae	*Abies cephalonica* Loudon	4.3	1.8	1,3
25	Gymnosperms	Pinales	Pinaceae	*Abies koreana* E.H.Wilson	32.0	0.7	1,3
26	Gymnosperms	Cupressales/Pinales	Taxaceae	*Taxus baccata* L.	4.6	1.3	1,4
27	Gymnosperms	Cupressales	Cupressaceae	*Cryptomeria japonica* (Thunb. ex L.f.) D.Don	56.6	5.1	1,3
28	Gymnosperms	Cupressales	Cupressaceae	*Thujopsis dolabrata* (Thunb. ex L.f.) Sieb. & Zucc.	5.6	3.0	1,3
29	Gymnosperms	Araucariales	Araucariaceae	*Araucaria araucana* (Molina) K.Koch	93.7	56.5	2,5
30	Gymnosperms	Ginkgoales	Ginkgoaceae	*Ginkgo biloba* L.	755.1	359.7	2,5
31	Gymnosperms	Cycadales	Cycadaceae	*Cycas revoluta* Thunb.	424.6	347.4	2,5
32	Monilophytes	Polypodiales	Polypodiaceae	*Polypodium vulgare* L.	15.1	4.8	2,5
33	Monilophytes	Polypodiales	Davalliaceae	*Davallia canariensis* (L.) Sm.	3.7	0.2	1,3
34	Monilophytes	Polypodiales	Tectariaceae	*Tectaria zeylanica* (Houtt.) Sledge	1.8	0.4	1,3
35	Monilophytes	Polypodiales	Dryopteridaceae	*Polystichum aculeatum* (L.) Roth ex Mert.	24.9	6.8	1,3
36	Monilophytes	Polypodiales	Onocleaceae	*Onoclea sensibilis* L.	9.1	1.4	1,3
37	Monilophytes	Polypodiales	Blechnaceae	*Blechnum spicant* (L.) Sm.	3.8	1.3	1,3
38	Monilophytes	Polypodiales	Thelypteridaceae	*Thelypteris palustri* Schott	2.3	2.1	1,3
39	Monilophytes	Polypodiales	Cystopteridaceae	*Gymnocarpium robertianum* (Hoffm.) Newman	8.7	3.5	1,3
40	Monilophytes	Polypodiales	Aspleniaceae	*Asplenium trichomanes* L.	20.6	1.3	1,3
41	Monilophytes	Polypodiales	Aspleniaceae	*Phyllitis scolopendrium* (L.) Newman	9.6	1.6	1,3
42	Monilophytes	Polypodiales	Pteridaceae	*Adiantum venustum* D. Don	7.6	0.3	1,3
43	Monilophytes	Cyatheales	Cyatheaceae	*Sphaeropteris cooperi* (F.Muell.) R.M.Tryon	10.0	1.4	1,3
44	Monilophytes	Salviniales	Salviniaceae	*Salvinia natans* (L.) All.	4.4	2.3	1,3
45	Monilophytes	Salviniales	Salviniaceae	*Salvinia molesta* D. S. Mitch.	104.0	73.9	2,5
46	Monilophytes	Schizaeales	Anemiaceae	*Anemia phyllitidis* (L.) Sw.	5.0	1.2	1,3
47	Monilophytes	Schizaeales	Lygodiaceae	*Lygodium volubile* Sw.	55.0	10.9	1,3
48	Monilophytes	Osmundales	Osmundaceae	*Osmunda regalis* L.	6.7	2.2	1,3
49	Monilophytes	Marattiales	Marattiaceae	*Angiopteris evecta* (G.Forst.) Hoffm.	2.1	0.5	1,3
50	Monilophytes	Equisetales	Equisetaceae	*Equisetum trachyodon* (A. Braun) W.D.J. Koch	2.4	1.3	1,3
51	Monilophytes	Psilotales	Psilotaceae	*Psilotum nudum* (L.) P. Beauv.	13.2	2.7	1,3
52	Lycophytes	Selaginellales	Selaginellaceae	*Selaginella moellendorffii* Hieron.	16.5	2.3	1,3
53	Lycophytes	Selaginellales	Selaginellaceae	*Selaginella pallescens* (C.Presl) Spring	33.7	13.0	2,6
54	Lycophytes	Lycopodiales	Lycopodiaceae	*Huperzia phlegmaria* (L.) Rothm.	17.0	8.0	1,3
55	Hornworts	Anthocerotales	Anthocerotaceae	*Anthoceros agrestis* Paton nom. cons. prop.	41.4	11.5	1,4
56	Hornworts	Anthocerotales	Anthocerotaceae	*Anthoceros punctatus* L.	148.3	23.1	1,3
57	Hornworts	Notothyladales	Notothyladaceae	*Phaeoceros laevis* (L.) Prosk.	126.8	37.4	1,3
58	Mosses	Funariales	Funariaceae	*Physcomitrella patens* (Hedw.) Bruch & Schimp.	132.7	108.5	3,6
59	Mosses	Funariales	Funariaceae	*Funaria hygrometrica* Hedw.	21.2	5.9	1,3
60	Mosses	Polytrichales	Polytrichaceae	*Polytrichum juniperinum* Hedw.	9.0	4.0	1,3
61	Mosses	Hedwigiales	Hedwigiaceae	*Hedwigia ciliata* (Hedw.) P.Beauv.	7.0	1.9	1,3
62	Mosses	Hypnales	Hylocomiaceae	*Hylocomium splendens* (Hedw.) Schimp. in B.S.G.	17.2	2.5	1,3
63	Liverworts	Marchantiales	Conocephalaceae	*Conocephalum conicum* (Linnaeus) Lindb.	59.4	16.4	1,3
64	Liverworts	Marchantiales	Marchantiaceae	*Marchantia polymorpha* L.	258.0	66.3	1,3
65	Liverworts	Marchantiales	Ricciaceae	*Riccia fluitans* L.	1147.7	560.5	1,3
66	Algae	Charophytes	Characeae	*Chara vulgaris* Linnaeus	17.5	6.7	2,5
67	Algae	Charophytes	Klebsormidiaceae	*Klebsormidium elegans* Lokhorst	261.0	44.6	1,3
68	Algae	Chlorophyta	Caulerpaceae	*Caulerpa prolifera* (P. Forsskal) Lamouroux	11.8	0.1	1,3
69	Algae	Rhodophyta	Halymeniaceae	*Halymenia floresii* (Clemente y Rubio) C. Agardh	14.0	3.0	1,3
70	Lichen	Lecanorales	Parmeliaceae	*Cetraria islandica* (L.) Ach.	40.8	6.6	1,3
71	Lichen	Teloschistales	Teloschistaceae	*Xanthoria parietina* (L.) Th. Fr.	71.9	11.0	1,3

Supplementary Table S7: Concentrations of palmitoyl ethanolamide or PEA (pmol/g plant weight) found in the 71 plant species analyzed. Values are reported as the mean (X) and standard deviation (SD) of at least triplicates (n) of at least one plant species (N).

#	Group	Order	Family	Species	PEA (pmol/g PW)		N,n
					X	SD	
1	Angiosperms	Santalales	Viscaceae	*Viscum album* L.	1420.0	313.2	1,3
2	Angiosperms	Brassicales	Brassicaceae	*Arabidopsis thaliana* (L.) Heynh.	3588.6	2854.1	2,5
3	Angiosperms	Malvales	Malvaceae	*Theobroma cacao* L.	306.7	43.8	1,3
4	Angiosperms	Rosales	Cannabaceae	*Cannabis sativa* L.	127.5	22.0	2,5
5	Angiosperms	Malpighiales	Rhizophoraceae	*Rhizophora mangle* L.	57.0	9.2	1,3
6	Angiosperms	Malpighiales	Salicaceae	*Salix glabra* Scop.	630.9	148.6	1,3
7	Angiosperms	Poales	Poaceae	*Sorghum bicolor* (L.) Moench	977.5	79.7	1,3
8	Angiosperms	Poales	Poaceae	*Hakonechloa macra* (Munro) Makino	254.1	32.6	1,3
9	Angiosperms	Poales	Poaceae	*Zea mays* L.	1360.5	237.5	1,3
10	Angiosperms	Asparagales	Amaryllidaceae	*Allium sativum* L.	16387.9	6479.0	1,3
11	Angiosperms	Magnoliales	Magnoliaceae	*Liriodendron tulipifera* L.	594.8	61.8	1,3
12	Angiosperms	Chloranthales	Chloranthaceae	*Chloranthus spicatus* (Thunb.) Makino	212.9	7.6	1,3
13	Angiosperms	Austrobaileyales	Schisandraceae	*Schisandra chinensis* (Turcz.) Baill.	675.1	347.9	1,3
14	Angiosperms	Nymphaeales	Nymphaeaceae	*Nymphaea nouchali* Burm.f.	2746.3	381.5	1,3
15	Angiosperms	Amborellales	Amborellaceae	*Amborella trichopoda* Baill.	1510.0	58.7	1,3
16	Gymnosperms	Gnetidae	Welwitschiaceae	*Welwitschia mirabilis* Hook.f.	1653.2	568.7	1,3
17	Gymnosperms	Pinales	Pinaceae	*Pinus peuce* Griseb.	278.2	218.8	2,5
18	Gymnosperms	Pinales	Pinaceae	*Pinus sylvestris* L.	155.7	8.8	1,3
19	Gymnosperms	Pinales	Pinaceae	*Pinus mugo* Turra	40.0	2.1	1,3
20	Gymnosperms	Pinales	Pinaceae	*Pinus cembra* L.	27.7	5.3	1,3
21	Gymnosperms	Pinales	Pinaceae	*Larix gmelinii* var. *principis-rupprechtii* (Mayr) Pilg.	20.9	3.1	1,3
22	Gymnosperms	Pinales	Pinaceae	*Picea abies* (L.) H.Karst.	7.2	0.5	1,3
23	Gymnosperms	Pinales	Pinaceae	*Abies numidica* de Lannoy ex Carrière	<LOD	-	1,3
24	Gymnosperms	Pinales	Pinaceae	*Abies cephalonica* Loudon	<LOD	-	1,3
25	Gymnosperms	Pinales	Pinaceae	*Abies koreana* E.H.Wilson	<LOD	-	1,3
26	Gymnosperms	Cupressales/Pinales	Taxaceae	*Taxus baccata* L.	769.3	335.1	1,4
27	Gymnosperms	Cupressales	Cupressaceae	*Cryptomeria japonica* (Thunb. ex L.f.) D.Don	107.5	21.4	1,3
28	Gymnosperms	Cupressales	Cupressaceae	*Thujopsis dolabrata* (Thunb. ex L.f.) Sieb. & Zucc.	58.2	31.1	1,3
29	Gymnosperms	Araucariales	Araucariaceae	*Araucaria araucana* (Molina) K.Koch	182.5	85.5	2,5
30	Gymnosperms	Ginkgoales	Ginkgoaceae	*Ginkgo biloba* L.	651.1	158.1	2,5
31	Gymnosperms	Cycadales	Cycadaceae	*Cycas revoluta* Thunb.	298.1	243.1	2,5
32	Monilophytes	Polypodiales	Polypodiaceae	*Polypodium vulgare* L.	1140.2	834.6	2,5
33	Monilophytes	Polypodiales	Davalliaceae	*Davallia canariensis* (L.) Sm.	629.4	1000.7	1,3
34	Monilophytes	Polypodiales	Tectariaceae	*Tectaria zeylanica* (Houtt.) Sledge	19.2	5.4	1,3
35	Monilophytes	Polypodiales	Dryopteridaceae	*Polystichum aculeatum* (L.) Roth ex Mert.	1969.2	531.9	1,3
36	Monilophytes	Polypodiales	Onocleaceae	*Onoclea sensibilis* L.	268.0	362.2	1,3
37	Monilophytes	Polypodiales	Blechnaceae	*Blechnum spicant* (L.) Sm.	59.2	7.5	1,3
38	Monilophytes	Polypodiales	Thelypteridaceae	*Thelypteris palustri* Schott	425.1	712.3	1,3
39	Monilophytes	Polypodiales	Cystopteridaceae	*Gymnocarpium robertianum* (Hoffm.) Newman	1291.1	686.7	1,3
40	Monilophytes	Polypodiales	Aspleniaceae	*Asplenium trichomanes* L.	1095.0	107.5	1,3
41	Monilophytes	Polypodiales	Aspleniaceae	*Phyllitis scolopendrium* (L.) Newman	482.8	97.2	1,3
42	Monilophytes	Polypodiales	Pteridaceae	*Adiantum venustum* D. Don	40.2	3.5	1,3
43	Monilophytes	Cyatheales	Cyatheaceae	*Sphaeropteris cooperi* (F.Muell.) R.M.Tryon	368.8	63.1	1,3
44	Monilophytes	Salviniales	Salviniaceae	*Salvinia natans* (L.) All.	1407.3	666.5	1,3
45	Monilophytes	Salviniales	Salviniaceae	*Salvinia molesta* D. S. Mitch.	445.2	301.5	2,5
46	Monilophytes	Schizaeales	Anemiaceae	*Anemia phyllitidis* (L.) Sw.	28.8	8.6	1,3
47	Monilophytes	Schizaeales	Lygodiaceae	*Lygodium volubile* Sw.	2481.3	289.8	1,3
48	Monilophytes	Osmundales	Osmundaceae	*Osmunda regalis* L.	58.3	72.4	1,3
49	Monilophytes	Marattiales	Marattiaceae	*Angiopteris evecta* (G.Forst.) Hoffm.	9.5	1.7	1,3
50	Monilophytes	Equisetales	Equisetaceae	*Equisetum trachyodon* (A. Braun) W.D.J. Koch	91.9	66.5	1,3
51	Monilophytes	Psilotales	Psilotaceae	*Psilotum nudum* (L.) P. Beauv.	258.9	196.9	1,3
52	Lycophytes	Selaginellales	Selaginellaceae	*Selaginella moellendorffii* Hieron.	80.2	10.1	1,3
53	Lycophytes	Selaginellales	Selaginellaceae	*Selaginella pallescens* (C.Presl) Spring	176.1	58.7	2,6
54	Lycophytes	Lycopodiales	Lycopodiaceae	*Huperzia phlegmaria* (L.) Rothm.	89.3	136.3	1,3
55	Hornworts	Anthocerotales	Anthocerotaceae	*Anthoceros agrestis* Paton nom. cons. prop.	258.1	24.7	1,4
56	Hornworts	Anthocerotales	Anthocerotaceae	*Anthoceros punctatus* L.	699.9	148.3	1,3
57	Hornworts	Notothyladales	Notothyladaceae	*Phaeoceros laevis* (L.) Prosk.	387.7	71.9	1,3
58	Mosses	Funariales	Funariaceae	*Physcomitrella patens* (Hedw.) Bruch & Schimp.	563.6	419.7	3,6
59	Mosses	Funariales	Funariaceae	*Funaria hygrometrica* Hedw.	69.0	14.0	1,3
60	Mosses	Polytrichales	Polytrichaceae	*Polytrichum juniperinum* Hedw.	63.1	33.2	1,3
61	Mosses	Hedwigiales	Hedwigiaceae	*Hedwigia ciliata* (Hedw.) P.Beauv.	47.7	42.3	1,3
62	Mosses	Hypnales	Hylocomiaceae	*Hylocomium splendens* (Hedw.) Schimp. in B.S.G.	341.6	36.3	1,3
63	Liverworts	Marchantiales	Conocephalaceae	*Conocephalum conicum* (Linnaeus) Lindb.	244.2	62.8	1,3
64	Liverworts	Marchantiales	Marchantiaceae	*Marchantia polymorpha* L.	591.3	223.1	1,3
65	Liverworts	Marchantiales	Ricciaceae	*Riccia fluitans* L.	2463.7	1456.7	1,3
66	Algae	Charophytes	Characeae	*Chara vulgaris* Linnaeus	101.4	16.6	2,5
67	Algae	Charophytes	Klebsormidiaceae	*Klebsormidium elegans* Lokhorst	1070.9	85.6	1,3
68	Algae	Chlorophyta	Caulerpaceae	*Caulerpa prolifera* (P. Forsskal) Lamouroux	173.8	166.7	1,3
69	Algae	Rhodophyta	Halymeniaceae	*Halymenia floresii* (Clemente y Rubio) C. Agardh	94.8	8.7	1,3
70	Lichen	Lecanorales	Parmeliaceae	*Cetraria islandica* (L.) Ach.	72.7	15.0	1,3
71	Lichen	Teloschistales	Teloschistaceae	*Xanthoria parietina* (L.) Th. Fr.	183.5	40.6	1,3

Supplementary Table S8: Concentrations of stearoyl ethanolamide or SEA (pmol/g plant weight) found in the 71 plant species analyzed. Values are reported as the mean (X) and standard deviation (SD) of at least triplicates (n) of at least one plant species (N).

#	Group	Order	Family	Species	SEA (pmol/g PW)		N,n
					X	SD	
1	Angiosperms	Santalales	Viscaceae	*Viscum album* L.	96.9	11.2	1,3
2	Angiosperms	Brassicales	Brassicaceae	*Arabidopsis thaliana* (L.) Heynh.	212.4	37.0	2,5
3	Angiosperms	Malvales	Malvaceae	*Theobroma cacao* L.	40.8	9.5	1,3
4	Angiosperms	Rosales	Cannabaceae	*Cannabis sativa* L.	98.1	54.3	2,5
5	Angiosperms	Malpighiales	Rhizophoraceae	*Rhizophora mangle* L.	67.8	25.1	1,3
6	Angiosperms	Malpighiales	Salicaceae	*Salix glabra* Scop.	<LOD	-	1,3
7	Angiosperms	Poales	Poaceae	*Sorghum bicolor* (L.) Moench	105.3	30.9	1,3
8	Angiosperms	Poales	Poaceae	*Hakonechloa macra* (Munro) Makino	<LOD	-	1,3
9	Angiosperms	Poales	Poaceae	*Zea mays* L.	142.2	48.1	1,3
10	Angiosperms	Asparagales	Amaryllidaceae	*Allium sativum* L.	1419.9	716.3	1,3
11	Angiosperms	Magnoliales	Magnoliaceae	*Liriodendron tulipifera* L.	123.8	21.0	1,3
12	Angiosperms	Chloranthales	Chloranthaceae	*Chloranthus spicatus* (Thunb.) Makino	65.2	26.5	1,3
13	Angiosperms	Austrobaileyales	Schisandraceae	*Schisandra chinensis* (Turcz.) Baill.	86.2	11.0	1,3
14	Angiosperms	Nymphaeales	Nymphaeaceae	*Nymphaea nouchali* Burm.f.	147.5	51.8	1,3
15	Angiosperms	Amborellales	Amborellaceae	*Amborella trichopoda* Baill.	157.2	81.2	1,3
16	Gymnosperms	Gnetidae	Welwitschiaceae	*Welwitschia mirabilis* Hook.f.	879.6	114.4	1,3
17	Gymnosperms	Pinales	Pinaceae	*Pinus peuce* Griseb.	71.3	38.7	2,5
18	Gymnosperms	Pinales	Pinaceae	*Pinus sylvestris* L.	37.3	3.9	1,3
19	Gymnosperms	Pinales	Pinaceae	*Pinus mugo* Turra	<LOD	-	1,3
20	Gymnosperms	Pinales	Pinaceae	*Pinus cembra* L.	23.3	1.5	1,3
21	Gymnosperms	Pinales	Pinaceae	*Larix gmelinii* var. *principis-rupprechtii* (Mayr) Pilg.	23.8	5.1	1,3
22	Gymnosperms	Pinales	Pinaceae	*Picea abies* (L.) H.Karst.	9.2	0.4	1,3
23	Gymnosperms	Pinales	Pinaceae	*Abies numidica* de Lannoy ex Carrière	<LOD	-	1,3
24	Gymnosperms	Pinales	Pinaceae	*Abies cephalonica* Loudon	<LOD	-	1,3
25	Gymnosperms	Pinales	Pinaceae	*Abies koreana* E.H.Wilson	<LOD	-	1,3
26	Gymnosperms	Cupressales/Pinales	Taxaceae	*Taxus baccata* L.	6057.0	2384.8	1,4
27	Gymnosperms	Cupressales	Cupressaceae	*Cryptomeria japonica* (Thunb. ex L.f.) D.Don	<LOD	-	1,3
28	Gymnosperms	Cupressales	Cupressaceae	*Thujopsis dolabrata* (Thunb. ex L.f.) Sieb. & Zucc.	<LOD	-	1,3
29	Gymnosperms	Araucariales	Araucariaceae	*Araucaria araucana* (Molina) K.Koch	<LOD	-	2,5
30	Gymnosperms	Ginkgoales	Ginkgoaceae	*Ginkgo biloba* L.	76.3	33.1	2,5
31	Gymnosperms	Cycadales	Cycadaceae	*Cycas revoluta* Thunb.	83.2	86.8	2,5
32	Monilophytes	Polypodiales	Polypodiaceae	*Polypodium vulgare* L.	7722.1	2110.7	2,5
33	Monilophytes	Polypodiales	Davalliaceae	*Davallia canariensis* (L.) Sm.	7072.4	12081.1	1,3
34	Monilophytes	Polypodiales	Tectariaceae	*Tectaria zeylanica* (Houtt.) Sledge	54.1	32.8	1,3
35	Monilophytes	Polypodiales	Dryopteridaceae	*Polystichum aculeatum* (L.) Roth ex Mert.	3443.5	435.8	1,3
36	Monilophytes	Polypodiales	Onocleaceae	*Onoclea sensibilis* L.	2053.0	3249.7	1,3
37	Monilophytes	Polypodiales	Blechnaceae	*Blechnum spicant* (L.) Sm.	350.9	258.1	1,3
38	Monilophytes	Polypodiales	Thelypteridaceae	*Thelypteris palustri* Schott	<LOD	-	1,3
39	Monilophytes	Polypodiales	Cystopteridaceae	*Gymnocarpium robertianum* (Hoffm.) Newman	6262.4	1217.4	1,3
40	Monilophytes	Polypodiales	Aspleniaceae	*Asplenium trichomanes* L.	4379.2	1894.7	1,3
41	Monilophytes	Polypodiales	Aspleniaceae	*Phyllitis scolopendrium* (L.) Newman	1945.2	700.0	1,3
42	Monilophytes	Polypodiales	Pteridaceae	*Adiantum venustum* D. Don	<LOD	-	1,3
43	Monilophytes	Cyatheales	Cyatheaceae	*Sphaeropteris cooperi* (F.Muell.) R.M.Tryon	2054.9	1148.8	1,3
44	Monilophytes	Salviniales	Salviniaceae	*Salvinia natans* (L.) All.	3748.3	1396.5	1,3
45	Monilophytes	Salviniales	Salviniaceae	*Salvinia molesta* D. S. Mitch.	179.8	112.3	2,5
46	Monilophytes	Schizaeales	Anemiaceae	*Anemia phyllitidis* (L.) Sw.	131.1	119.2	1,3
47	Monilophytes	Schizaeales	Lygodiaceae	*Lygodium volubile* Sw.	8170.9	988.3	1,3
48	Monilophytes	Osmundales	Osmundaceae	*Osmunda regalis* L.	35.3	23.3	1,3
49	Monilophytes	Marattiales	Marattiaceae	*Angiopteris evecta* (G.Forst.) Hoffm.	<LOD	-	1,3
50	Monilophytes	Equisetales	Equisetaceae	*Equisetum trachyodon* (A. Braun) W.D.J. Koch	81.7	15.9	1,3
51	Monilophytes	Psilotales	Psilotaceae	*Psilotum nudum* (L.) P. Beauv.	192.2	88.7	1,3
52	Lycophytes	Selaginellales	Selaginellaceae	*Selaginella moellendorffii* Hieron.	<LOD	-	1,3
53	Lycophytes	Selaginellales	Selaginellaceae	*Selaginella pallescens* (C.Presl) Spring	179.1	79.9	2,6
54	Lycophytes	Lycopodiales	Lycopodiaceae	*Huperzia phlegmaria* (L.) Rothm.	<LOD	-	1,3
55	Hornworts	Anthocerotales	Anthocerotaceae	*Anthoceros agrestis* Paton nom. cons. prop.	130.4	41.1	1,4
56	Hornworts	Anthocerotales	Anthocerotaceae	*Anthoceros punctatus* L.	<LOD	-	1,3
57	Hornworts	Notothyladales	Notothyladaceae	*Phaeoceros laevis* (L.) Prosk.	<LOD	-	1,3
58	Mosses	Funariales	Funariaceae	*Physcomitrella patens* (Hedw.) Bruch & Schimp.	<LOD	-	3,6
59	Mosses	Funariales	Funariaceae	*Funaria hygrometrica* Hedw.	<LOD	-	1,3
60	Mosses	Polytrichales	Polytrichaceae	*Polytrichum juniperinum* Hedw.	191.2	170.7	1,3
61	Mosses	Hedwigiales	Hedwigiaceae	*Hedwigia ciliata* (Hedw.) P.Beauv.	<LOD	-	1,3
62	Mosses	Hypnales	Hylocomiaceae	*Hylocomium splendens* (Hedw.) Schimp. in B.S.G.	48.1	33.4	1,3
63	Liverworts	Marchantiales	Conocephalaceae	*Conocephalum conicum* (Linnaeus) Lindb.	<LOD	-	1,3
64	Liverworts	Marchantiales	Marchantiaceae	*Marchantia polymorpha* L.	150.7	60.0	1,3
65	Liverworts	Marchantiales	Ricciaceae	*Riccia fluitans* L.	538.5	386.4	1,3
66	Algae	Charophytes	Characeae	*Chara vulgaris* Linnaeus	<LOD	-	2,5
67	Algae	Charophytes	Klebsormidiaceae	*Klebsormidium elegans* Lokhorst	1093.0	767.1	1,3
68	Algae	Chlorophyta	Caulerpaceae	*Caulerpa prolifera* (P. Forsskal) Lamouroux	<LOD	-	1,3
69	Algae	Rhodophyta	Halymeniaceae	*Halymenia floresii* (Clemente y Rubio) C. Agardh	<LOD	-	1,3
70	Lichen	Lecanorales	Parmeliaceae	*Cetraria islandica* (L.) Ach.	<LOD	-	1,3
71	Lichen	Teloschistales	Teloschistaceae	*Xanthoria parietina* (L.) Th. Fr.	216.4	175.9	1,3

Supplementary Table S9: Concentrations of stearic acid or STE (18:0) (pmol/g plant weight) found in the 71 plant species analyzed. Values are reported as the mean (X) and standard deviation (SD) of at least triplicates (n) of at least one plant species (N).

#	Group	Order	Family	Species	STE (pmol/g PW)		N,n
					X	SD	
1	Angiosperms	Santalales	Viscaceae	*Viscum album* L.	1793985.8	482275.9	1,3
2	Angiosperms	Brassicales	Brassicaceae	*Arabidopsis thaliana* (L.) Heynh.	710665.7	377957.2	2,5
3	Angiosperms	Malvales	Malvaceae	*Theobroma cacao* L.	52498.4	45006.7	1,3
4	Angiosperms	Rosales	Cannabaceae	*Cannabis sativa* L.	120666.4	52322.8	2,5
5	Angiosperms	Malpighiales	Rhizophoraceae	*Rhizophora mangle* L.	110796.7	31610.0	1,3
6	Angiosperms	Malpighiales	Salicaceae	*Salix glabra* Scop.	575567.6	91177.7	1,3
7	Angiosperms	Poales	Poaceae	*Sorghum bicolor* (L.) Moench	446422.6	55271.0	1,3
8	Angiosperms	Poales	Poaceae	*Hakonechloa macra* (Munro) Makino	273283.0	31832.7	1,3
9	Angiosperms	Poales	Poaceae	*Zea mays* L.	1286745.2	156574.9	1,3
10	Angiosperms	Asparagales	Amaryllidaceae	*Allium sativum* L.	988868.6	377392.5	1,3
11	Angiosperms	Magnoliales	Magnoliaceae	*Liriodendron tulipifera* L.	552722.3	36558.3	1,3
12	Angiosperms	Chloranthales	Chloranthaceae	*Chloranthus spicatus* (Thunb.) Makino	319188.7	43020.8	1,3
13	Angiosperms	Austrobaileyales	Schisandraceae	*Schisandra chinensis* (Turcz.) Baill.	800284.3	720103.8	1,3
14	Angiosperms	Nymphaeales	Nymphaeaceae	*Nymphaea nouchali* Burm.f.	1000273.1	641477.1	1,3
15	Angiosperms	Amborellales	Amborellaceae	*Amborella trichopoda* Baill.	420899.3	74800.3	1,3
16	Gymnosperms	Gnetidae	Welwitschiaceae	*Welwitschia mirabilis* Hook.f.	545012.0	233303.4	1,3
17	Gymnosperms	Pinales	Pinaceae	*Pinus peuce* Griseb.	863726.7	565622.0	2,5
18	Gymnosperms	Pinales	Pinaceae	*Pinus sylvestris* L.	171460.5	143403.5	1,3
19	Gymnosperms	Pinales	Pinaceae	*Pinus mugo* Turra	105228.9	81578.1	1,3
20	Gymnosperms	Pinales	Pinaceae	*Pinus cembra* L.	98371.6	82259.2	1,3
21	Gymnosperms	Pinales	Pinaceae	*Larix gmelinii* var. *principis-rupprechtii* (Mayr) Pilg.	162640.8	149206.7	1,3
22	Gymnosperms	Pinales	Pinaceae	*Picea abies* (L.) H.Karst.	40322.3	34520.1	1,3
23	Gymnosperms	Pinales	Pinaceae	*Abies numidica* de Lannoy ex Carrière	58885.0	52062.5	1,3
24	Gymnosperms	Pinales	Pinaceae	*Abies cephalonica* Loudon	121607.9	110409.7	1,3
25	Gymnosperms	Pinales	Pinaceae	*Abies koreana* E.H.Wilson	97141.6	28329.7	1,3
26	Gymnosperms	Cupressales/Pinales	Taxaceae	*Taxus baccata* L.	53896.6	50210.6	1,4
27	Gymnosperms	Cupressales	Cupressaceae	*Cryptomeria japonica* (Thunb. ex L.f.) D.Don	546965.6	161613.9	1,3
28	Gymnosperms	Cupressales	Cupressaceae	*Thujopsis dolabrata* (Thunb. ex L.f.) Sieb. & Zucc.	194562.6	71609.5	1,3
29	Gymnosperms	Araucariales	Araucariaceae	*Araucaria araucana* (Molina) K.Koch	400852.4	297923.1	2,5
30	Gymnosperms	Ginkgoales	Ginkgoaceae	*Ginkgo biloba* L.	6173280.7	7161360.6	2,5
31	Gymnosperms	Cycadales	Cycadaceae	*Cycas revoluta* Thunb.	549507.1	442187.1	2,5
32	Monilophytes	Polypodiales	Polypodiaceae	*Polypodium vulgare* L.	171579.6	32698.5	2,5
33	Monilophytes	Polypodiales	Davalliaceae	*Davallia canariensis* (L.) Sm.	234286.1	175063.9	1,3
34	Monilophytes	Polypodiales	Tectariaceae	*Tectaria zeylanica* (Houtt.) Sledge	71072.3	35736.8	1,3
35	Monilophytes	Polypodiales	Dryopteridaceae	*Polystichum aculeatum* (L.) Roth ex Mert.	144908.4	38697.3	1,3
36	Monilophytes	Polypodiales	Onocleaceae	*Onoclea sensibilis* L.	133466.3	6455.3	1,3
37	Monilophytes	Polypodiales	Blechnaceae	*Blechnum spicant* (L.) Sm.	57955.2	9923.7	1,3
38	Monilophytes	Polypodiales	Thelypteridaceae	*Thelypteris palustri* Schott	89662.0	15056.1	1,3
39	Monilophytes	Polypodiales	Cystopteridaceae	*Gymnocarpium robertianum* (Hoffm.) Newman	154575.2	37828.7	1,3
40	Monilophytes	Polypodiales	Aspleniaceae	*Asplenium trichomanes* L.	148684.8	40518.0	1,3
41	Monilophytes	Polypodiales	Aspleniaceae	*Phyllitis scolopendrium* (L.) Newman	76459.4	13134.0	1,3
42	Monilophytes	Polypodiales	Pteridaceae	*Adiantum venustum* D. Don	210498.2	70583.3	1,3
43	Monilophytes	Cyatheales	Cyatheaceae	*Sphaeropteris cooperi* (F.Muell.) R.M.Tryon	138391.7	115738.1	1,3
44	Monilophytes	Salviniales	Salviniaceae	*Salvinia natans* (L.) All.	26130.8	13331.9	1,3
45	Monilophytes	Salviniales	Salviniaceae	*Salvinia molesta* D. S. Mitch.	430715.0	212095.1	2,5
46	Monilophytes	Schizaeales	Anemiaceae	*Anemia phyllitidis* (L.) Sw.	63336.3	7850.9	1,3
47	Monilophytes	Schizaeales	Lygodiaceae	*Lygodium volubile* Sw.	10819008.8	9674626.6	1,3
48	Monilophytes	Osmundales	Osmundaceae	*Osmunda regalis* L.	71277.2	5853.0	1,3
49	Monilophytes	Marattiales	Marattiaceae	*Angiopteris evecta* (G.Forst.) Hoffm.	42068.9	7616.7	1,3
50	Monilophytes	Equisetales	Equisetaceae	*Equisetum trachyodon* (A. Braun) W.D.J. Koch	147520.5	72202.2	1,3
51	Monilophytes	Psilotales	Psilotaceae	*Psilotum nudum* (L.) P. Beauv.	211052.2	3258.0	1,3
52	Lycophytes	Selaginellales	Selaginellaceae	*Selaginella moellendorffii* Hieron.	249766.6	46935.1	1,3
53	Lycophytes	Selaginellales	Selaginellaceae	*Selaginella pallescens* (C.Presl) Spring	294576.8	118758.2	2,6
54	Lycophytes	Lycopodiales	Lycopodiaceae	*Huperzia phlegmaria* (L.) Rothm.	395542.9	259726.7	1,3
55	Hornworts	Anthocerotales	Anthocerotaceae	*Anthoceros agrestis* Paton nom. cons. prop.	230231.6	95506.6	1,4
56	Hornworts	Anthocerotales	Anthocerotaceae	*Anthoceros punctatus* L.	1169281.9	515804.6	1,3
57	Hornworts	Notothyladales	Notothyladaceae	*Phaeoceros laevis* (L.) Prosk.	104539.8	42422.8	1,3
58	Mosses	Funariales	Funariaceae	*Physcomitrella patens* (Hedw.) Bruch & Schimp.	1094541.9	614164.0	3,6
59	Mosses	Funariales	Funariaceae	*Funaria hygrometrica* Hedw.	224594.0	12198.0	1,3
60	Mosses	Polytrichales	Polytrichaceae	*Polytrichum juniperinum* Hedw.	71619.8	16164.3	1,3
61	Mosses	Hedwigiales	Hedwigiaceae	*Hedwigia ciliata* (Hedw.) P.Beauv.	66455.7	18696.0	1,3
62	Mosses	Hypnales	Hylocomiaceae	*Hylocomium splendens* (Hedw.) Schimp. in B.S.G.	128415.1	31244.7	1,3
63	Liverworts	Marchantiales	Conocephalaceae	*Conocephalum conicum* (Linnaeus) Lindb.	578811.1	278995.2	1,3
64	Liverworts	Marchantiales	Marchantiaceae	*Marchantia polymorpha* L.	688452.2	170412.7	1,3
65	Liverworts	Marchantiales	Ricciaceae	*Riccia fluitans* L.	2485774.0	1164875.4	1,3
66	Algae	Charophytes	Characeae	*Chara vulgaris* Linnaeus	102878.6	52095.2	2,5
67	Algae	Charophytes	Klebsormidiaceae	*Klebsormidium elegans* Lokhorst	753175.9	111691.7	1,3
68	Algae	Chlorophyta	Caulerpaceae	*Caulerpa prolifera* (P. Forsskal) Lamouroux	1134470.0	415566.0	1,3
69	Algae	Rhodophyta	Halymeniaceae	*Halymenia floresii* (Clemente y Rubio) C. Agardh	432744.0	180677.8	1,3
70	Lichen	Lecanorales	Parmeliaceae	*Cetraria islandica* (L.) Ach.	1771409.5	435555.6	1,3
71	Lichen	Teloschistales	Teloschistaceae	*Xanthoria parietina* (L.) Th. Fr.	529803.4	263644.0	1,3

Supplementary Table S10: Concentrations of oleic acid or OA (18:1, Δ^9) (pmol/g plant weight) found in the 71 plant species analyzed. Values are reported as the mean (X) and standard deviation (SD) of at least triplicates (n) of at least one plant species (N).

#	Group	Order	Family	Species	OA (pmol/g PW)		N,n
					X	SD	
1	Angiosperms	Santalales	Viscaceae	*Viscum album* L.	3474485.8	444252.5	1,3
2	Angiosperms	Brassicales	Brassicaceae	*Arabidopsis thaliana* (L.) Heynh.	202056.5	131762.4	2,5
3	Angiosperms	Malvales	Malvaceae	*Theobroma cacao* L.	13301.4	4933.9	1,3
4	Angiosperms	Rosales	Cannabaceae	*Cannabis sativa* L.	11611.2	766.2	2,5
5	Angiosperms	Malpighiales	Rhizophoraceae	*Rhizophora mangle* L.	23740.1	3738.5	1,3
6	Angiosperms	Malpighiales	Salicaceae	*Salix glabra* Scop.	115809.4	29684.7	1,3
7	Angiosperms	Poales	Poaceae	*Sorghum bicolor* (L.) Moench	96455.1	5072.7	1,3
8	Angiosperms	Poales	Poaceae	*Hakonechloa macra* (Munro) Makino	208392.0	26622.9	1,3
9	Angiosperms	Poales	Poaceae	*Zea mays* L.	237610.3	48238.4	1,3
10	Angiosperms	Asparagales	Amaryllidaceae	*Allium sativum* L.	248219.6	91016.9	1,3
11	Angiosperms	Magnoliales	Magnoliaceae	*Liriodendron tulipifera* L.	275535.6	51812.8	1,3
12	Angiosperms	Chloranthales	Chloranthaceae	*Chloranthus spicatus* (Thunb.) Makino	127290.9	11839.2	1,3
13	Angiosperms	Austrobaileyales	Schisandraceae	*Schisandra chinensis* (Turcz.) Baill.	484856.8	351168.5	1,3
14	Angiosperms	Nymphaeales	Nymphaeaceae	*Nymphaea nouchali* Burm.f.	837041.8	303712.5	1,3
15	Angiosperms	Amborellales	Amborellaceae	*Amborella trichopoda* Baill.	209565.5	14870.6	1,3
16	Gymnosperms	Gnetidae	Welwitschiaceae	*Welwitschia mirabilis* Hook.f.	611839.4	446557.9	1,3
17	Gymnosperms	Pinales	Pinaceae	*Pinus peuce* Griseb.	304375.5	174014.0	2,5
18	Gymnosperms	Pinales	Pinaceae	*Pinus sylvestris* L.	154184.2	69232.4	1,3
19	Gymnosperms	Pinales	Pinaceae	*Pinus mugo* Turra	187990.0	31077.9	1,3
20	Gymnosperms	Pinales	Pinaceae	*Pinus cembra* L.	29163.6	9782.5	1,3
21	Gymnosperms	Pinales	Pinaceae	*Larix gmelinii* var. *principis-rupprechtii* (Mayr) Pilg.	23364.1	9661.4	1,3
22	Gymnosperms	Pinales	Pinaceae	*Picea abies* (L.) H.Karst.	59011.9	17941.6	1,3
23	Gymnosperms	Pinales	Pinaceae	*Abies numidica* de Lannoy ex Carrière	32813.6	14841.3	1,3
24	Gymnosperms	Pinales	Pinaceae	*Abies cephalonica* Loudon	97161.0	40128.2	1,3
25	Gymnosperms	Pinales	Pinaceae	*Abies koreana* E.H.Wilson	175975.3	54230.5	1,3
26	Gymnosperms	Cupressales/Pinales	Taxaceae	*Taxus baccata* L.	30235.1	8727.0	1,4
27	Gymnosperms	Cupressales	Cupressaceae	*Cryptomeria japonica* (Thunb. ex L.f.) D.Don	243521.3	21140.8	1,3
28	Gymnosperms	Cupressales	Cupressaceae	*Thujopsis dolabrata* (Thunb. ex L.f.) Sieb. & Zucc.	6584.9	5624.6	1,3
29	Gymnosperms	Araucariales	Araucariaceae	*Araucaria araucana* (Molina) K.Koch	293943.8	258920.8	2,5
30	Gymnosperms	Ginkgoales	Ginkgoaceae	*Ginkgo biloba* L.	201566.5	91539.9	2,5
31	Gymnosperms	Cycadales	Cycadaceae	*Cycas revoluta* Thunb.	269000.4	236354.6	2,5
32	Monilophytes	Polypodiales	Polypodiaceae	*Polypodium vulgare* L.	85032.6	99862.5	2,5
33	Monilophytes	Polypodiales	Davalliaceae	*Davallia canariensis* (L.) Sm.	10430.6	6158.4	1,3
34	Monilophytes	Polypodiales	Tectariaceae	*Tectaria zeylanica* (Houtt.) Sledge	64569.9	24652.4	1,3
35	Monilophytes	Polypodiales	Dryopteridaceae	*Polystichum aculeatum* (L.) Roth ex Mert.	60318.1	12317.9	1,3
36	Monilophytes	Polypodiales	Onocleaceae	*Onoclea sensibilis* L.	3913.5	2262.6	1,3
37	Monilophytes	Polypodiales	Blechnaceae	*Blechnum spicant* (L.) Sm.	3402.4	399.5	1,3
38	Monilophytes	Polypodiales	Thelypteridaceae	*Thelypteris palustri* Schott	6243.1	767.4	1,3
39	Monilophytes	Polypodiales	Cystopteridaceae	*Gymnocarpium robertianum* (Hoffm.) Newman	18566.0	5911.5	1,3
40	Monilophytes	Polypodiales	Aspleniaceae	*Asplenium trichomanes* L.	121704.7	20336.9	1,3
41	Monilophytes	Polypodiales	Aspleniaceae	*Phyllitis scolopendrium* (L.) Newman	47145.7	8189.4	1,3
42	Monilophytes	Polypodiales	Pteridaceae	*Adiantum venustum* D. Don	191052.5	31386.5	1,3
43	Monilophytes	Cyatheales	Cyatheaceae	*Sphaeropteris cooperi* (F.Muell.) R.M.Tryon	24462.9	29845.5	1,3
44	Monilophytes	Salviniales	Salviniaceae	*Salvinia natans* (L.) All.	19665.2	9931.5	1,3
45	Monilophytes	Salviniales	Salviniaceae	*Salvinia molesta* D. S. Mitch.	204435.9	138793.8	2,5
46	Monilophytes	Schizaeales	Anemiaceae	*Anemia phyllitidis* (L.) Sw.	28092.1	8547.9	1,3
47	Monilophytes	Schizaeales	Lygodiaceae	*Lygodium volubile* Sw.	36193893.9	19220024.7	1,3
48	Monilophytes	Osmundales	Osmundaceae	*Osmunda regalis* L.	4504.4	1715.7	1,3
49	Monilophytes	Marattiales	Marattiaceae	*Angiopteris evecta* (G.Forst.) Hoffm.	16962.5	1992.3	1,3
50	Monilophytes	Equisetales	Equisetaceae	*Equisetum trachyodon* (A. Braun) W.D.J. Koch	23284.1	11061.8	1,3
51	Monilophytes	Psilotales	Psilotaceae	*Psilotum nudum* (L.) P. Beauv.	72989.6	12759.5	1,3
52	Lycophytes	Selaginellales	Selaginellaceae	*Selaginella moellendorffii* Hieron.	115827.5	16158.8	1,3
53	Lycophytes	Selaginellales	Selaginellaceae	*Selaginella pallescens* (C.Presl) Spring	367551.7	116581.8	2,6
54	Lycophytes	Lycopodiales	Lycopodiaceae	*Huperzia phlegmaria* (L.) Rothm.	761513.4	207154.3	1,3
55	Hornworts	Anthocerotales	Anthocerotaceae	*Anthoceros agrestis* Paton nom. cons. prop.	13781.5	12447.6	1,4
56	Hornworts	Anthocerotales	Anthocerotaceae	*Anthoceros punctatus* L.	71427.9	-	1,3
57	Hornworts	Notothyladales	Notothyladaceae	*Phaeoceros laevis* (L.) Prosk.	221707.8	42261.1	1,3
58	Mosses	Funariales	Funariaceae	*Physcomitrella patens* (Hedw.) Bruch & Schimp.	61825.5	84664.0	3,6
59	Mosses	Funariales	Funariaceae	*Funaria hygrometrica* Hedw.	584339.2	91246.0	1,3
60	Mosses	Polytrichales	Polytrichaceae	*Polytrichum juniperinum* Hedw.	63304.6	19460.6	1,3
61	Mosses	Hedwigiales	Hedwigiaceae	*Hedwigia ciliata* (Hedw.) P.Beauv.	43020.7	10517.8	1,3
62	Mosses	Hypnales	Hylocomiaceae	*Hylocomium splendens* (Hedw.) Schimp. in B.S.G.	63219.0	23140.3	1,3
63	Liverworts	Marchantiales	Conocephalaceae	*Conocephalum conicum* (Linnaeus) Lindb.	137532.4	61545.0	1,3
64	Liverworts	Marchantiales	Marchantiaceae	*Marchantia polymorpha* L.	689884.8	76270.7	1,3
65	Liverworts	Marchantiales	Ricciaceae	*Riccia fluitans* L.	986353.5	338543.4	1,3
66	Algae	Charophytes	Characeae	*Chara vulgaris* Linnaeus	87249.4	62518.9	2,5
67	Algae	Charophytes	Klebsormidiaceae	*Klebsormidium elegans* Lokhorst	68705.7	81424.2	1,3
68	Algae	Chlorophyta	Caulerpaceae	*Caulerpa prolifera* (P. Forsskal) Lamouroux	38333.1	10803.4	1,3
69	Algae	Rhodophyta	Halymeniaceae	*Halymenia floresii* (Clemente y Rubio) C. Agardh	37672.8	7825.5	1,3
70	Lichen	Lecanorales	Parmeliaceae	*Cetraria islandica* (L.) Ach.	1782066.9	499417.9	1,3
71	Lichen	Teloschistales	Teloschistaceae	*Xanthoria parietina* (L.) Th. Fr.	296859.3	13559.2	1,3

Supplementary Table S11: Concentrations of jasmonic acid (JA) (pmol/g plant weight) found in the 71 plant species analyzed. Values are reported as the mean (X) and standard deviation (SD) of at least triplicates (n) of at least one plant species (N).

#	Group	Order	Family	Species	JA (pmol/g PW)		N,n
					X	SD	
1	Angiosperms	Santalales	Viscaceae	*Viscum album* L.	3119.5	1621.3	1,3
2	Angiosperms	Brassicales	Brassicaceae	*Arabidopsis thaliana* (L.) Heynh.	8477.2	2617.0	2,5
3	Angiosperms	Malvales	Malvaceae	*Theobroma cacao* L.	79.8	1.9	1,3
4	Angiosperms	Rosales	Cannabaceae	*Cannabis sativa* L.	21.6	10.4	2,5
5	Angiosperms	Malpighiales	Rhizophoraceae	*Rhizophora mangle* L.	<LOD	-	1,3
6	Angiosperms	Malpighiales	Salicaceae	*Salix glabra* Scop.	6764.2	326.5	1,3
7	Angiosperms	Poales	Poaceae	*Sorghum bicolor* (L.) Moench	599.5	483.0	1,3
8	Angiosperms	Poales	Poaceae	*Hakonechloa macra* (Munro) Makino	513.1	333.8	1,3
9	Angiosperms	Poales	Poaceae	*Zea mays* L.	337.0	112.4	1,3
10	Angiosperms	Asparagales	Amaryllidaceae	*Allium sativum* L.	172.4	63.3	1,3
11	Angiosperms	Magnoliales	Magnoliaceae	*Liriodendron tulipifera* L.	2069.6	1260.1	1,3
12	Angiosperms	Chloranthales	Chloranthaceae	*Chloranthus spicatus* (Thunb.) Makino	90.0	49.5	1,3
13	Angiosperms	Austrobaileyales	Schisandraceae	*Schisandra chinensis* (Turcz.) Baill.	201.6	27.1	1,3
14	Angiosperms	Nymphaeales	Nymphaeaceae	*Nymphaea nouchali* Burm.f.	720.6	455.3	1,3
15	Angiosperms	Amborellales	Amborellaceae	*Amborella trichopoda* Baill.	2170.1	1517.5	1,3
16	Gymnosperms	Gnetidae	Welwitschiaceae	*Welwitschia mirabilis* Hook.f.	48.5	29.9	1,3
17	Gymnosperms	Pinales	Pinaceae	*Pinus peuce* Griseb.	284.0	281.4	2,5
18	Gymnosperms	Pinales	Pinaceae	*Pinus sylvestris* L.	69.8	7.0	1,3
19	Gymnosperms	Pinales	Pinaceae	*Pinus mugo* Turra	17.5	2.4	1,3
20	Gymnosperms	Pinales	Pinaceae	*Pinus cembra* L.	41.3	11.1	1,3
21	Gymnosperms	Pinales	Pinaceae	*Larix gmelinii* var. *principis-rupprechtii* (Mayr) Pilg.	209.8	164.4	1,3
22	Gymnosperms	Pinales	Pinaceae	*Picea abies* (L.) H.Karst.	11.0	1.5	1,3
23	Gymnosperms	Pinales	Pinaceae	*Abies numidica* de Lannoy ex Carrière	30.6	6.8	1,3
24	Gymnosperms	Pinales	Pinaceae	*Abies cephalonica* Loudon	64.4	9.6	1,3
25	Gymnosperms	Pinales	Pinaceae	*Abies koreana* E.H.Wilson	13.6	0.02	1,3
26	Gymnosperms	Cupressales/Pinales	Taxaceae	*Taxus baccata* L.	39.2	25.4	1,4
27	Gymnosperms	Cupressales	Cupressaceae	*Cryptomeria japonica* (Thunb. ex L.f.) D.Don	26.0	6.1	1,3
28	Gymnosperms	Cupressales	Cupressaceae	*Thujopsis dolabrata* (Thunb. ex L.f.) Sieb. & Zucc.	<LOD	-	1,3
29	Gymnosperms	Araucariales	Araucariaceae	*Araucaria araucana* (Molina) K.Koch	175.8	155.4	2,5
30	Gymnosperms	Ginkgoales	Ginkgoaceae	*Ginkgo biloba* L.	355.8	147.5	2,5
31	Gymnosperms	Cycadales	Cycadaceae	*Cycas revoluta* Thunb.	150.2	123.6	2,5
32	Monilophytes	Polypodiales	Polypodiaceae	*Polypodium vulgare* L.	<LOD	-	2,5
33	Monilophytes	Polypodiales	Davalliaceae	*Davallia canariensis* (L.) Sm.	<LOD	-	1,3
34	Monilophytes	Polypodiales	Tectariaceae	*Tectaria zeylanica* (Houtt.) Sledge	<LOD	-	1,3
35	Monilophytes	Polypodiales	Dryopteridaceae	*Polystichum aculeatum* (L.) Roth ex Mert.	<LOD	-	1,3
36	Monilophytes	Polypodiales	Onocleaceae	*Onoclea sensibilis* L.	23.3	10.9	1,3
37	Monilophytes	Polypodiales	Blechnaceae	*Blechnum spicant* (L.) Sm.	32.0	19.6	1,3
38	Monilophytes	Polypodiales	Thelypteridaceae	*Thelypteris palustri* Schott	167.0	65.6	1,3
39	Monilophytes	Polypodiales	Cystopteridaceae	*Gymnocarpium robertianum* (Hoffm.) Newman	13.7	3.9	1,3
40	Monilophytes	Polypodiales	Aspleniaceae	*Asplenium trichomanes* L.	<LOD	-	1,3
41	Monilophytes	Polypodiales	Aspleniaceae	*Phyllitis scolopendrium* (L.) Newman	<LOD	-	1,3
42	Monilophytes	Polypodiales	Pteridaceae	*Adiantum venustum* D. Don	<LOD	-	1,3
43	Monilophytes	Cyatheales	Cyatheaceae	*Sphaeropteris cooperi* (F.Muell.) R.M.Tryon	<LOD	-	1,3
44	Monilophytes	Salviniales	Salviniaceae	*Salvinia natans* (L.) All.	127.4	117.1	1,3
45	Monilophytes	Salviniales	Salviniaceae	*Salvinia molesta* D. S. Mitch.	292.1	151.2	2,5
46	Monilophytes	Schizaeales	Anemiaceae	*Anemia phyllitidis* (L.) Sw.	<LOD	-	1,3
47	Monilophytes	Schizaeales	Lygodiaceae	*Lygodium volubile* Sw.	47.9	27.8	1,3
48	Monilophytes	Osmundales	Osmundaceae	*Osmunda regalis* L.	5.3	0.7	1,3
49	Monilophytes	Marattiales	Marattiaceae	*Angiopteris evecta* (G.Forst.) Hoffm.	38.2	17.0	1,3
50	Monilophytes	Equisetales	Equisetaceae	*Equisetum trachyodon* (A. Braun) W.D.J. Koch	12.5	7.2	1,3
51	Monilophytes	Psilotales	Psilotaceae	*Psilotum nudum* (L.) P. Beauv.	<LOD	-	1,3
52	Lycophytes	Selaginellales	Selaginellaceae	*Selaginella moellendorffii* Hieron.	<LOD	-	1,3
53	Lycophytes	Selaginellales	Selaginellaceae	*Selaginella pallescens* (C.Presl) Spring	15.3	9.7	2,6
54	Lycophytes	Lycopodiales	Lycopodiaceae	*Huperzia phlegmaria* (L.) Rothm.	<LOD	-	1,3
55	Hornworts	Anthocerotales	Anthocerotaceae	*Anthoceros agrestis* Paton nom. cons. prop.	349.6	166.7	1,4
56	Hornworts	Anthocerotales	Anthocerotaceae	*Anthoceros punctatus* L.	177.0	53.4	1,3
57	Hornworts	Notothyladales	Notothyladaceae	*Phaeoceros laevis* (L.) Prosk.	110.5	37.5	1,3
58	Mosses	Funariales	Funariaceae	*Physcomitrella patens* (Hedw.) Bruch & Schimp.	<LOD	-	3,6
59	Mosses	Funariales	Funariaceae	*Funaria hygrometrica* Hedw.	5.9	2.5	1,3
60	Mosses	Polytrichales	Polytrichaceae	*Polytrichum juniperinum* Hedw.	6.0	4.0	1,3
61	Mosses	Hedwigiales	Hedwigiaceae	*Hedwigia ciliata* (Hedw.) P.Beauv.	3.9	0.3	1,3
62	Mosses	Hypnales	Hylocomiaceae	*Hylocomium splendens* (Hedw.) Schimp. in B.S.G.	<LOD	-	1,3
63	Liverworts	Marchantiales	Conocephalaceae	*Conocephalum conicum* (Linnaeus) Lindb.	12.4	4.6	1,3
64	Liverworts	Marchantiales	Marchantiaceae	*Marchantia polymorpha* L.	41.2	5.7	1,3
65	Liverworts	Marchantiales	Ricciaceae	*Riccia fluitans* L.	<LOD	-	1,3
66	Algae	Charophytes	Characeae	*Chara vulgaris* Linnaeus	<LOD	-	2,5
67	Algae	Charophytes	Klebsormidiaceae	*Klebsormidium elegans* Lokhorst	<LOD	-	1,3
68	Algae	Chlorophyta	Caulerpaceae	*Caulerpa prolifera* (P. Forsskal) Lamouroux	<LOD	-	1,3
69	Algae	Rhodophyta	Halymeniaceae	*Halymenia floresii* (Clemente y Rubio) C. Agardh	<LOD	-	1,3
70	Lichen	Lecanorales	Parmeliaceae	*Cetraria islandica* (L.) Ach.	<LOD	-	1,3
71	Lichen	Teloschistales	Teloschistaceae	*Xanthoria parietina* (L.) Th. Fr.	<LOD	-	1,3

Supplementary Table S12: Concentrations of α-linolenic acid or ALA (18:3, $\Delta^{9,12,15}$, ω-3), γ-linolenic acid or GLA (18:3, $\Delta^{6,9,12}$, ω-6) and the unassigned ALA/GLA (pmol/g plant weight) found in the 71 plant species analyzed. Values are reported as the mean (X) and standard deviation (SD) of at least triplicates (n) of at least one plant species (N). The identification of ALA and GLA was only performed in the 33 plant species re-analyzed.

#	Family	Species	ALA (pmol/g PW)		GLA (pmol/g PW)		ALA/GLA (pmol/g PW)		N,n
			X	SD	X	SD	X	SD	
1	Viscaceae	*Viscum album* L.	-	-	-	-	4496077.5	725438.7	1,3
2	Brassicaceae	*Arabidopsis thaliana* (L.) Heynh.	8194124.5	3681668.4	<LOD	-	-	-	2,5
3	Malvaceae	*Theobroma cacao* L.	134418.0	11179.6	<LOD	-	-	-	1,3
4	Cannabaceae	*Cannabis sativa* L.	1808337.4	2192407.7	<LOD	-	-	-	2,5
5	Rhizophoraceae	*Rhizophora mangle* L.	-	-	-	-	91943.8	31669.8	1,3
6	Salicaceae	*Salix glabra* Scop.	-	-	-	-	7648653.3	1809990.6	1,3
7	Poaceae	*Sorghum bicolor* (L.) Moench	8157827.5	833091.2	<LOD	-	-	-	1,3
8	Poaceae	*Hakonechloa macra* (Munro) Makino	-	-	-	-	2822300.8	690398.1	1,3
9	Poaceae	*Zea mays* L.	-	-	-	-	6898186.9	925575.0	1,3
10	Amaryllidaceae	*Allium sativum* L.	-	-	-	-	10590274.5	4994807.6	1,3
11	Magnoliaceae	*Liriodendron tulipifera* L.	-	-	-	-	7573521.2	2113716.4	1,3
12	Chloranthaceae	*Chloranthus spicatus* (Thunb.) Makino	-	-	-	-	662719.3	62801.3	1,3
13	Schisandraceae	*Schisandra chinensis* (Turcz.) Baill.	-	-	-	-	1592200.4	1087104.6	1,3
14	Nymphaeaceae	*Nymphaea nouchali* Burm.f.	-	-	-	-	16234685.1	2966303.6	1,3
15	Amborellaceae	*Amborella trichopoda* Baill.	1339452.9	19810.8	<LOD	-	-	-	1,3
16	Welwitschiaceae	*Welwitschia mirabilis* Hook.f.	2716818.0	1082124.9	<LOD	-	-	-	1,3
17	Pinaceae	*Pinus peuce* Griseb.	357370.2	178286.4	26898.8	13419.4	-	-	2,5
18	Pinaceae	*Pinus sylvestris* L.	747810.9	503053.7	30558.0	3023.0	-	-	1,3
19	Pinaceae	*Pinus mugo* Turra	354784.5	35599.7	39420.5	3955.5	-	-	1,3
20	Pinaceae	*Pinus cembra* L.	30578.2	11421.5	3779.3	1411.6	-	-	1,3
21	Pinaceae	*Larix gmelinii* var. *principis-rupprechtii* (Mayr) Pilg.	11336.9	4082.4	1259.7	453.6	-	-	1,3
22	Pinaceae	*Picea abies* (L.) H.Karst.	98512.4	8474.2	3046.8	262.1	-	-	1,3
23	Pinaceae	*Abies numidica* de Lannoy ex Carrière	297247.1	275440.4	3002.5	2782.2	-	-	1,3
24	Pinaceae	*Abies cephalonica* Loudon	735874.8	138296.3	<LOD	-	-	-	1,3
25	Pinaceae	*Abies koreana* E.H.Wilson	204296.2	26706.8	10752.4	1405.6	-	-	1,3
26	Taxaceae	*Taxus baccata* L.	7034.0	6120.4	<LOD	-	-	-	1,4
27	Cupressaceae	*Cryptomeria japonica* (Thunb. ex L.f.) D.Don	3345046.3	856093.4	<LOD	-	-	-	1,3
28	Cupressaceae	*Thujopsis dolabrata* (Thunb. ex L.f.) Sieb. & Zucc.	15102.0	3991.7	<LOD	-	-	-	1,3
29	Araucariaceae	*Araucaria araucana* (Molina) K.Koch	929721.0	291327.9	<LOD	-	-	-	2,5
30	Ginkgoaceae	*Ginkgo biloba* L.	1253071.9	478841.4	<LOD	-	-	-	2,5
31	Cycadaceae	*Cycas revoluta* Thunb.	700868.2	534633.0	<LOD	-	-	-	2,5
32	Polypodiaceae	*Polypodium vulgare* L.	-	-	-	-	42293.6	35549.3	2,5
33	Davalliaceae	*Davallia canariensis* (L.) Sm.	-	-	-	-	31502.6	14105.3	1,3
34	Tectariaceae	*Tectaria zeylanica* (Houtt.) Sledge	-	-	-	-	15639.4	2280.9	1,3
35	Dryopteridaceae	*Polystichum aculeatum* (L.) Roth ex Mert.	-	-	-	-	51620.3	19574.4	1,3
36	Onocleaceae	*Onoclea sensibilis* L.	-	-	-	-	105559.3	15197.7	1,3
37	Blechnaceae	*Blechnum spicant* (L.) Sm.	-	-	-	-	13095.4	4135.2	1,3
38	Thelypteridaceae	*Thelypteris palustri* Schott	-	-	-	-	17266.7	12903.5	1,3
39	Cystopteridaceae	*Gymnocarpium robertianum* (Hoffm.) Newman	-	-	-	-	48275.7	8206.2	1,3
40	Aspleniaceae	*Asplenium trichomanes* L.	-	-	-	-	98746.8	17433.1	1,3
41	Aspleniaceae	*Phyllitis scolopendrium* (L.) Newman	-	-	-	-	4278.0	2910.0	1,3
42	Pteridaceae	*Adiantum venustum* D. Don	-	-	-	-	35003.1	54935.3	1,3
43	Cyatheaceae	*Sphaeropteris cooperi* (F.Muell.) R.M.Tryon	-	-	-	-	236239.3	103394.9	1,3
44	Salviniaceae	*Salvinia natans* (L.) All.	-	-	-	-	269278.2	129952.6	1,3
45	Salviniaceae	*Salvinia molesta* D. S. Mitch.	423584.2	167371.0	13100.5	5176.4	-	-	2,5
46	Anemiaceae	*Anemia phyllitidis* (L.) Sw.	-	-	-	-	11489.4	1873.4	1,3
47	Lygodiaceae	*Lygodium volubile* Sw.	-	-	-	-	273628.4	165303.4	1,3
48	Osmundaceae	*Osmunda regalis* L.	-	-	-	-	25689.1	2522.6	1,3
49	Marattiaceae	*Angiopteris evecta* (G.Forst.) Hoffm.	-	-	-	-	471476.0	37673.5	1,3
50	Equisetaceae	*Equisetum trachyodon* (A. Braun) W.D.J. Koch	277456.8	124374.7	<LOD	-	-	-	1,3
51	Psilotaceae	*Psilotum nudum* (L.) P. Beauv.	120366.3	23942.4	<LOD	-	-	-	1,3
52	Selaginellaceae	*Selaginella moellendorffii* Hieron.	1032473.4	55357.7	<LOD	-	-	-	1,3
53	Selaginellaceae	*Selaginella pallescens* (C.Presl) Spring	1165578.3	900330.2	<LOD	-	-	-	2,6
54	Lycopodiaceae	*Huperzia phlegmaria* (L.) Rothm.	9928.5	4481.3	<LOD	-	-	-	1,3
55	Anthocerotaceae	*Anthoceros agrestis* Paton nom. cons. prop.	57078.1	9242.3	<LOD	-	-	-	1,4
56	Anthocerotaceae	*Anthoceros punctatus* L.	-	-	-	-	70046.2	14818.5	1,3
57	Notothyladaceae	*Phaeoceros laevis* (L.) Prosk.	-	-	-	-	443016.5	126038.1	1,3
58	Funariaceae	*Physcomitrella patens* (Hedw.) Bruch & Schimp.	758794.5	852341.9	93783.6	105345.6	-	-	3,6
59	Funariaceae	*Funaria hygrometrica* Hedw.	-	-	-	-	1669254.6	303237.1	1,3
60	Polytrichaceae	*Polytrichum juniperinum* Hedw.	-	-	-	-	91419.2	35740.3	1,3
61	Hedwigiaceae	*Hedwigia ciliata* (Hedw.) P.Beauv.	-	-	-	-	35230.6	17134.6	1,3
62	Hylocomiaceae	*Hylocomium splendens* (Hedw.) Schimp. in B.S.G.	105730.7	65462.9	<LOD	-	-	-	1,3
63	Conocephalaceae	*Conocephalum conicum* (Linnaeus) Lindb.	2881321.0	4335.5	250549.7	377.0	-	-	1,3
64	Marchantiaceae	*Marchantia polymorpha* L.	-	-	-	-	1413672.1	302800.5	1,3
65	Ricciaceae	*Riccia fluitans* L.	-	-	-	-	934388.0	370558.0	1,3
66	Characeae	*Chara vulgaris* Linnaeus	54239.7	42892.6	<LOD	-	-	-	2,5
67	Klebsormidiaceae	*Klebsormidium elegans* Lokhorst	-	-	-	-	823764.8	102027.2	1,3
68	Caulerpaceae	*Caulerpa prolifera* (P. Forsskal) Lamouroux	-	-	-	-	88301.6	51835.2	1,3
69	Halymeniaceae	*Halymenia floresii* (Clemente y Rubio) C. Agardh	-	-	-	-	12771.3	5466.5	1,3
70	Parmeliaceae	*Cetraria islandica* (L.) Ach.	-	-	-	-	12403.3	6679.3	1,3
71	Teloschistaceae	*Xanthoria parietina* (L.) Th. Fr.	723213.7	53696.6	<LOD	-	-	-	1,3

Supplementary Table S13: Concentrations of dihomo-γ-linolenic acid or DHGLA (20:3, $\Delta^{8,11,14}$, ω-6), sciadonic acid or ScA (20:3, $\Delta^{5,11,14}$, ω-6) and the unassigned DHGLA/ScA (pmol/g plant weight) found in the 71 plant species analyzed. Values are reported as the mean (X) and standard deviation (SD) of at least triplicates (n) of at least one plant species (N). The identification of DHGLA and ScA was only performed in the 33 plant species re-analyzed.

#	Family	Species	DHGLA (pmol/g PW)		ScAc (pmol/g PW)		DHGLA/ScA (pmol/g PW)		N,n
			X	SD	X	SD	X	SD	
1	Viscaceae	*Viscum album* L.	<LOD	-	<LOD	-	<LOD	-	1,3
2	Brassicaceae	*Arabidopsis thaliana* (L.) Heynh.	<LOD	-	<LOD	-	<LOD	-	2,5
3	Malvaceae	*Theobroma cacao* L.	<LOD	-	<LOD	-	<LOD	-	1,3
4	Cannabaceae	*Cannabis sativa* L.	<LOD	-	<LOD	-	<LOD	-	2,5
5	Rhizophoraceae	*Rhizophora mangle* L.	<LOD	-	<LOD	-	<LOD	-	1,3
6	Salicaceae	*Salix glabra* Scop.	<LOD	-	<LOD	-	<LOD	-	1,3
7	Poaceae	*Sorghum bicolor* (L.) Moench	<LOD	-	<LOD	-	<LOD	-	1,3
8	Poaceae	*Hakonechloa macra* (Munro) Makino	<LOD	-	<LOD	-	<LOD	-	1,3
9	Poaceae	*Zea mays* L.	<LOD	-	<LOD	-	8713.9	2850.9	1,3
10	Amaryllidaceae	*Allium sativum* L.	<LOD	-	<LOD	-	<LOD	-	1,3
11	Magnoliaceae	*Liriodendron tulipifera* L.	<LOD	-	<LOD	-	12528.6	2102.4	1,3
12	Chloranthaceae	*Chloranthus spicatus* (Thunb.) Makino	<LOD	-	<LOD	-	<LOD	-	1,3
13	Schisandraceae	*Schisandra chinensis* (Turcz.) Baill.	<LOD	-	<LOD	-	<LOD	-	1,3
14	Nymphaeaceae	*Nymphaea nouchali* Burm.f.	<LOD	-	<LOD	-	71054.6	28729.4	1,3
15	Amborellaceae	*Amborella trichopoda* Baill.	<LOD	-	<LOD	-	<LOD	-	1,3
16	Welwitschiaceae	*Welwitschia mirabilis* Hook.f.	<LOD	-	<LOD	-	<LOD	-	1,3
17	Pinaceae	*Pinus peuce* Griseb.	<LOD	-	120816.4	74680.4	-	-	2,5
18	Pinaceae	*Pinus sylvestris* L.	<LOD	-	261768.2	35776.2	-	-	1,3
19	Pinaceae	*Pinus mugo* Turra	7079.8	454.7	110916.8	7123.2	-	-	1,3
20	Pinaceae	*Pinus cembra* L.	<lod	-	18661.0	6329.1	-	-	1,3
21	Pinaceae	*Larix gmelinii* var. *principis-rupprechtii* (Mayr) Pilg.	<lod	-	3060.5	463.3	-	-	1,3
22	Pinaceae	*Picea abies* (L.) H.Karst.	447.1	2.7	5940.6	36.4	-	-	1,3
23	Pinaceae	*Abies numidica* de Lannoy ex Carrière	<lod	-	57816.0	57824.0	-	-	1,3
24	Pinaceae	*Abies cephalonica* Loudon	9278.8	2885.2	93819.1	29172.4	-	-	1,3
25	Pinaceae	*Abies koreana* E.H.Wilson	4939.6	27.5	44456.4	247.3	-	-	1,3
26	Taxaceae	*Taxus baccata* L.	<LOD	-	<LOD	-	<LOD	-	1,4
27	Cupressaceae	*Cryptomeria japonica* (Thunb. ex L.f.) D.Don	<LOD	-	720051.6	188565.7	-	-	1,3
28	Cupressaceae	*Thujopsis dolabrata* (Thunb. ex L.f.) Sieb. & Zucc.	<LOD	-	3591.3	2005.3	-	-	1,3
29	Araucariaceae	*Araucaria araucana* (Molina) K.Koch	<LOD	-	90034.2	32728.7	-	-	2,5
30	Ginkgoaceae	*Ginkgo biloba* L.	<LOD	-	25059.4	9759.0	-	-	2,5
31	Cycadaceae	*Cycas revoluta* Thunb.	<LOD	-	26983.9	24342.2	-	-	2,5
32	Polypodiaceae	*Polypodium vulgare* L.	<LOD	-	<LOD	-	<LOD	-	2,5
33	Davalliaceae	*Davallia canariensis* (L.) Sm.	<LOD	-	<LOD	-	<LOD	-	1,3
34	Tectariaceae	*Tectaria zeylanica* (Houtt.) Sledge	<LOD	-	<LOD	-	<LOD	-	1,3
35	Dryopteridaceae	*Polystichum aculeatum* (L.) Roth ex Mert.	<LOD	-	<LOD	-	<LOD	-	1,3
36	Onocleaceae	*Onoclea sensibilis* L.	<LOD	-	<LOD	-	<LOD	-	1,3
37	Blechnaceae	*Blechnum spicant* (L.) Sm.	<LOD	-	<LOD	-	<LOD	-	1,3
38	Thelypteridaceae	*Thelypteris palustri* Schott	<LOD	-	<LOD	-	<LOD	-	1,3
39	Cystopteridaceae	*Gymnocarpium robertianum* (Hoffm.) Newman	<LOD	-	<LOD	-	<LOD	-	1,3
40	Aspleniaceae	*Asplenium trichomanes* L.	<LOD	-	<LOD	-	<LOD	-	1,3
41	Aspleniaceae	*Phyllitis scolopendrium* (L.) Newman	<LOD	-	<LOD	-	<LOD	-	1,3
42	Pteridaceae	*Adiantum venustum* D. Don	<LOD	-	<LOD	-	<LOD	-	1,3
43	Cyatheaceae	*Sphaeropteris cooperi* (F.Muell.) R.M.Tryon	<LOD	-	<LOD	-	<LOD	-	1,3
44	Salviniaceae	*Salvinia natans* (L.) All.	<LOD	-	<LOD	-	1219.6	591.9	1,3
45	Salviniaceae	*Salvinia molesta* D. S. Mitch.	20045.3	9514.0	8187.5	3886.0	-	-	2,5
46	Anemiaceae	*Anemia phyllitidis* (L.) Sw.	<LOD	-	<LOD	-	<LOD	-	1,3
47	Lygodiaceae	*Lygodium volubile* Sw.	<LOD	-	<LOD	-	96338.2	84945.9	1,3
48	Osmundaceae	*Osmunda regalis* L.	<LOD	-	<LOD	-	<LOD	-	1,3
49	Marattiaceae	*Angiopteris evecta* (G.Forst.) Hoffm.	<LOD	-	<LOD	-	<LOD	-	1,3
50	Equisetaceae	*Equisetum trachyodon* (A. Braun) W.D.J. Koch	<LOD	-	5588.5	973.7	-	-	1,3
51	Psilotaceae	*Psilotum nudum* (L.) P. Beauv.	<LOD	-	5898.0	1223.1	-	-	1,3
52	Selaginellaceae	*Selaginella moellendorffii* Hieron.	<LOD	-	38307.1	7286.0	-	-	1,3
53	Selaginellaceae	*Selaginella pallescens* (C.Presl) Spring	<LOD	-	29341.2	20686.7	-	-	2,6
54	Lycopodiaceae	*Huperzia phlegmaria* (L.) Rothm.	7211.1	4683.2	983.3	638.6	-	-	1,3
55	Anthocerotaceae	*Anthoceros agrestis* Paton nom. cons. prop.	<LOD	-	<LOD	-	<LOD	-	1,4
56	Anthocerotaceae	*Anthoceros punctatus* L.	<LOD	-	<LOD	-	<LOD	-	1,3
57	Notothyladaceae	*Phaeoceros laevis* (L.) Prosk.	<LOD	-	<LOD	-	7711.4	978.0	1,3
58	Funariaceae	*Physcomitrella patens* (Hedw.) Bruch & Schimp.	38067.3	14782.1	<LOD	-	-	-	3,6
59	Funariaceae	*Funaria hygrometrica* Hedw.	<LOD	-	<LOD	-	73012.4	3605.1	1,3
60	Polytrichaceae	*Polytrichum juniperinum* Hedw.	<LOD	-	<LOD	-	1009.1	483.1	1,3
61	Hedwigiaceae	*Hedwigia ciliata* (Hedw.) P.Beauv.	<LOD	-	<LOD	-	897.8	267.2	1,3
62	Hylocomiaceae	*Hylocomium splendens* (Hedw.) Schimp. in B.S.G.	3163.2	2507.3	<LOD	-	-	-	1,3
63	Conocephalaceae	*Conocephalum conicum* (Linnaeus) Lindb.	172806.0	56664.0	<LOD	-	-	-	1,3
64	Marchantiaceae	*Marchantia polymorpha* L.	<LOD	-	<LOD	-	56951.3	36469.2	1,3
65	Ricciaceae	*Riccia fluitans* L.	<LOD	-	<LOD	-	84220.8	61174.6	1,3
66	Characeae	*Chara vulgaris* Linnaeus	11472.4	9880.2	1714.3	1476.3	-	-	2,5
67	Klebsormidiaceae	*Klebsormidium elegans* Lokhorst	<LOD	-	<LOD	-	<LOD	-	1,3
68	Caulerpaceae	*Caulerpa prolifera* (P. Forsskal) Lamouroux	<LOD	-	<LOD	-	<LOD	-	1,3
69	Halymeniaceae	*Halymenia floresii* (Clemente y Rubio) C. Agardh	<LOD	-	<LOD	-	<LOD	-	1,3
70	Parmeliaceae	*Cetraria islandica* (L.) Ach.	<LOD	-	<LOD	-	<LOD	-	1,3
71	Teloschistaceae	*Xanthoria parietina* (L.) Th. Fr.	<LOD	-	<LOD	-	<LOD	-	1,3

Supplementary Table S14: Concentrations of adrenic acid or AdA (22:4, $\Delta^{7,10,13,16}$, ω-6) (pmol/g plant weight) found in the 71 plant species analyzed. Values are reported as the mean (X) and standard deviation (SD) of at least triplicates (n) of at least one plant species (N).

#	Group	Order	Family	Species	AdA (pmol/g PW)		N,n
					X	SD	
1	Angiosperms	Santalales	Viscaceae	*Viscum album* L.	<LOD	-	1,3
2	Angiosperms	Brassicales	Brassicaceae	*Arabidopsis thaliana* (L.) Heynh.	<LOD	-	2,5
3	Angiosperms	Malvales	Malvaceae	*Theobroma cacao* L.	<LOD	-	1,3
4	Angiosperms	Rosales	Cannabaceae	*Cannabis sativa* L.	<LOD	-	2,5
5	Angiosperms	Malpighiales	Rhizophoraceae	*Rhizophora mangle* L.	<LOD	-	1,3
6	Angiosperms	Malpighiales	Salicaceae	*Salix glabra* Scop.	<LOD	-	1,3
7	Angiosperms	Poales	Poaceae	*Sorghum bicolor* (L.) Moench	<LOD	-	1,3
8	Angiosperms	Poales	Poaceae	*Hakonechloa macra* (Munro) Makino	<LOD	-	1,3
9	Angiosperms	Poales	Poaceae	*Zea mays* L.	<LOD	-	1,3
10	Angiosperms	Asparagales	Amaryllidaceae	*Allium sativum* L.	<LOD	-	1,3
11	Angiosperms	Magnoliales	Magnoliaceae	*Liriodendron tulipifera* L.	<LOD	-	1,3
12	Angiosperms	Chloranthales	Chloranthaceae	*Chloranthus spicatus* (Thunb.) Makino	<LOD	-	1,3
13	Angiosperms	Austrobaileyales	Schisandraceae	*Schisandra chinensis* (Turcz.) Baill.	<LOD	-	1,3
14	Angiosperms	Nymphaeales	Nymphaeaceae	*Nymphaea nouchali* Burm.f.	<LOD	-	1,3
15	Angiosperms	Amborellales	Amborellaceae	*Amborella trichopoda* Baill.	<LOD	-	1,3
16	Gymnosperms	Gnetidae	Welwitschiaceae	*Welwitschia mirabilis* Hook.f.	<LOD	-	1,3
17	Gymnosperms	Pinales	Pinaceae	*Pinus peuce* Griseb.	<LOD	-	2,5
18	Gymnosperms	Pinales	Pinaceae	*Pinus sylvestris* L.	<LOD	-	1,3
19	Gymnosperms	Pinales	Pinaceae	*Pinus mugo* Turra	<LOD	-	1,3
20	Gymnosperms	Pinales	Pinaceae	*Pinus cembra* L.	<LOD	-	1,3
21	Gymnosperms	Pinales	Pinaceae	*Larix gmelinii* var. *principis-rupprechtii* (Mayr) Pilg.	<LOD	-	1,3
22	Gymnosperms	Pinales	Pinaceae	*Picea abies* (L.) H.Karst.	<LOD	-	1,3
23	Gymnosperms	Pinales	Pinaceae	*Abies numidica* de Lannoy ex Carrière	<LOD	-	1,3
24	Gymnosperms	Pinales	Pinaceae	*Abies cephalonica* Loudon	<LOD	-	1,3
25	Gymnosperms	Pinales	Pinaceae	*Abies koreana* E.H.Wilson	<LOD	-	1,3
26	Gymnosperms	Cupressales/Pinales	Taxaceae	*Taxus baccata* L.	<LOD	-	1,4
27	Gymnosperms	Cupressales	Cupressaceae	*Cryptomeria japonica* (Thunb. ex L.f.) D.Don	<LOD	-	1,3
28	Gymnosperms	Cupressales	Cupressaceae	*Thujopsis dolabrata* (Thunb. ex L.f.) Sieb. & Zucc.	<LOD	-	1,3
29	Gymnosperms	Araucariales	Araucariaceae	*Araucaria araucana* (Molina) K.Koch	<LOD	-	2,5
30	Gymnosperms	Ginkgoales	Ginkgoaceae	*Ginkgo biloba* L.	<LOD	-	2,5
31	Gymnosperms	Cycadales	Cycadaceae	*Cycas revoluta* Thunb.	<LOD	-	2,5
32	Monilophytes	Polypodiales	Polypodiaceae	*Polypodium vulgare* L.	192.6	89.2	2,5
33	Monilophytes	Polypodiales	Davalliaceae	*Davallia canariensis* (L.) Sm.	<LOD	-	1,3
34	Monilophytes	Polypodiales	Tectariaceae	*Tectaria zeylanica* (Houtt.) Sledge	<LOD	-	1,3
35	Monilophytes	Polypodiales	Dryopteridaceae	*Polystichum aculeatum* (L.) Roth ex Mert.	62.2	37.3	1,3
36	Monilophytes	Polypodiales	Onocleaceae	*Onoclea sensibilis* L.	<LOD	-	1,3
37	Monilophytes	Polypodiales	Blechnaceae	*Blechnum spicant* (L.) Sm.	<LOD	-	1,3
38	Monilophytes	Polypodiales	Thelypteridaceae	*Thelypteris palustri* Schott	<LOD	-	1,3
39	Monilophytes	Polypodiales	Cystopteridaceae	*Gymnocarpium robertianum* (Hoffm.) Newman	<LOD	-	1,3
40	Monilophytes	Polypodiales	Aspleniaceae	*Asplenium trichomanes* L.	278.0	204.4	1,3
41	Monilophytes	Polypodiales	Aspleniaceae	*Phyllitis scolopendrium* (L.) Newman	<LOD	-	1,3
42	Monilophytes	Polypodiales	Pteridaceae	*Adiantum venustum* D. Don	<LOD	-	1,3
43	Monilophytes	Cyatheales	Cyatheaceae	*Sphaeropteris cooperi* (F.Muell.) R.M.Tryon	<LOD	-	1,3
44	Monilophytes	Salviniales	Salviniaceae	*Salvinia natans* (L.) All.	21.7	5.9	1,3
45	Monilophytes	Salviniales	Salviniaceae	*Salvinia molesta* D. S. Mitch.	1840.2	2109.6	2,5
46	Monilophytes	Schizaeales	Anemiaceae	*Anemia phyllitidis* (L.) Sw.	<LOD	-	1,3
47	Monilophytes	Schizaeales	Lygodiaceae	*Lygodium volubile* Sw.	<LOD	-	1,3
48	Monilophytes	Osmundales	Osmundaceae	*Osmunda regalis* L.	<LOD	-	1,3
49	Monilophytes	Marattiales	Marattiaceae	*Angiopteris evecta* (G.Forst.) Hoffm.	<LOD	-	1,3
50	Monilophytes	Equisetales	Equisetaceae	*Equisetum trachyodon* (A. Braun) W.D.J. Koch	<LOD	-	1,3
51	Monilophytes	Psilotales	Psilotaceae	*Psilotum nudum* (L.) P. Beauv.	<LOD	-	1,3
52	Lycophytes	Selaginellales	Selaginellaceae	*Selaginella moellendorffii* Hieron.	<LOD	-	1,3
53	Lycophytes	Selaginellales	Selaginellaceae	*Selaginella pallescens* (C.Presl) Spring	<LOD	-	2,6
54	Lycophytes	Lycopodiales	Lycopodiaceae	*Huperzia phlegmaria* (L.) Rothm.	106.5	102.9	1,3
55	Hornworts	Anthocerotales	Anthocerotaceae	*Anthoceros agrestis* Paton nom. cons. prop.	360.4	293.0	1,4
56	Hornworts	Anthocerotales	Anthocerotaceae	*Anthoceros punctatus* L.	<LOD	-	1,3
57	Hornworts	Notothyladales	Notothyladaceae	*Phaeoceros laevis* (L.) Prosk.	2717.6	378.2	1,3
58	Mosses	Funariales	Funariaceae	*Physcomitrella patens* (Hedw.) Bruch & Schimp.	4165.5	5546.5	3,6
59	Mosses	Funariales	Funariaceae	*Funaria hygrometrica* Hedw.	2472.3	390.5	1,3
60	Mosses	Polytrichales	Polytrichaceae	*Polytrichum juniperinum* Hedw.	194.9	87.9	1,3
61	Mosses	Hedwigiales	Hedwigiaceae	*Hedwigia ciliata* (Hedw.) P.Beauv.	22.1	12.7	1,3
62	Mosses	Hypnales	Hylocomiaceae	*Hylocomium splendens* (Hedw.) Schimp. in B.S.G.	270.2	151.4	1,3
63	Liverworts	Marchantiales	Conocephalaceae	*Conocephalum conicum* (Linnaeus) Lindb.	3140.3	2019.2	1,3
64	Liverworts	Marchantiales	Marchantiaceae	*Marchantia polymorpha* L.	3547.2	2218.8	1,3
65	Liverworts	Marchantiales	Ricciaceae	*Riccia fluitans* L.	1590.9	1943.7	1,3
66	Algae	Charophytes	Characeae	*Chara vulgaris* Linnaeus	435.2	352.2	2,5
67	Algae	Charophytes	Klebsormidiaceae	*Klebsormidium elegans* Lokhorst	<LOD	-	1,3
68	Algae	Chlorophyta	Caulerpaceae	*Caulerpa prolifera* (P. Forsskal) Lamouroux	<LOD	-	1,3
69	Algae	Rhodophyta	Halymeniaceae	*Halymenia floresii* (Clemente y Rubio) C. Agardh	<LOD	-	1,3
70	Lichen	Lecanorales	Parmeliaceae	*Cetraria islandica* (L.) Ach.	<LOD	-	1,3
71	Lichen	Teloschistales	Teloschistaceae	*Xanthoria parietina* (L.) Th. Fr.	<LOD	-	1,3

Supplementary Table S15: Concentrations of docosahexaenoic acid or DHA (22:6, $\Delta^{4,7,10,13,16,19}$, ω-3) (pmol/g plant weight) found in the 71 plant species analyzed. Values are reported as the mean (X) and standard deviation (SD) of at least triplicates (n) of at least one plant species (N).

#	Group	Order	Family	Species	DHA (pmol/g PW))		N,n
					X	SD	
1	Angiosperms	Santalales	Viscaceae	*Viscum album* L.	<LOD	-	1,3
2	Angiosperms	Brassicales	Brassicaceae	*Arabidopsis thaliana* (L.) Heynh.	<LOD	-	2,5
3	Angiosperms	Malvales	Malvaceae	*Theobroma cacao* L.	<LOD	-	1,3
4	Angiosperms	Rosales	Cannabaceae	*Cannabis sativa* L.	<LOD	-	2,5
5	Angiosperms	Malpighiales	Rhizophoraceae	*Rhizophora mangle* L.	<LOD	-	1,3
6	Angiosperms	Malpighiales	Salicaceae	*Salix glabra* Scop.	<LOD	-	1,3
7	Angiosperms	Poales	Poaceae	*Sorghum bicolor* (L.) Moench	<LOD	-	1,3
8	Angiosperms	Poales	Poaceae	*Hakonechloa macra* (Munro) Makino	<LOD	-	1,3
9	Angiosperms	Poales	Poaceae	*Zea mays* L.	<LOD	-	1,3
10	Angiosperms	Asparagales	Amaryllidaceae	*Allium sativum* L.	<LOD	-	1,3
11	Angiosperms	Magnoliales	Magnoliaceae	*Liriodendron tulipifera* L.	<LOD	-	1,3
12	Angiosperms	Chloranthales	Chloranthaceae	*Chloranthus spicatus* (Thunb.) Makino	<LOD	-	1,3
13	Angiosperms	Austrobaileyales	Schisandraceae	*Schisandra chinensis* (Turcz.) Baill.	<LOD	-	1,3
14	Angiosperms	Nymphaeales	Nymphaeaceae	*Nymphaea nouchali* Burm.f.	7194.3	1241.2	1,3
15	Angiosperms	Amborellales	Amborellaceae	*Amborella trichopoda* Baill.	<LOD	-	1,3
16	Gymnosperms	Gnetidae	Welwitschiaceae	*Welwitschia mirabilis* Hook.f.	<LOD	-	1,3
17	Gymnosperms	Pinales	Pinaceae	*Pinus peuce* Griseb.	<LOD	-	2,5
18	Gymnosperms	Pinales	Pinaceae	*Pinus sylvestris* L.	<LOD	-	1,3
19	Gymnosperms	Pinales	Pinaceae	*Pinus mugo* Turra	<LOD	-	1,3
20	Gymnosperms	Pinales	Pinaceae	*Pinus cembra* L.	<LOD	-	1,3
21	Gymnosperms	Pinales	Pinaceae	*Larix gmelinii* var. *principis-rupprechtii* (Mayr) Pilg.	<LOD	-	1,3
22	Gymnosperms	Pinales	Pinaceae	*Picea abies* (L.) H.Karst.	<LOD	-	1,3
23	Gymnosperms	Pinales	Pinaceae	*Abies numidica* de Lannoy ex Carrière	<LOD	-	1,3
24	Gymnosperms	Pinales	Pinaceae	*Abies cephalonica* Loudon	<LOD	-	1,3
25	Gymnosperms	Pinales	Pinaceae	*Abies koreana* E.H.Wilson	<LOD	-	1,3
26	Gymnosperms	Cupressales/Pinales	Taxaceae	*Taxus baccata* L.	<LOD	-	1,4
27	Gymnosperms	Cupressales	Cupressaceae	*Cryptomeria japonica* (Thunb. ex L.f.) D.Don	<LOD	-	1,3
28	Gymnosperms	Cupressales	Cupressaceae	*Thujopsis dolabrata* (Thunb. ex L.f.) Sieb. & Zucc.	<LOD	-	1,3
29	Gymnosperms	Araucariales	Araucariaceae	*Araucaria araucana* (Molina) K.Koch	<LOD	-	2,5
30	Gymnosperms	Ginkgoales	Ginkgoaceae	*Ginkgo biloba* L.	<LOD	-	2,5
31	Gymnosperms	Cycadales	Cycadaceae	*Cycas revoluta* Thunb.	<LOD	-	2,5
32	Monilophytes	Polypodiales	Polypodiaceae	*Polypodium vulgare* L.	<LOD	-	2,5
33	Monilophytes	Polypodiales	Davalliaceae	*Davallia canariensis* (L.) Sm.	<LOD	-	1,3
34	Monilophytes	Polypodiales	Tectariaceae	*Tectaria zeylanica* (Houtt.) Sledge	<LOD	-	1,3
35	Monilophytes	Polypodiales	Dryopteridaceae	*Polystichum aculeatum* (L.) Roth ex Mert.	9519.6	1707.2	1,3
36	Monilophytes	Polypodiales	Onocleaceae	*Onoclea sensibilis* L.	<LOD	-	1,3
37	Monilophytes	Polypodiales	Blechnaceae	*Blechnum spicant* (L.) Sm.	<LOD	-	1,3
38	Monilophytes	Polypodiales	Thelypteridaceae	*Thelypteris palustri* Schott	<LOD	-	1,3
39	Monilophytes	Polypodiales	Cystopteridaceae	*Gymnocarpium robertianum* (Hoffm.) Newman	<LOD	-	1,3
40	Monilophytes	Polypodiales	Aspleniaceae	*Asplenium trichomanes* L.	<LOD	-	1,3
41	Monilophytes	Polypodiales	Aspleniaceae	*Phyllitis scolopendrium* (L.) Newman	<LOD	-	1,3
42	Monilophytes	Polypodiales	Pteridaceae	*Adiantum venustum* D. Don	<LOD	-	1,3
43	Monilophytes	Cyatheales	Cyatheaceae	*Sphaeropteris cooperi* (F.Muell.) R.M.Tryon	<LOD	-	1,3
44	Monilophytes	Salviniales	Salviniaceae	*Salvinia natans* (L.) All.	4518.4	1801.3	1,3
45	Monilophytes	Salviniales	Salviniaceae	*Salvinia molesta* D. S. Mitch.	14906.5	1341.4	2,5
46	Monilophytes	Schizaeales	Anemiaceae	*Anemia phyllitidis* (L.) Sw.	<LOD	-	1,3
47	Monilophytes	Schizaeales	Lygodiaceae	*Lygodium volubile* Sw.	<LOD	-	1,3
48	Monilophytes	Osmundales	Osmundaceae	*Osmunda regalis* L.	<LOD	-	1,3
49	Monilophytes	Marattiales	Marattiaceae	*Angiopteris evecta* (G.Forst.) Hoffm.	<LOD	-	1,3
50	Monilophytes	Equisetales	Equisetaceae	*Equisetum trachyodon* (A. Braun) W.D.J. Koch	<LOD	-	1,3
51	Monilophytes	Psilotales	Psilotaceae	*Psilotum nudum* (L.) P. Beauv.	<LOD	-	1,3
52	Lycophytes	Selaginellales	Selaginellaceae	*Selaginella moellendorffii* Hieron.	<LOD	-	1,3
53	Lycophytes	Selaginellales	Selaginellaceae	*Selaginella pallescens* (C.Presl) Spring	<LOD	-	2,6
54	Lycophytes	Lycopodiales	Lycopodiaceae	*Huperzia phlegmaria* (L.) Rothm.	<LOD	-	1,3
55	Hornworts	Anthocerotales	Anthocerotaceae	*Anthoceros agrestis* Paton nom. cons. prop.	<LOD	-	1,4
56	Hornworts	Anthocerotales	Anthocerotaceae	*Anthoceros punctatus* L.	<LOD	-	1,3
57	Hornworts	Notothyladales	Notothyladaceae	*Phaeoceros laevis* (L.) Prosk.	32359.0	7421.0	1,3
58	Mosses	Funariales	Funariaceae	*Physcomitrella patens* (Hedw.) Bruch & Schimp.	<LOD	-	3,6
59	Mosses	Funariales	Funariaceae	*Funaria hygrometrica* Hedw.	49449.7	11936.9	1,3
60	Mosses	Polytrichales	Polytrichaceae	*Polytrichum juniperinum* Hedw.	8816.2	5560.4	1,3
61	Mosses	Hedwigiales	Hedwigiaceae	*Hedwigia ciliata* (Hedw.) P.Beauv.	87869.9	31392.5	1,3
62	Mosses	Hypnales	Hylocomiaceae	*Hylocomium splendens* (Hedw.) Schimp. in B.S.G.	11080.7	6243.4	1,3
63	Liverworts	Marchantiales	Conocephalaceae	*Conocephalum conicum* (Linnaeus) Lindb.	46526.9	13399.3	1,3
64	Liverworts	Marchantiales	Marchantiaceae	*Marchantia polymorpha* L.	38756.9	15939.0	1,3
65	Liverworts	Marchantiales	Ricciaceae	*Riccia fluitans* L.	56252.6	31858.6	1,3
66	Algae	Charophytes	Characeae	*Chara vulgaris* Linnaeus	<LOD	-	2,5
67	Algae	Charophytes	Klebsormidiaceae	*Klebsormidium elegans* Lokhorst	<LOD	-	1,3
68	Algae	Chlorophyta	Caulerpaceae	*Caulerpa prolifera* (P. Forsskal) Lamouroux	44300.6	34373.3	1,3
69	Algae	Rhodophyta	Halymeniaceae	*Halymenia floresii* (Clemente y Rubio) C. Agardh	<LOD	-	1,3
70	Lichen	Lecanorales	Parmeliaceae	*Cetraria islandica* (L.) Ach.	<LOD	-	1,3
71	Lichen	Teloschistales	Teloschistaceae	*Xanthoria parietina* (L.) Th. Fr.	11685.7	6684.8	1,3

Supplementary Table S16: Concentrations (mean values in pmol/g plant weight) of the 14 analytes used for the Principal Component Analysis (PCA). The background colors of the cells are used to highlight concentrations in the column (i.e. concentrations of the respective analyte are coded red for small amounts, yellow for intermediate amounts and green for high amounts).

#	Group	*Species*	AA	JuA	2AG	2JG	AEA	JEA	OA	STE	MEA	LEA	OEA	SEA	PEA	JA
1	Angiosperms	*Viscum album*	0.0	0.0	0.0	0.0	0.0	0.0	3474485.8	1793985.8	0.0	9051.8	997.9	96.9	1420.0	3119.5
2	Angiosperms	*Arabidopsis thaliana*	0.0	0.0	0.0	0.0	0.0	0.0	202056.5	710665.7	48.4	17710.2	284.2	212.4	3588.6	8477.2
3	Angiosperms	*Theobroma cacao*	0.0	0.0	0.0	0.0	0.0	0.0	13301.4	52498.4	0.0	1870.1	93.9	40.8	306.7	79.8
4	Angiosperms	*Cannabis sativa*	0.0	0.0	0.0	0.0	0.0	0.0	11611.2	120666.4	3.7	416.5	15.9	98.1	127.5	21.6
5	Angiosperms	*Rhizophora mangle*	0.0	0.0	0.0	0.0	0.0	0.0	23740.1	110796.7	5.3	433.2	101.9	67.8	57.0	0.0
6	Angiosperms	*Salix glabra*	0.0	0.0	0.0	0.0	0.0	0.0	115809.4	575567.6	0.0	5250.1	88.1	0.0	630.9	6764.2
7	Angiosperms	*Sorghum bicolor*	0.0	0.0	0.0	0.0	0.0	0.0	96455.1	446422.6	8.7	4727.1	160.0	105.3	977.5	599.5
8	Angiosperms	*Hakonechloa macra*	0.0	0.0	0.0	0.0	0.0	0.0	208392.0	273283.0	9.5	2056.0	213.9	0.0	254.1	513.1
9	Angiosperms	*Zea mays*	0.0	0.0	0.0	0.0	0.0	0.0	237610.3	1286745.2	1.9	8279.9	228.3	142.2	1360.5	337.0
10	Angiosperms	*Allium sativum*	0.0	0.0	0.0	0.0	0.0	0.0	248219.6	988868.6	57.5	77053.3	1382.4	1419.9	16387.9	172.4
11	Angiosperms	*Liriodendron tulipifera*	0.0	0.0	0.0	0.0	0.0	0.0	275535.6	552722.3	0.0	4140.2	211.6	123.8	594.8	2069.6
12	Angiosperms	*Chloranthus spicatus*	0.0	0.0	0.0	0.0	0.0	0.0	127290.9	319188.7	0.0	3658.7	121.7	65.2	212.9	90.0
13	Angiosperms	*Schisandra chinensis*	0.0	0.0	0.0	0.0	0.0	0.0	484856.8	800284.3	0.0	2611.5	64.3	86.2	675.1	201.6
14	Angiosperms	*Nymphaea nouchali*	0.0	0.0	0.0	0.0	0.0	0.0	837041.8	1000273.1	11.9	6986.2	200.0	147.5	2746.3	720.6
15	Angiosperms	*Amborella trichopoda*	0.0	0.0	0.0	0.0	0.0	0.0	209565.5	420899.3	4.7	5421.6	261.0	157.2	1510.0	2170.1
16	Gymnosperms	*Welwitschia mirabilis*	0.0	0.0	0.0	0.0	0.0	0.0	611839.4	545012.0	20.1	2866.5	2681.4	879.6	1653.2	48.5
17	Gymnosperms	*Pinus peuce*	0.0	12895.8	0.0	468.7	0.0	23.8	304375.5	863726.7	0.0	892.3	131.2	71.3	278.2	284.0
18	Gymnosperms	*Pinus sylvestris*	0.0	76526.6	0.0	2375.0	0.0	229.8	154184.2	171460.5	0.0	986.5	74.3	37.3	155.7	69.8
19	Gymnosperms	*Pinus mugo*	0.0	18467.1	0.0	600.8	0.0	0.0	187990.0	105228.9	0.0	89.7	29.9	0.0	40.0	17.5
20	Gymnosperms	*Pinus cembra*	0.0	601.1	0.0	55.8	0.0	2.5	29163.6	98371.6	0.0	218.0	12.6	23.3	27.7	41.3
21	Gymnosperms	*Larix gmelinii*	0.0	54.4	0.0	37.4	0.0	27.6	23364.1	162640.8	0.0	79.6	12.8	23.8	20.9	209.8
22	Gymnosperms	*Picea abies*	0.0	4427.3	0.0	308.5	0.0	0.8	59011.9	40322.3	0.0	13.6	1.8	9.2	7.2	11.0
23	Gymnosperms	*Abies numidica*	0.0	10998.8	0.0	374.1	0.0	7.2	32813.6	58885.0	0.0	44.3	18.7	0.0	0.0	30.6
24	Gymnosperms	*Abies cephalonica*	0.0	38959.6	0.0	2695.1	0.0	7.4	97161.0	121607.9	0.0	18.0	4.3	0.0	0.0	64.4
25	Gymnosperms	*Abies koreana*	0.0	15811.0	0.0	697.4	0.0	4.6	175975.3	97141.6	0.0	75.8	32.0	0.0	0.0	13.6
26	Gymnosperms	*Taxus baccata*	0.0	0.0	0.0	0.0	0.0	0.0	30235.1	53896.6	1.6	11.8	4.6	6057.0	769.3	39.2
27	Gymnosperms	*Cryptomeria japonica*	0.0	100611.8	0.0	2163.8	0.0	69.3	243521.3	546965.6	0.0	983.7	56.6	0.0	107.5	26.0
28	Gymnosperms	*Thujopsis dolabrata*	0.0	770.4	0.0	0.0	0.0	0.0	6584.9	194562.6	0.0	53.0	5.6	0.0	58.2	0.0
29	Gymnosperms	*Araucaria araucana*	26773.4	80320.2	0.0	3208.1	0.0	118.1	293943.8	400852.4	0.0	521.7	93.7	0.0	182.5	175.8
30	Gymnosperms	*Ginkgo biloba*	0.0	216277.7	0.0	2795.6	0.0	254.3	201566.5	6173280.7	5.6	1399.0	755.1	76.3	651.1	355.8
31	Gymnosperms	*Cycas revoluta*	0.0	4216.1	0.0	118.2	0.0	11.8	269000.4	549507.1	0.0	3604.2	424.6	83.2	298.1	150.2
32	Monilophytes	*Polypodium vulgare*	44425.8	0.0	6909.4	0.0	11.0	0.0	85032.6	171579.6	13.4	94.0	15.1	7722.1	1140.2	0.0
33	Monilophytes	*Davallia canariensis*	2884.3	0.0	1298.1	0.0	0.0	0.0	10430.6	234286.1	0.0	36.3	3.7	7072.4	629.4	0.0
34	Monilophytes	*Tectaria zeylanica*	3848.3	0.0	3233.2	0.0	0.0	0.0	64569.9	71072.3	0.0	12.7	1.8	54.1	19.2	0.0
35	Monilophytes	*Polystichum aculeatum*	16165.1	0.0	5162.6	0.0	5.0	0.0	60318.1	144908.4	32.9	76.3	24.9	3443.5	1969.2	0.0
36	Monilophytes	*Onoclea sensibilis*	32079.4	0.0	2085.9	0.0	0.0	0.0	3913.5	133466.3	0.0	87.7	9.1	2053.0	268.0	23.3
37	Monilophytes	*Blechnum spicant*	7979.1	0.0	2995.3	0.0	0.0	0.0	3402.4	57955.2	0.0	41.2	3.8	350.9	59.2	32.0
38	Monilophytes	*Thelypteris palustri*	6753.9	0.0	2851.6	0.0	7.2	0.0	6243.1	89662.0	0.0	45.7	2.3	0.0	425.1	167.0
39	Monilophytes	*Gymnocarpium robertianum*	12083.0	0.0	3616.1	0.0	7.7	0.0	18566.0	154575.2	6.5	66.2	8.7	6262.4	1291.1	13.7
40	Monilophytes	*Asplenium trichomanes*	20835.6	0.0	16014.1	0.0	6.4	0.0	121704.7	148684.8	3.5	178.1	20.6	4379.2	1095.0	0.0
41	Monilophytes	*Phyllitis scolopendrium*	3516.8	0.0	1698.1	0.0	3.8	0.0	47145.7	76459.4	5.7	93.2	9.6	1945.2	482.8	0.0
42	Monilophytes	*Adiantum venustum*	75994.9	0.0	10783.1	0.0	0.0	0.0	191052.5	210498.2	1.6	59.2	7.6	0.0	40.2	0.0
43	Monilophytes	*Sphaeropteris cooperi*	4784.5	0.0	166.1	0.0	0.0	0.0	24462.9	138391.7	6.3	122.7	10.0	2054.9	368.8	0.0
44	Monilophytes	*Salvinia natans*	13175.0	0.0	2067.9	0.0	1.2	0.0	19665.2	26130.8	13.8	15.0	4.4	3748.3	1407.3	127.4
45	Monilophytes	*Salvinia molesta*	307394.3	0.0	11227.2	0.0	102.6	0.0	204435.9	430715.0	57.8	862.4	104.0	179.8	445.2	292.1
46	Monilophytes	*Anemia phyllitidis*	12143.7	0.0	1209.6	0.0	56.0	0.0	28092.1	63336.3	0.0	90.7	5.0	131.1	28.8	0.0
47	Monilophytes	*Lygodium volubile*	64628.3	0.0	1182.9	0.0	0.0	0.0	36193893.9	10819008.8	0.0	152.5	55.0	8170.9	2481.3	47.9
48	Monilophytes	*Osmunda regalis*	1386.0	0.0	409.9	0.0	0.0	0.0	4504.4	71277.2	0.0	62.6	6.7	35.3	58.3	5.3
49	Monilophytes	*Angiopteris evecta*	6125.1	0.0	379.7	0.0	6.9	0.0	16962.5	42068.9	0.0	29.6	2.1	0.0	9.5	38.2
50	Monilophytes	*Equisetum trachyodon*	0.0	4768.5	0.0	2748.9	0.0	7.4	23284.1	147520.5	0.0	57.8	2.4	81.7	91.9	12.5
51	Monilophytes	*Psilotum nudum*	0.0	0.0	0.0	176.1	0.0	4.7	72989.6	211052.2	0.0	191.2	13.2	192.2	258.9	0.0
52	Lycophytes	*Selaginella moellendorffii*	0.0	9985.0	0.0	1818.6	0.0	6.5	115827.5	249766.6	0.0	282.2	16.5	0.0	80.2	0.0
53	Lycophytes	*Selaginella pallescens*	0.0	15352.2	0.0	4394.7	0.0	15.4	367551.7	294576.8	25.2	768.9	33.7	179.1	176.1	15.3
54	Lycophytes	*Huperzia phlegmaria*	83663.7	0.0	2098.9	0.0	10.2	0.0	761513.4	395542.9	0.0	124.1	17.0	0.0	89.3	0.0
55	Hornworts	*Anthoceros agrestis*	69691.7	0.0	51037.2	0.0	258.9	0.0	13781.5	230231.6	58.8	240.2	41.4	130.4	258.1	349.6
56	Hornworts	*Anthoceros punctatus*	24687.6	0.0	21241.6	0.0	0.0	0.0	71427.9	1169281.9	330.9	165.4	148.3	0.0	699.9	177.0
57	Hornworts	*Phaeoceros laevis*	316375.9	0.0	37873.5	0.0	694.4	0.0	221707.8	104539.8	72.4	887.2	126.8	0.0	387.7	110.5
58	Mosses	*Physcomitrella patens*	2648874.2	0.0	286570.7	0.0	694.4	0.0	61825.5	1094541.9	209.5	491.6	132.7	0.0	563.6	0.0
59	Mosses	*Funaria hygrometrica*	898972.3	0.0	47555.5	0.0	148.7	0.0	584339.2	224594.0	14.4	146.5	21.2	0.0	69.0	5.9
60	Mosses	*Polytrichum juniperinum*	35394.6	0.0	2472.3	0.0	35.9	0.0	63304.6	71619.8	0.2	83.4	9.0	191.2	63.1	6.0
61	Mosses	*Hedwigia ciliata*	20046.8	0.0	728.5	0.0	15.4	0.0	43020.7	66455.7	0.0	38.0	7.0	0.0	47.7	3.9
62	Mosses	*Hylocomium splendens*	86608.3	0.0	6307.5	0.0	215.5	0.0	63219.0	128415.1	0.0	383.7	17.2	48.1	341.6	0.0
63	Liverworts	*Conocephalum conicum*	1233225.6	0.0	55780.9	0.0	518.3	0.0	137532.4	578811.1	5.8	420.2	59.4	0.0	244.2	12.4
64	Liverworts	*Marchantia polymorpha*	903496.0	0.0	81990.1	0.0	891.7	0.0	689884.8	688452.2	38.1	1650.9	258.0	150.7	591.3	41.2
65	Liverworts	*Riccia fluitans*	452189.2	0.0	58983.4	0.0	460.2	0.0	986353.5	2485774.0	137.7	1070.5	1147.7	538.5	2463.7	0.0
66	Algae	*Chara vulgaris*	203074.1	0.0	10047.4	0.0	28.9	0.0	87249.4	102878.6	5.2	67.2	17.5	0.0	101.4	0.0
67	Algae	*Klebsormidium elegans*	0.0	0.0	0.0	0.0	0.0	0.0	68705.7	753175.9	767.9	308.5	261.0	1093.0	1070.9	0.0
68	Algae	*Caulerpa prolifera*	74711.0	0.0	0.0	0.0	0.0	0.0	38333.1	1134470.0	0.0	32.4	11.8	0.0	173.8	0.0
69	Algae	*Halymenia floresii*	9959.3	0.0	1033.0	0.0	0.0	0.0	37672.8	432744.0	18.3	16.9	14.0	0.0	94.8	0.0
70	Lichen	*Cetraria islandica*	2708.8	0.0	0.0	0.0	0.0	0.0	1782066.9	1771409.5	0.0	205.4	40.8	0.0	72.7	0.0
71	Lichen	*Xanthoria parietina*	24535.4	0.0	1112.4	0.0	0.0	0.0	296859.3	529803.4	11.6	1872.4	71.9	216.4	183.5	0.0

Supplementary Table S17: Contribution of the variables and eigenvalues obtained for the Principal Component Analysis (PCA) using Pearson (n-1).

Contribution of the variables (%):

	F1	F2	F3	F4	F5	F6	F7	F8	F9	F10	F11	F12	F13	F14
AA	**21.449**	2.620	8.878	0.028	0.460	1.805	1.058	0.003	0.037	12.692	0.121	46.692	3.762	0.394
JuA	11.916	6.934	**14.786**	1.597	0.004	0.007	0.338	2.306	6.385	0.005	30.136	2.595	22.599	0.393
2AG	**21.550**	2.359	9.045	0.058	0.143	0.586	1.666	0.236	0.178	15.484	0.009	43.596	4.859	0.231
2JG	9.549	8.088	5.414	2.057	0.551	0.452	4.840	6.772	60.294	0.215	0.000	0.103	1.641	0.023
AEA	**18.757**	1.899	6.843	0.144	0.367	1.951	0.638	0.136	0.029	69.043	0.004	0.047	0.033	0.109
JEA	11.145	6.767	**13.696**	1.778	0.013	0.000	0.776	3.836	12.794	0.054	45.279	0.575	3.271	0.016
OA	0.431	3.889	6.052	**31.569**	2.036	0.022	1.211	10.257	1.222	0.003	12.200	1.834	28.976	0.299
STE	1.671	1.903	**18.613**	**17.450**	2.490	1.422	2.934	2.787	1.911	0.053	11.585	2.793	33.230	1.159
MEA	3.173	0.109	1.374	0.229	11.669	**77.471**	4.219	0.552	0.001	0.474	0.089	0.360	0.146	0.134
LEA	0.062	**22.767**	3.114	**11.712**	0.416	3.479	4.406	6.164	1.271	0.000	0.009	0.543	0.691	45.367
OEA	0.032	8.338	6.938	6.194	0.441	1.218	**45.217**	**18.763**	10.788	1.111	0.421	0.131	0.275	0.134
SEA	0.148	4.494	0.150	**19.673**	7.928	2.232	**19.112**	**40.983**	3.043	0.421	0.111	0.106	0.060	1.537
PEA	0.004	**26.540**	5.080	5.410	2.563	2.246	4.742	1.645	0.368	0.241	0.022	0.613	0.431	50.094
JA	0.113	3.294	0.016	2.099	**70.919**	7.110	8.842	5.560	1.681	0.204	0.013	0.012	0.027	0.110
Eigenvalue	2.923	2.530	2.402	1.972	0.990	0.925	0.715	0.585	0.432	0.303	0.119	0.051	0.039	0.015
Variability (%)	20.875	18.070	17.154	14.085	7.072	6.604	5.107	4.179	3.086	2.166	0.851	0.366	0.280	0.104
Cumulative %	20.875	38.945	56.099	70.184	77.257	83.861	88.968	93.147	96.233	98.399	99.251	99.617	99.896	100.000

8. Supplementary Figures:

a

STE
18:0

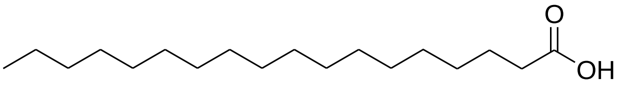

OA
18:1, Δ^{9}, $\omega-9$

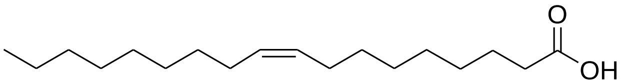

GLA
18:3, $\Delta^{6,9,12}$, $\omega-6$

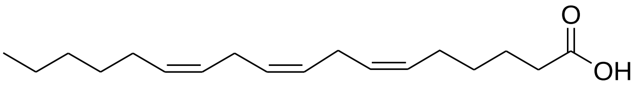

ALA
18:3, $\Delta^{9,12,15}$, $\omega-3$

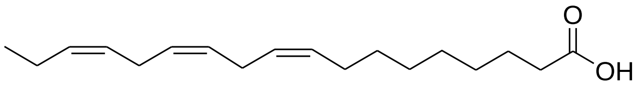

DHGLA
20:3, $\Delta^{8,11,14}$, $\omega-6$

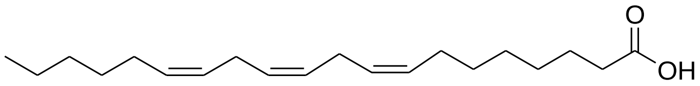

ScA
20:3, $\Delta^{5,11,14}$, $\omega-6$

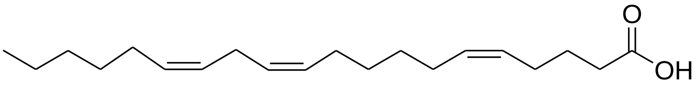

AA
20:4, $\Delta^{5,8,11,14}$, $\omega-6$

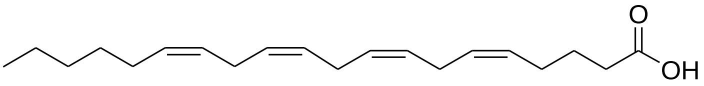

AA ω3
20:4, $\Delta^{8,11,14,17}$, $\omega-3$

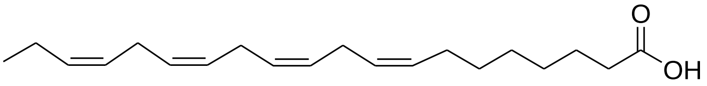

JuA
20:4, $\Delta^{5,11,14,17}$, $\omega-3$

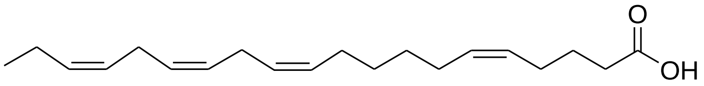

AdA
22:4, $\Delta^{7,10,13,16}$, $\omega-6$

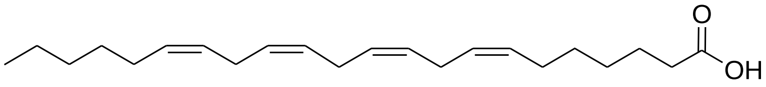

DHA
22:6, $\Delta^{4,7,10,13,16,19}$, $\omega-3$

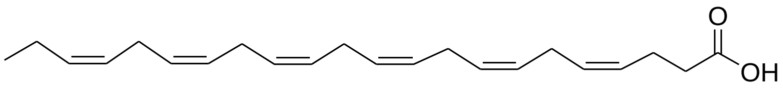

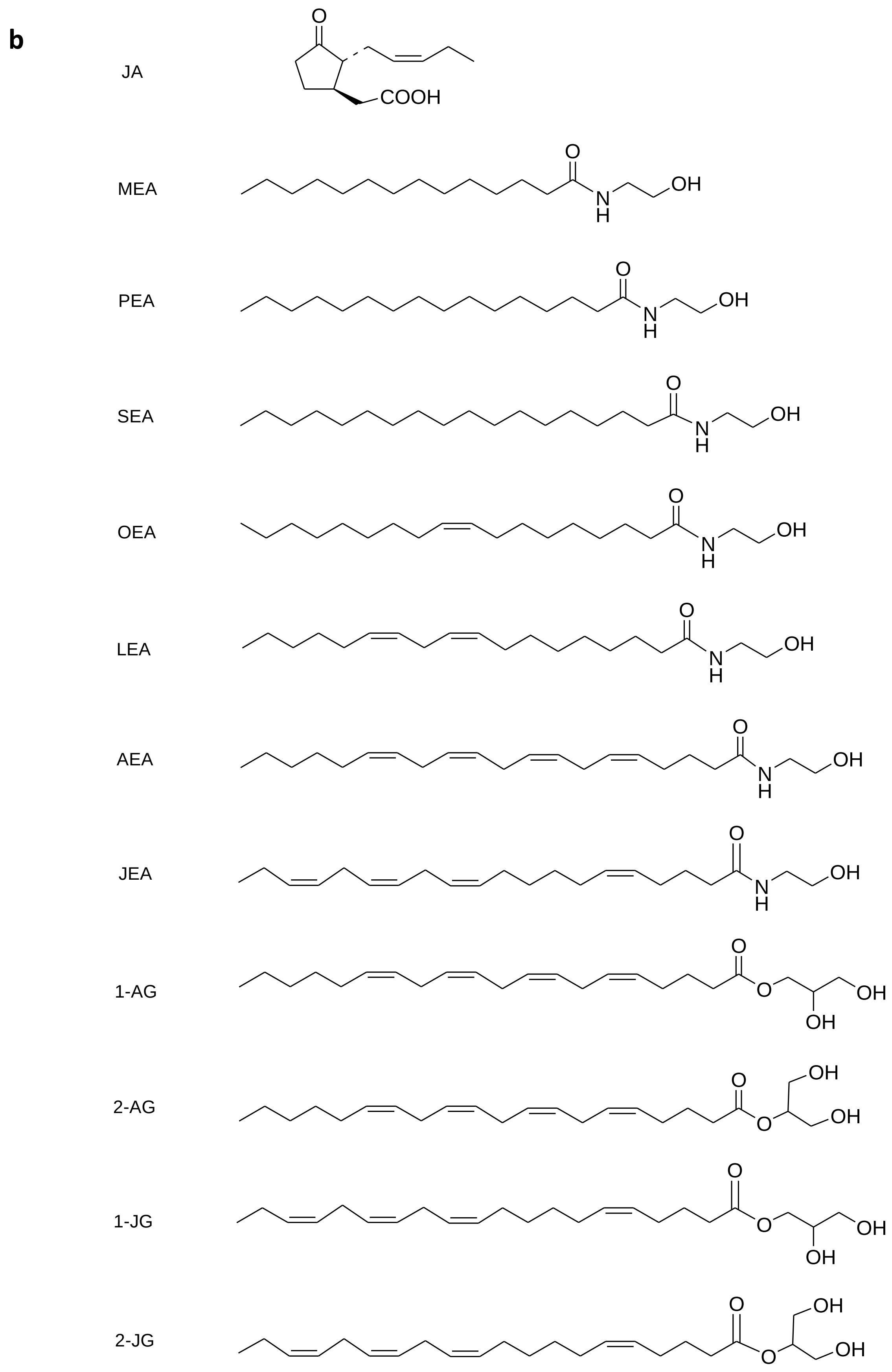

Supplementary Fig. S1. Chemical structure of the analytes investigated: a) fatty acids and b) fatty acid metabolites.

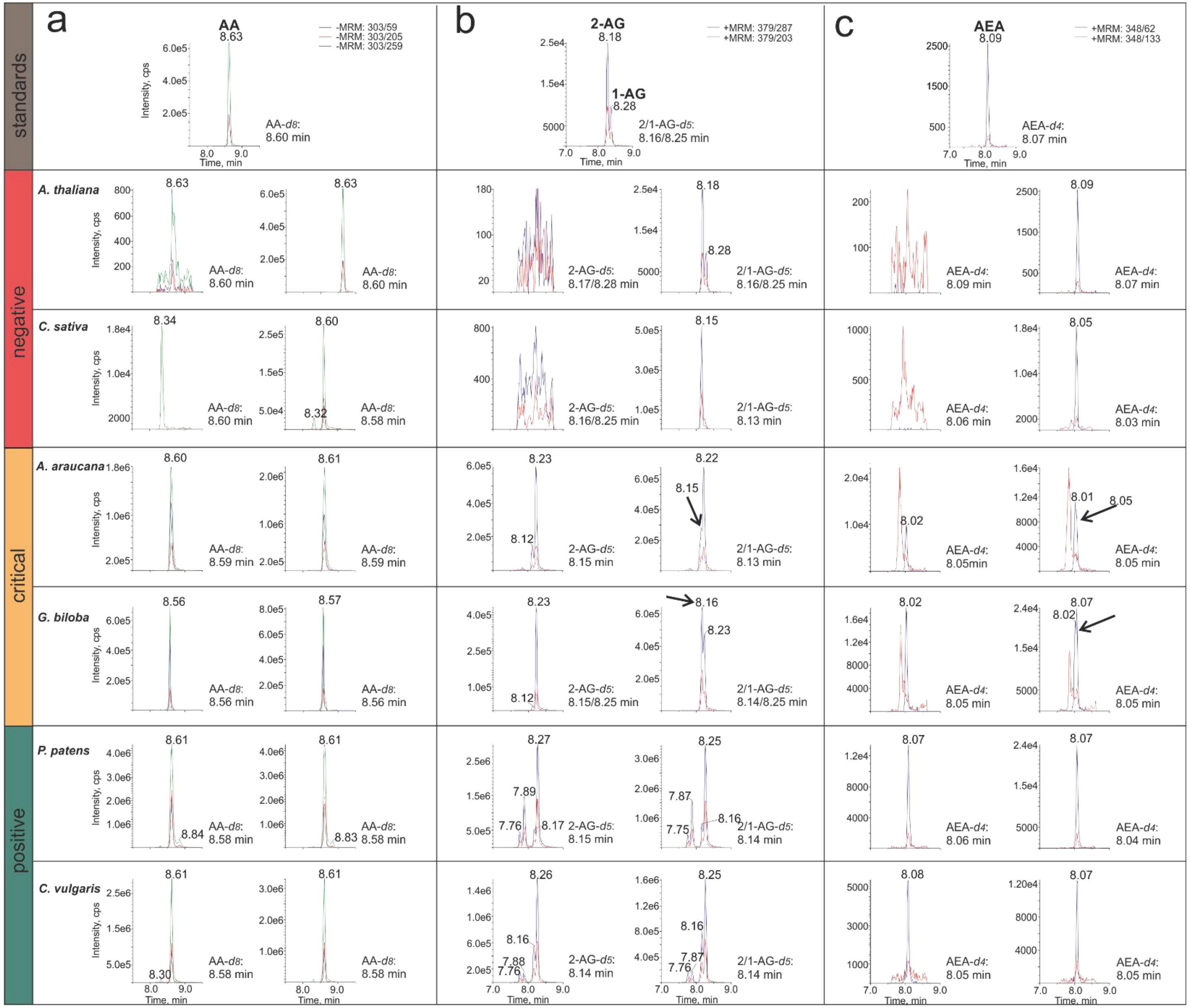
a
b
c
standards
negative
critical
positive
AA
8.63
-MRM: 303/59
-MRM: 303/205
-MRM: 303/259
AA-d8:
8.60 min
2-AG
8.18
1-AG
8.28
+MRM: 379/287
+MRM: 379/203
2/1-AG-d5:
8.16/8.25 min
AEA
8.09
+MRM: 348/62
+MRM: 348/133
AEA-d4:
8.07 min
A. thaliana
C. sativa
A. araucana
G. biloba
P. patens
C. vulgaris
Intensity, cps
Time, min

Supplementary Fig. S2. Chromatograms (LC-MS/MS) showing the analysis of AA (a), 1/2-AG (b) and AEA (c). Only the top row shows one chromatogram representing the standards alone process without matrix (i.e., 4000 ng/mL of AA, 400 ng/mL of 2-AG and 8 ng/mL of AEA). The following rows present two chromatograms, with the first corresponding to the analysis of the sample (plant species) and the second, with the same spiked sample. Next to each chromatogram, the retention time of the IS in the analysis is shown. The figure depicts examples of samples of plant species for which all three analytes were absent (negative), all three analytes were present (positive) and where is doubt about the identity of AA, 1/2-AG and AEA (critical). Arrows in some chromatograms confirm that the peaks shown in the samples did not correspond to the spiked analytes (serendipitous observation). Samples were prepared using SPE for cleanup (see Sample preparation for quantification) and analyzed using LC-MS/MS method 1 in the positive and negative modes (see Chromatographic conditions used for the quantifications).

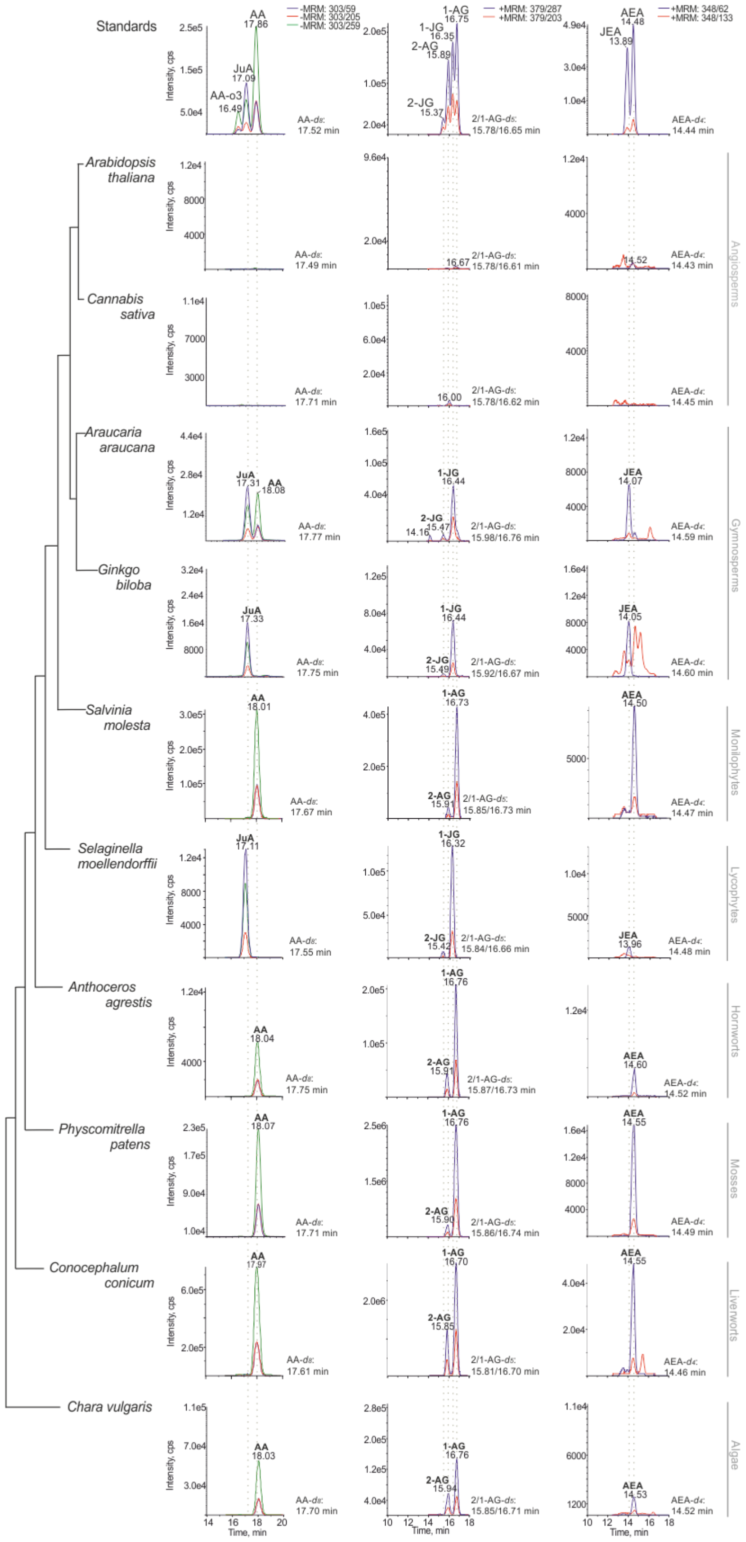

Supplementary Fig. S3. Chromatograms showing the analysis of C-20 PUFA metabolites. First row: AA and JuA, second row: 1/2-AG and 1/2-JG and third row: AEA and JEA. Samples were prepared using HPLC for cleanup (see Sample preparation for identification) and analyzed using LC-MS/MS in the positive and negative modes (see Chromatographic conditions used for the identification of structural isomers (LC-MS/MS method 2)).

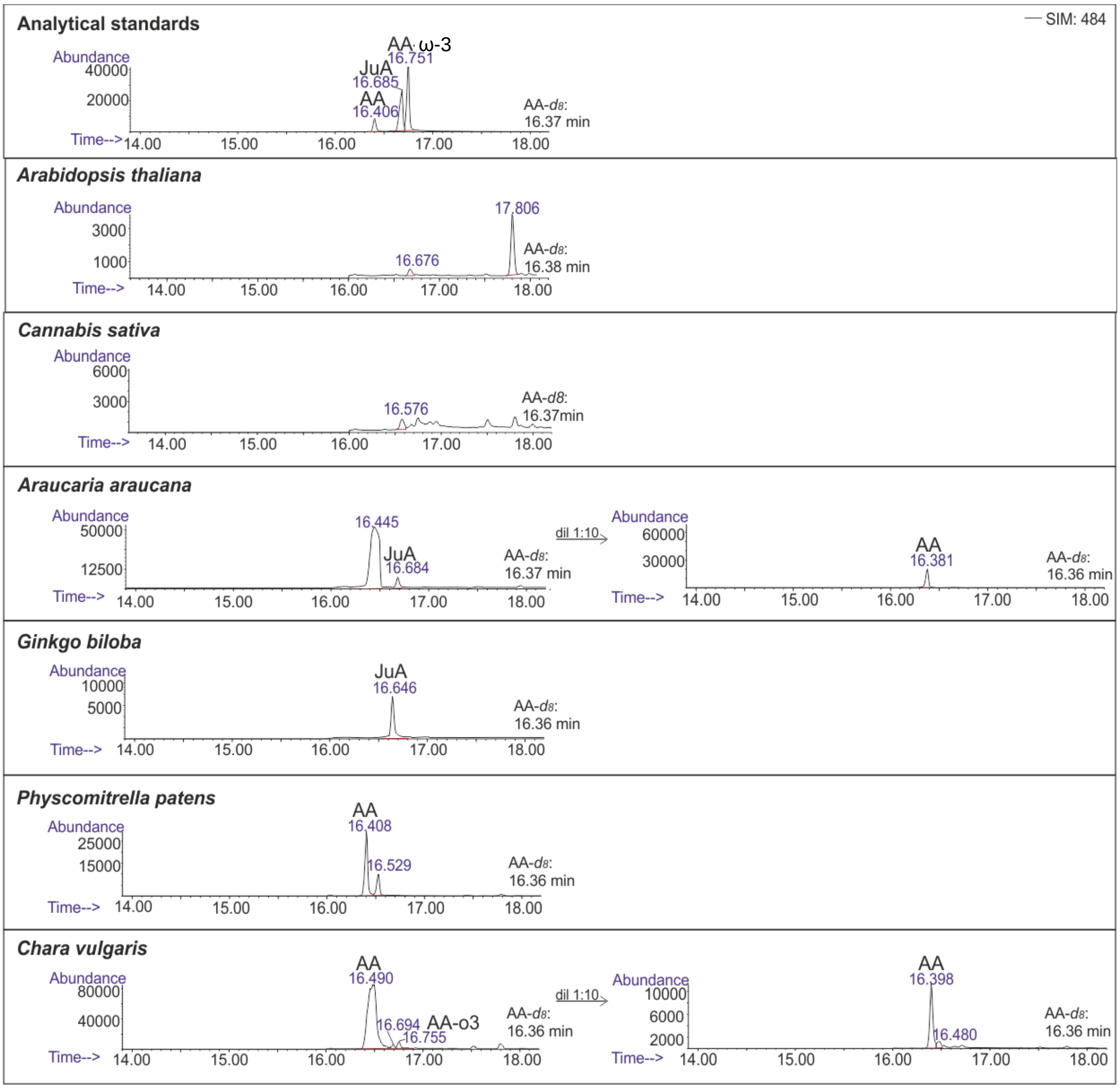

Supplementary Fig. S4. GC-MS chromatograms showing the analysis of AA, JuA and AA ω-3 (SIM *m/z* 484). Samples were prepared using HPLC for cleanup (see Sample preparation for identification) and analyzed using GC-MS (see GC-MS analysis). In the case of *Araucaria araucana* and *Chara vulgaris*, chromatograms of a dilution 1:10 are also shown in order to highlight the presences of AA and JuA or AA ω-3, respectively.

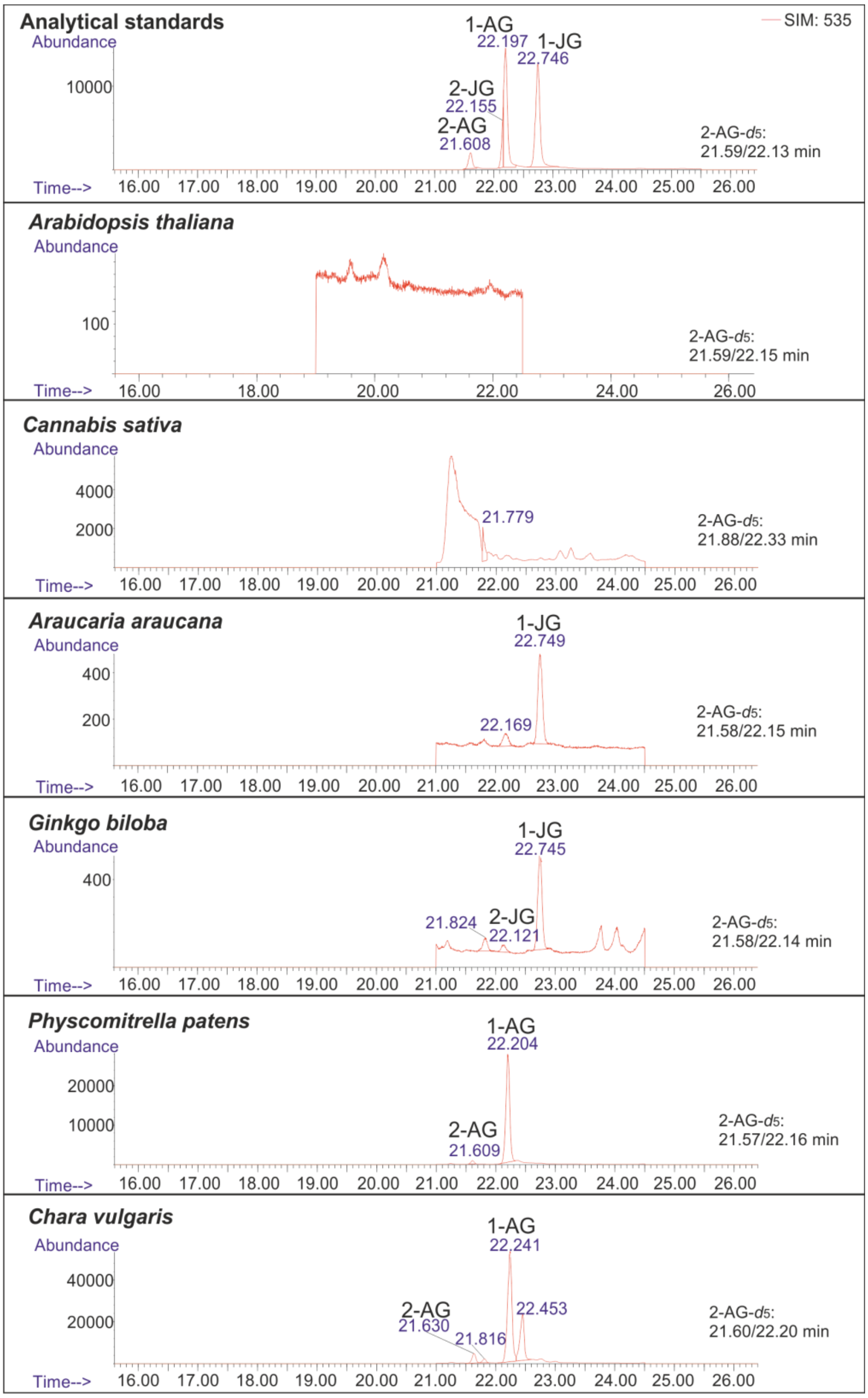

Supplementary Fig. S5. GC-MS chromatograms showing the analysis of 1/2-AG and 1/2-JG (SIM *m/z* 535). Samples were prepared using HPLC for cleanup (see Sample preparation for identification) and analyzed using GC-MS (see GC-MS analysis).

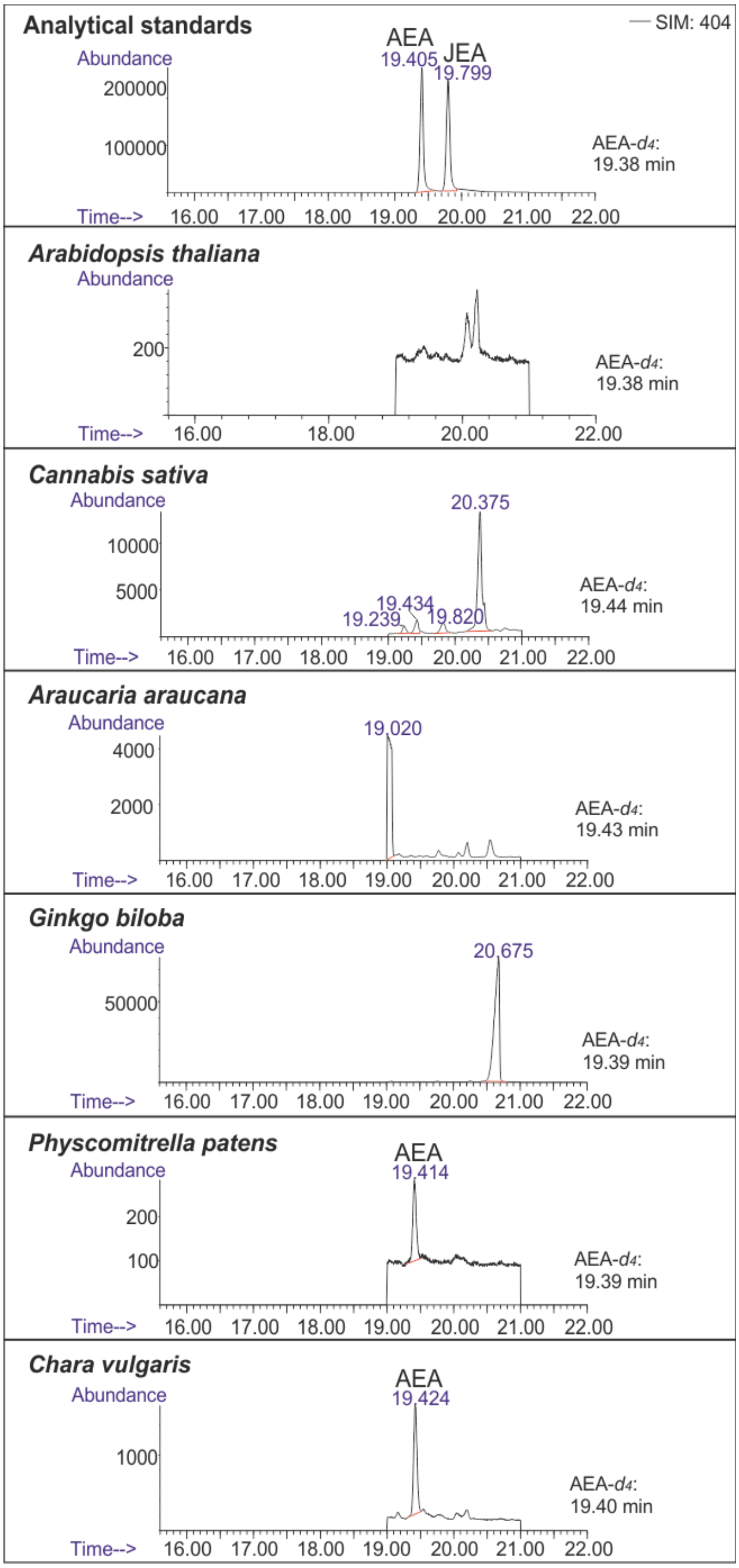

Supplementary Fig. S6. CG-MS chromatograms showing the analysis of AEA and JEA (SIM *m/z* 404). Samples were prepared using HPLC for cleanup (see Sample preparation for identification) and analyzed using GC-MS (see GC-MS analysis).

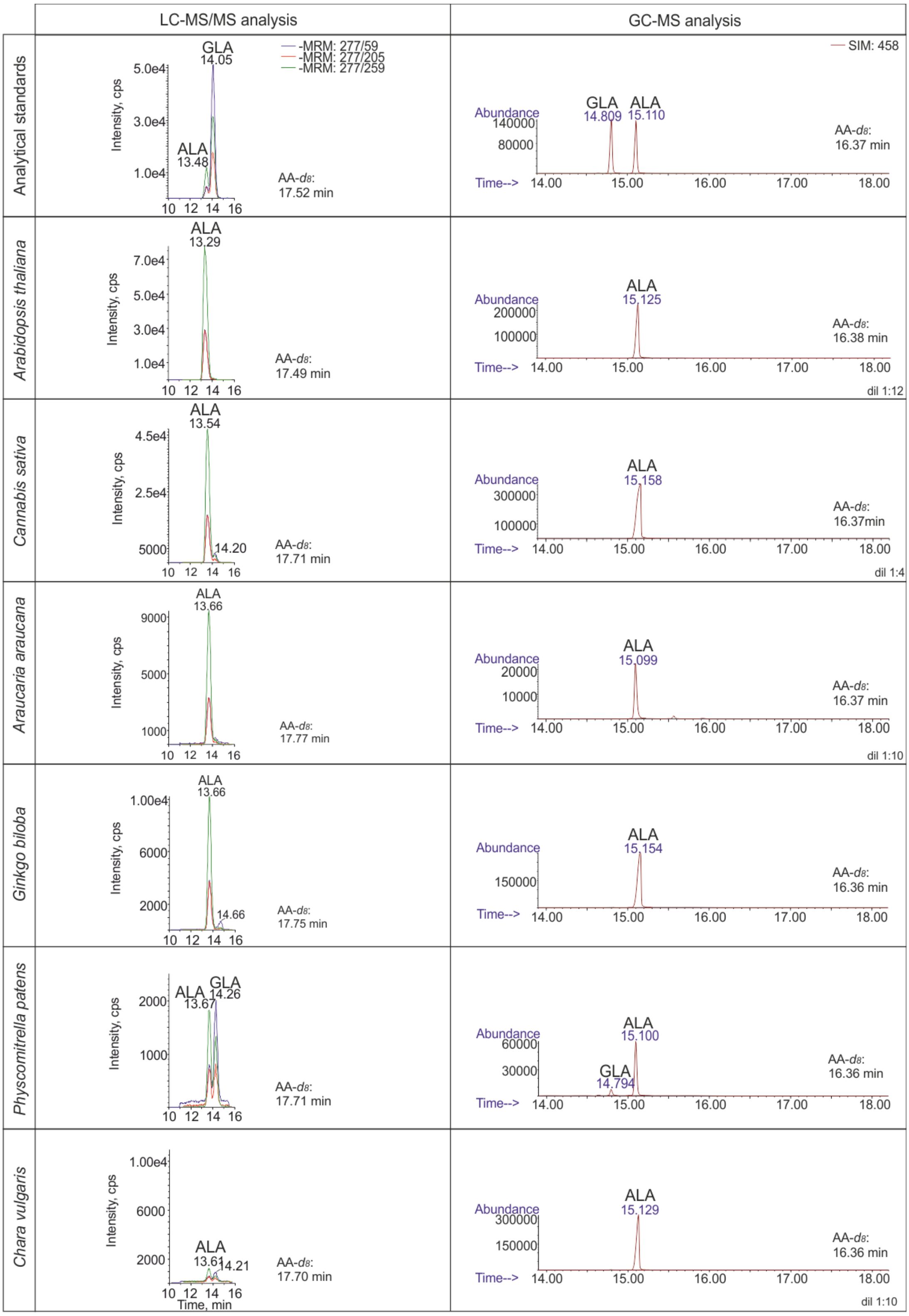

Supplementary Fig. S7. Chromatograms showing the analysis of ALA and GLA by LC-MS/MS (left row) and GC-MS (right row). Samples were prepared using HPLC for cleanup (see Sample preparation for identification) and analyzed using LC-MS/MS (see Chromatographic conditions used for the identification of structural isomers (LC-MS/MS method 2)) and GC-MS (see GC-MS analysis).

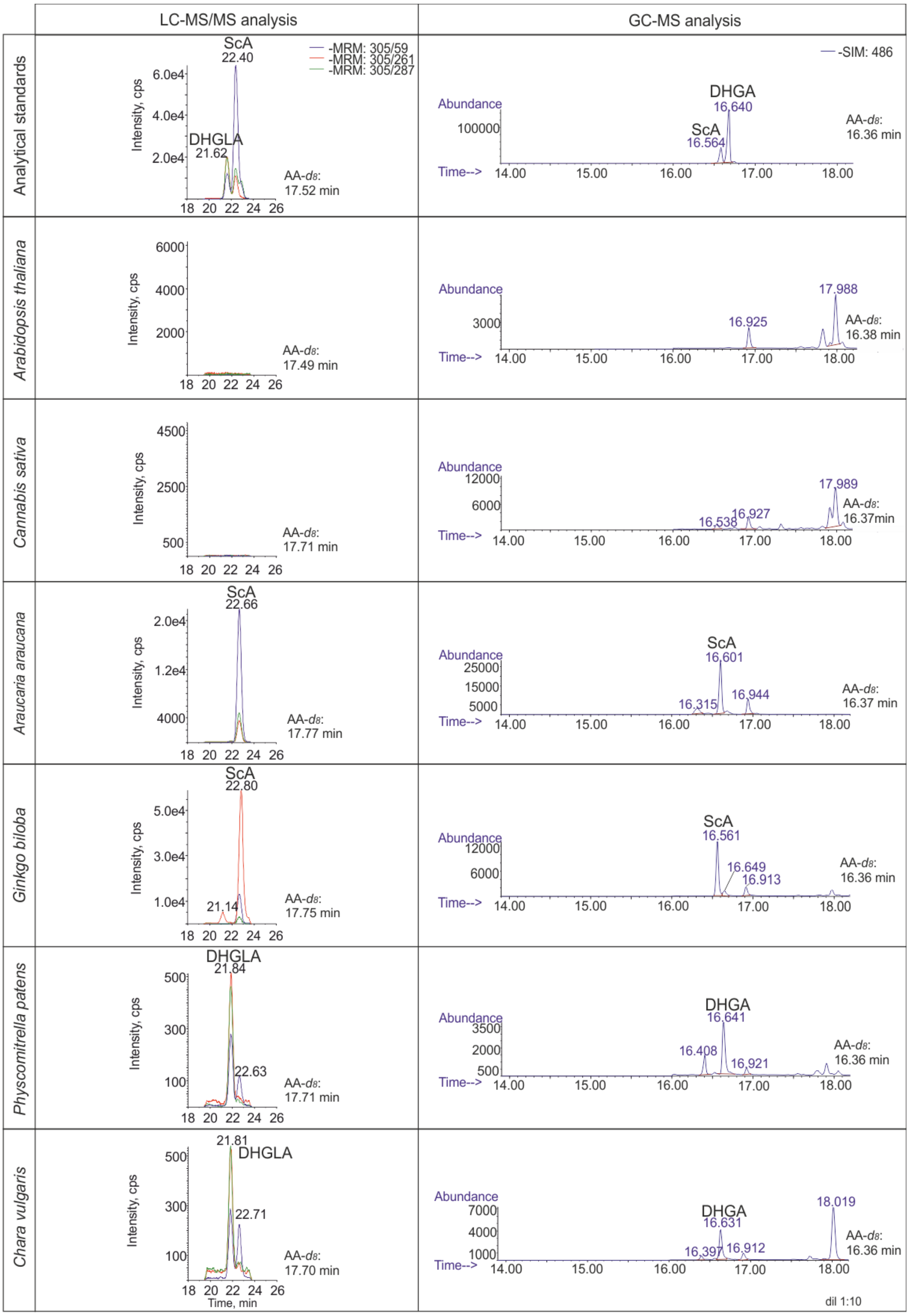

Supplementary Fig. S8. Chromatograms showing the analysis of ScA and DHGLA by LC-MS/MS (left row) and GC-MS (right row). Samples were prepared using HPLC for cleanup (see Sample preparation for identification) and analyzed using LC-MS/MS (see Chromatographic conditions used for the identification of structural isomers (LC-MS/MS method 2)) and GC-MS (see GC-MS analysis).

References:

1. Gachet, M. S., Rhyn, P., Bosch, O. G., Quednow, B. B. & Gertsch, J. A quantitiative LC-MS/MS method for the measurement of arachidonic acid, prostanoids, endocannabinoids, N-acylethanolamines and steroids in human plasma. *Journal of Chromatography B* **976-977,** 6–18 (2015).
2. Gachet, M. S. & Gertsch, J. Quantitative analysis of arachidonic acid, endocannabinoids, N-acylethanolamines and steroids in biological samples by LCMS/MS: Fit to purpose. *Journal of Chromatography B* (2015). doi:10.1016/j.jchromb.2015.11.013
3. Marazzi, J., Kleyer, J., Paredes, J. M. V. & Gertsch, J. Endocannabinoid content in fetal bovine sera - unexpected effects on mononuclear cells and osteoclastogenesis. *Journal of immunological methods* **373,** 219–228 (2011).
